# Healable Supramolecular Polyurethane Elastomers Possessing Pendant Bis-Aromatic Urea Recognition Units for Use in Repairable Coatings

**DOI:** 10.1021/acsapm.4c03135

**Published:** 2024-12-05

**Authors:** Adam D. O’Donnell, Matthew Hyder, Ann M. Chippindale, Josephine L. Harries, Ian M. German, Wayne Hayes

**Affiliations:** † Department of Chemistry, University of Reading, Whiteknights, Reading RG6 6DX, United Kingdom; ‡ Domino UK Ltd, Trafalgar Way, Bar Hill, Cambridge CB23 8TU, United Kingdom; § Kinectrics UK Ltd, 17-18 Frederick Sanger Road, The Surrey Research Park, Guildford, Surrey GU2 7YD, United Kingdom

**Keywords:** Polyurethane, Supramolecular, Hydrogen bonding, Healable, Pendent groups

## Abstract

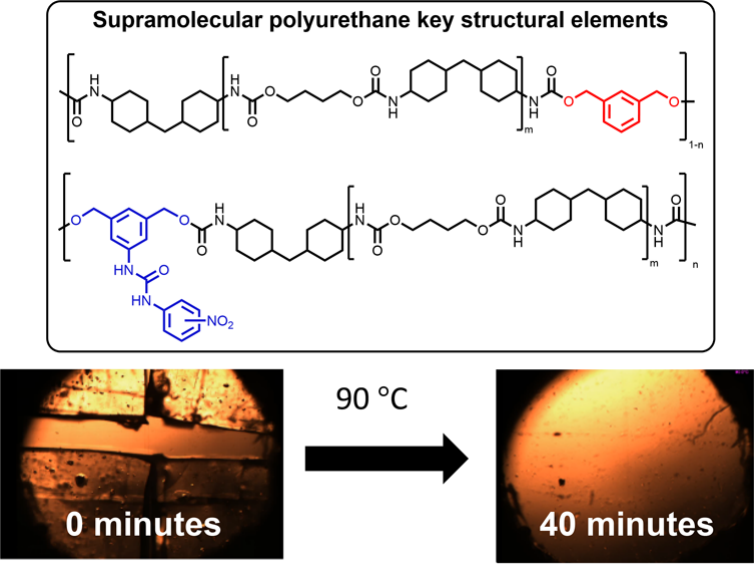

This paper describes the synthesis, characterization, and supramolecular assembly of polyurethane elastomers. Bis-aromatic urea hydrogen-bonding motifs have been used to promote the self-assembly of the materials. The materials described comprise a soft block, namely, polytetramethylene ether glycol (PTMG), as a telechelic diol and hard crystalline domains that feature a bis-aromatic urea hydrogen bonding motif as a chain extender. Two diols were polymerized (one featuring the bis-aromatic urea hydrogen bonding motif) with a PTMG diisocyanate prepolymer to yield supramolecular polyurethanes with molecular weights *ca*. 185000 with polydispersities *ca*. 2.0. The mechanical properties and processing temperatures of the polyurethanes were shown to be tunable by controlling the feed ratio of the supramolecular chain extenders. These supramolecular polyurethanes were found to be healable in nature, offering a useful property for use of these elastomers in applications such as cable coatings.

## 1 Introduction

Supramolecular polymers are dynamic systems that utilize noncovalent interactions to assemble and deliver properties that can be comparable to those exhibited by covalently-bonded polymers.^1−3^ The advantage of using supramolecular assembly to tailor mechanical properties stems from their stimuli-responsive nature, which is not typically observed in conventional covalently bonded polymers, especially cross-linked systems. Stimuli such as temperature and light have been successfully used to modify polymer properties, and as such, supramolecular polymers are an intriguing proposition when selecting a material for a specific application. They have found application in adhesives,^4−7^ coatings,^8−10^ biomedical devices,^11,12^ and healable materials.^13−17^ Bao and co-workers reported the synthesis and mechanical-property enhancement of a series of poly(siloxane) elastomers (PDMSs) by varying the chosen isocyanate and, thus, the strength of the hydrogen-bonding interaction in the hard domains, self-healing PDMS elastomers with tunable elasticity, and toughness.^18^ Fu and co-workers described the synthesis of a chain-extended self-healing polytetramethylene ether glycol (PTMG) elastomer with a tensile strength of 29.0 MPa.^19^ In an alternative approach, Park and co-workers reported a self-healing elastomer in which a PTMG diol was chain extended by a bis(4-hydroxylphenyl) disulfide unit: recovery of the material properties was achieved through aromatic disulfide metathesis.^20^ Zhang and co-workers have recently adapted these metathesis chemistries to generate a bottlebrush polyurethane elastomer with skin-like properties that is capable of self-healing.^21^

Alternative polymer topologies of supramolecular polymer systems from linear systems end-capped with recognition units have also been investigated: for example, thermoreversible random comb copolymers composed of butyl acrylate and a UPy-functionalized methacrylate have been reported by Long and co-workers.^22^ The physical characteristics of the resultant supramolecular polyacrylates were dependent on the loading of the hydrogen-bonding UPy unit pendant from the polymer backbone, and the adhesive properties were improved via incorporation of the UPy pendant groups.

Cable coatings present as internal and external cable sheaths in all cable types from high-voltage power transmission cables to control, communication, signaling, data, and fiber optic cables perform crucial energy-supply, communication, and protection roles for modern society, where reliability and low to zero maintenance is paramount.^23^ Protection of power and fiber optic cables, in particular from environmental factors (moisture, heat, pressure, chemicals), is required, in addition to minimizing the effects from direct mechanical damage during installation and operation. There are many types of cable coating and sheathing compounds available, each possessing unique properties suitable for different applications.^24^ Elastomeric polyurethane coatings provide excellent resistance to abrasion, chemicals, and weathering, rendering them suitable for use in harsh environments.^25^ In addition, these flexible elastomers offer good electrical insulation properties, making them ideal for use in electrical and electronic applications. Prolonging the operational lifetime of cables and maintaining their design performance are major objectives in the cable industry. To develop cable coating and sheathing materials capable of self-repair *in situ* without the need for intervention to effect either a repair or replacement of damage would constitute a significant development.^26,27^

As part of a program to discover novel elastomers with healing properties for use in cable coatings, this paper reports the design and preparation of a series of supramolecular polyurethane comb elastomers (SPEs), **SPE1**-**12**. Various loadings of hydrogen-bonding bis-aromatic ureas with nitro moieties located in either the *meta* or *para* positions and weaker π–π stacking interactions were introduced as pendant groups^28^ in these comb polymers to physically cross-link and tune the mechanical properties of the resultant supramolecular polymer elastomer to afford healable coating materials and thus prolong their useful operational lifespan.

## 2 Experimental Section

### 2.1 Materials

Tetrahydrofuran (THF) was distilled from benzophenone and sodium before use. All other reagents were purchased from Sigma-Aldrich and used as received. The bis acid-ureas used in this study were synthesized according to literature procedures.^29,30^

### 2.2 NMR Spectroscopy

^1^H NMR and ^13^C{H} NMR spectra were recorded on either a Bruker Nanobay 400 or a Bruker DPX 400 spectrometer operating at 400 MHz for ^1^H NMR or 100 MHz for ^13^C{H} NMR spectroscopic analysis. The data were processed using MestReNova Version 11.0.3-18688. Samples for NMR spectroscopic analysis were prepared in CDCl_3_, *d*_6_-DMSO, or *d*_8_-THF, and dissolution of the sample was aided by gentle heating. Chemical shifts (δ) are reported in parts per million relative to tetramethylsilane (δ 0.00 ppm) for CDCl_3_ and the residual solvent resonance (δ 2.50 ppm) for *d*_6_-DMSO and (δ 1.73 ppm) for *d*_8_-THF in ^1^H NMR spectra.

### 2.3 Infrared Spectroscopic Analysis

A PerkinElmer 100 FT-IR (Fourier transform infrared) instrument was employed with a diamond-ATR sampling accessory. Variable temperature IR (VT-IR) spectroscopic analysis was carried out using a PerkinElmer 100 FT-IR spectrometer with a Specac variable temperature cell holder and Temperature Controller. The temperature was measured locally with a thermocouple embedded inside the solid cell frame.

### 2.4 Mass Spectrometry

Mass spectra were obtained by use of a ThermoFisher Scientific Orbitrap XL LCMS. The sample was introduced by liquid chromatography (LC), and sample ionization was achieved by electrospray ionization (ESI).

### 2.5 Gel Permeation Chromatography

An Agilent Technologies 1260 Infinity II system was employed to determine the molecular weight and polydispersity data (the mobile phase used was HPLC-grade DMF with 5 mM NH_4_BF_4_ at a flow rate of 1.0 mL min^–1^). The system was calibrated using a series of near monodisperse polystyrene standards; toluene was used as an internal flow marker, and samples were prepared at a concentration of 1 mg mL^–1^.

### 2.6 Thermal Analysis

Differential scanning calorimetry (DSC) was performed on a Discovery DSC 25 TA Instrument. All experiments were carried out under a nitrogen atmosphere and with a heating rate of 10 °C min^–1^. Preweighed samples of 5 ± 1 mg were loaded at 25 °C, cooled to −90 °C, and heated to 200 °C. All glass-transition temperature (*T*_*g*_) values were determined from the midpoints in the second heating run using the TRIOS software (v5.1.1) of TA Instruments unless stated otherwise. Melting points were recorded using a Stuart MP10 melting point apparatus and are uncorrected.

### 2.7 X-ray Diffraction Analysis

Small-angle X-ray scattering (SAXS) and wide-angle X-ray scattering (WAXS) experiments were performed on a Bruker Nanostar. Samples were mounted in modified DSC pans equipped with Kapton windows in an MRI electrical heating unit for temperature control. In the case of single-crystal X-ray diffraction analysis, crystals of **1** and **2** were mounted under Paratone-N oil and flash cooled to 100 K under nitrogen in an Oxford Cryosystems Cryostream. Single-crystal X-ray intensity data were collected using a Rigaku XtaLAB Synergy diffractometer (Cu Kα radiation (λ = 1.54184 Å)). The data were reduced within the CrysAlisPro software.^31^ The structures were solved using the program Superflip,^32^ and all non-hydrogen atoms were located. Least-squares refinement against *F* was carried out using the *CRYSTALS* suite of programs.^33^ The non-hydrogen atoms in **1** and **2** were refined anisotropically. All of the hydrogen atoms could be located in difference Fourier maps. The positions of the hydrogen atoms attached to nitrogen and oxygen were refined with a *U*_iso_ of ∼1.2–1.5 times the value of *U*_eq_ of the parent N or O atom. The hydrogen atoms attached to carbon were placed geometrically with a C–H distance of 0.95 Å and a *U*_iso_ of ∼1.2–1.5 times the value of *U*_eq_ of the parent C atom, and the positions were refined with riding constraints. The crystal structure of **1** contains a high degree of pseudosymmetry with four molecules of **1** and one molecule of ethanol in the asymmetric unit.

### 2.8 Rheological Analysis

Rheological measurements were performed on a Malvern Panalytical Kinexus Lab+ instrument fitted with a Peltier plate cartridge and 8 mm parallel plate geometry and analyzed using rSpace Kinexus v1.76.2398 software.

### 2.9 Mechanical Measurements

Tensile tests were carried out using a Thümler Z3-X1200 tensometer at a rate of 10 mm min^–1^ with a 1 KN load cell and THSSD-2019 software. The modulus of toughness was calculated by integrating the recorded plot to give the area under the curve. The trapezium rule was applied to calculate the area between zero strain and strain at break for each sample. The error reported is the standard deviation for the three repeats for each sample.

The synthesis and characterization data for the compounds and polymers described in this paper are reported in the Supporting Information (SI) file.

### 2.10 Casting Films of Polyurethanes

The casting of the SPEs was performed as follows: the dried polymer was dissolved in a minimum volume of THF (approximately 3 mL per 1 g of polymer) at 40 °C while stirring. Once fully dissolved, the polymer solution was poured into a 10 cm × 10 cm mold with a PTFE base. The solvent was allowed to evaporate slowly over 24 h at room temperature and pressure. The mold was placed into a vacuum oven at 60 °C for 24 h, then under partial vacuum (approximately 800 mbar) at 60 °C for 24 h, and finally at 10 mbar for 24 h. The polymer film was then allowed to reach room temperature before removal from the mold.

## 3 Results and Discussion

### 3.1 Design, Synthesis, and Characterization

Elastomeric materials are under ever-increasing demands, as more strenuous applications demand greater elasticity, greater tensile strength, resilience, and increased lifespan.^34−36^ To meet these demands, an in-depth material understanding is required. Consequently, a series of supramolecular elastomers (**SPE1**-**12**) has been designed and synthesized in which the densities of the supramolecular cross-links have been increased (2.5 to 15 mol %) via condensation polymerizations. A polytetramethylene ether glycol (PTMG) block^37^ (*M*_n_ = 2000 g mol^–1^) was first reacted with 2.05 equiv of 4,4′-methylenebis(cyclohexyl isocyanate) (HMDI) using a catalytic quantity of dibutyltin dilaurate (DBTDL), followed by chain extension using 1,3-benzenedimethanol in combination with either 1-(3,5-bis(hydroxymethyl) phenyl)-3-(4-nitrophenyl)urea (**1**) or 1-(3,5-bis(hydroxymethyl) phenyl)-3-(3-nitrophenyl) urea (**2**). The bisaromatic urea motif was chosen because it can be deployed in a range of functional materials.^38−40^ Furthermore, it was anticipated that the self-assembly of the motif would not disrupt the association of the PTMG soft domains and, therefore, yield soft thermoplastic elastomers capable of self-healing.

**Scheme 1:**
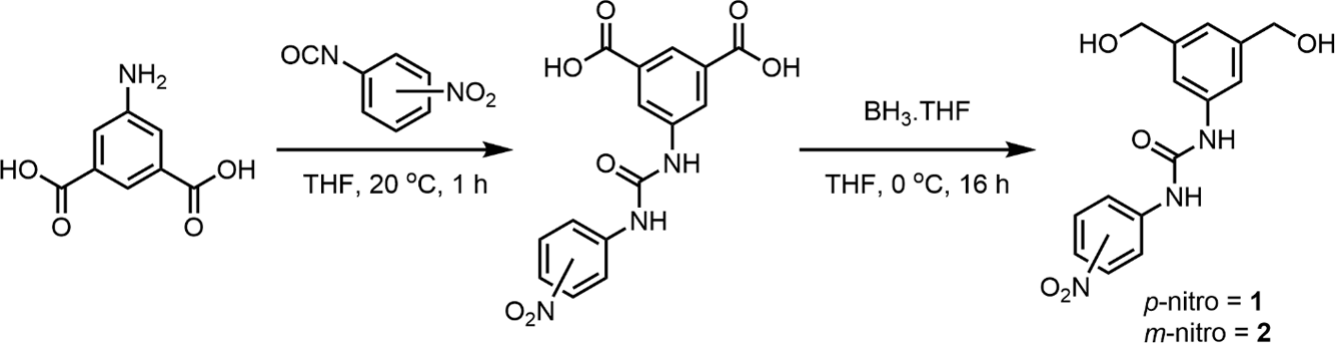
General Synthetic Route to Nitro-Aryl Urea Chain Extenders (**1** and **2**)

Synthesis of the nitro-aryl urea chain extenders **1** and **2** involved the reaction of 5-aminoisophthalic acid with the appropriate isocyanate, 3-nitrophenylisocyanate or 4-nitrophenylisocyanate, to yield known diacid ureas (Scheme 1).^29,30^ Selective borane reduction^41^ of the carboxylic acid groups yielded the respective primary alcohols **1** and **2** (see the SI file for the analytical data of these compounds). ^1^H NMR spectroscopic analysis of the diacid ureas confirmed the selective reduction through the disappearance of the resonances associated with the carboxylic acids (4.0–5.0 ppm) and the appearance of two new proton resonances at 5.20 and 4.47 ppm that were assigned to the primary alcohol and the benzylic groups, respectively (for the characterization data of **1** and **2**, see Figures S1–S9).

To gain insight into the intermolecular interactions in the hydrogen bonded domains of the resultant SPEs, single crystals of nitro-aryl urea diol chain extenders **1** and **2** were grown by slow evaporation from ethanol and studied by X-ray crystallography (see Figures S10–S13 and Tables S1–S8 for the crystallographic data for **1** and **2**). However, the presence of ethanol in the crystal of **1** complicates the packed structure, and so, only the crystal structure of **2** is described in detail in this paper. Key intermolecular interactions, established by X-ray crystallographic analysis of the crystals of **2**, are shown schematically in Figure S13B. Hydrogen-bond lengths (H···O) were 2.22(2) and 2.08(3) Å (see Table S7) for the nitro-oxygen acceptor (N13–H···O8) distance) and the urea-carbonyl N3–H···O1 distance), respectively, indicating that the urea-carbonyl groups form shorter, stronger hydrogen bonds when compared to those involving the nitro moiety. Interestingly, urea–urea bifurcation was not observed in the solid-state structure, presumably on account of competitive hydrogen bonding involving the nitro substituent and the additional hydrogen-bonding interactions between the hydroxyl groups in adjacent molecules (O(18)–H···O(22), 1.91(2) Å and O(18)′···O(22), 1.82(2) Å) shown in Figure S12.

A two-step prepolymer synthesis was performed whereby PTMG (*M*_*n*_ = 2000 g mol^–1^) was first reacted with a slight excess of HMDI (2.05 equiv) in the presence of a catalytic quantity of DBTDL to form a reactive diisocyanate prepolymer in bulk (Scheme 2). This intermediate was diluted with anhydrous DMAc, and the chain was extended with 1,3-benzenedimethanol plus **1** or **2** (from 2.5 to 15 mol %). The SPEs generated were divided into two groups: **SPE1**-**6** that feature a nitro substituent in the *-meta* position relative to the urea and **SPE7**-**12** with the nitro substituent in the *-para* position relative to the urea (see Table 1). Structural characterization and molecular weight analysis data are reported in Figures S14–S49.

**Scheme 2:**
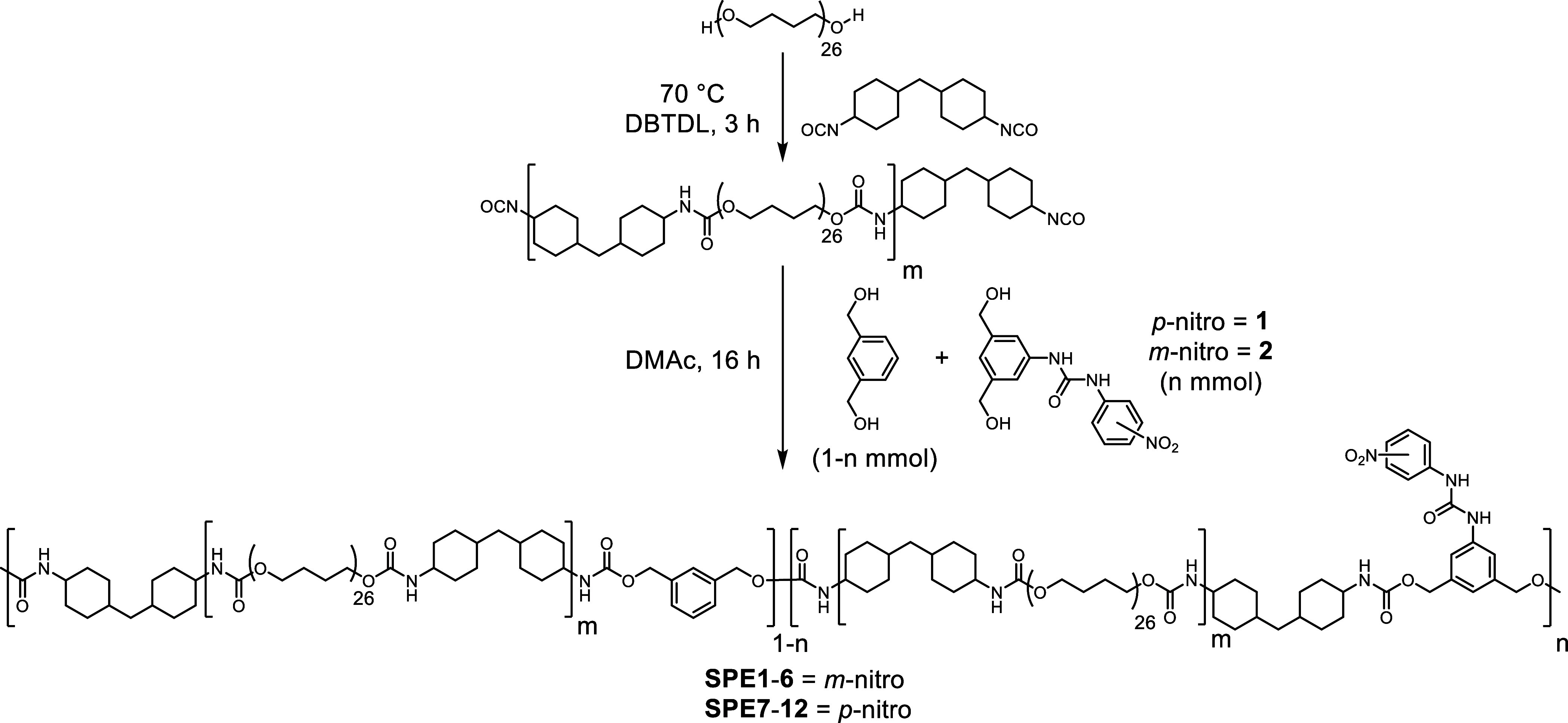
General Synthesis Strategy Employed to Synthesize **SPE1**-**12** A prepolymer is first formed by reacting PTMG with HMDI and subsequently chain extended with 1,3-benzenedimethanol and either **1** or **2**.

**Table 1:** Summary of the GPC Data for **SPE1**-**12**

SPE	mol % urea diol	*M*_*n*_/g mol^–1^	*M*_*w*_/g mol^–1^	*Đ*
**SPE1**	2.5	103200	184000	1.78
**SPE2**	5.0	82800	176000	2.13
**SPE3**	7.5	122200	232200	1.90
**SPE4**	10	101900	187800	1.84
**SPE5**	12.5	184500	408500	2.21
**SPE6**	15	101200	179000	1.76
**SPE7**	2.5	85400	150300	1.76
**SPE8**	5.0	91100	176300	1.94
**SPE9**	7.5	125800	236900	1.88
**SPE10**	10	92800	165300	1.78
**SPE11**	12.5	123800	242800	1.96
**SPE12**	15	82600	149300	1.81

^1^H NMR spectroscopic analysis revealed the successful preparation of the desired SPEs, as determined by the key resonances associated with the PTMG backbone, HMDI, and chain extenders. The proton resonances of the PTMG backbone dominate the spectra (see the ^1^H NMR spectra for **SPE1**-**12** in the SI file); however, the successful incorporation of the aromatic chain extenders was confirmed by the aromatic resonances observed at 7.33 ppm as well as the urethane resonances at 8.19 and 7.84 ppm. Furthermore, FTIR spectroscopic analysis confirmed the complete consumption of the isocyanate residues of the prepolymer (observed typically at ∼2250 cm^–1^) in the chain extension reaction prior to polymer isolation. All of the SPEs were obtained with comparable number-average molecular weight (*M*_*n*_) in the range of 83000–185000 (see the GPC eluograms of **SPE1**-**12** in the SI and Table 1) and relatively narrow polydispersity indexes (<1.5–2.21) (*Đ*, calculated by *M*_*w*_/*M*_*n*_) as measured by gel permeation chromatography (GPC) utilizing *N*,*N*-dimethylformamide (DMF) as the eluent and poly(styrene) (PS) as the calibrants.

The degree of hydrogen bonding within the supramolecular polymer networks was calculated by deconvolution of the carbonyl absorbance bands from the FT-IR spectroscopic data (see Table 2). The deconvolution analysis was carried out successfully on the urea and urethane carbonyl bands (see Figures S50–S54). Assessing the percentages of free (1692 cm^–1^), disordered hydrogen-bonded (1656–1680 cm^–1^), and ordered hydrogen-bonded (1640 cm^–1^) urea groups provides a key insight into the assembly of the SPEs; these stretching vibrations arise from the urea–urea interactions of the aromatic nitro-urea moieties pendant from the polymer backbone.^42^

**Table 2:** Normalized Percentage Integrals of the Urethane and Urea Absorbances of **SPE1**-**12** from Their Respective FT-IR Spectra, Where the Total Bound Urea Equals the Sum of Ordered Bound Urea and Disordered Bound Urea

SPE	bound urethane (%)	free urethane (%)	ordered bound urea (%)	disordered bound urea (%)	total bound urea (%)	free urea (%)
**SPE1**	100 ± 1.4		2.8 ± 0.17	14 ± 0.22	17 ± 0.27	83 ± 0.81
**SPE2**	100 ± 2.0		3.9 ± 0.22	15 ± 0.36	19 ± 0.42	81 ± 1.2
**SPE3**	100 ± 1.6		4.9 ± 0.24	18 ± 0.35	22 ± 0.43	78 ± 1.2
**SPE4**	100 ± 1.9		10 ± 0.27	18 ± 0.33	28 ± 0.42	72 ± 1.0
**SPE5**	100 ± 3.3		15 ± 0.44	18 ± 0.51	33 ± 0.67	67 ± 1.7
**SPE6**	100 ± 2.3		16 ± 0.39	22 ± 0.48	38 ± 0.62	62 ± 1.4
**SPE7**	100 ± 1.8		3.1 ± 0.14	12 ± 0.23	15 ± 0.27	85 ± 1.1
**SPE8**	100 ± 2.1		4.5 ± 0.23	15 ± 0.33	20 ± 0.41	80 ± 1.2
**SPE9**	100 ± 3.0		8.1 ± 0.36	19 ± 0.57	27 ± 0.67	73 ± 1.7
**SPE10**	100 ± 3.7		9.9 ± 0.43	21 ± 0.73	31 ± 0.85	69 ± 1.9
**SPE11**	100 ± 3.2		12 ± 0.48	22 ± 0.65	34 ± 0.80	66 ± 1.8
**SPE12**	100 ± 3.3		14 ± 0.51	23 ± 0.65	37 ± 0.83	63 ± 1.9

For both the *-para* and -*meta* nitro substituted functionalized SPEs, as the concentration of self-assembly units increased, so did the percentage of hydrogen-bonded urea, thus further validating the successful incorporation of the self-assembly units pendant from the polymeric backbone. Furthermore, the degree of hydrogen-bonded urea was found to be independent of the position of the nitro substituent on the aromatic ring. This result contrasts related studies that involved the chain end-functionalization of a poly(butadiene) backbone.^43^ In this study, analogous deconvolution of the IR spectroscopic data of the poly(butadiene) derivative did not reveal the contribution to the carbonyl absorption envelope from a free urethane stretch. As a result, the incorporation of the assembly unit pendant to the main chain of the polymer was not found to disrupt the urethane–urethane interactions along the polymeric backbone (Table 2).

### 3.2 Differential Scanning Calorimetric Analysis

The thermal properties of these supramolecular elastomers were assessed by DSC analysis (see Figures S55–S73). In the second heating cycle, the SPEs only exhibited *T*_*g*_’s at *ca*. −80 °C associated with the thermal behavior of the PTMG soft domain, and distinct glass transitions were not evident for all of the SPE materials. The thermal characteristics of all of these materials were typical of amorphous polymers in that only a single glass transition was evident, with no indication of crystal melting.

### 3.3 X-ray Scattering Analysis

**Figure 1:**
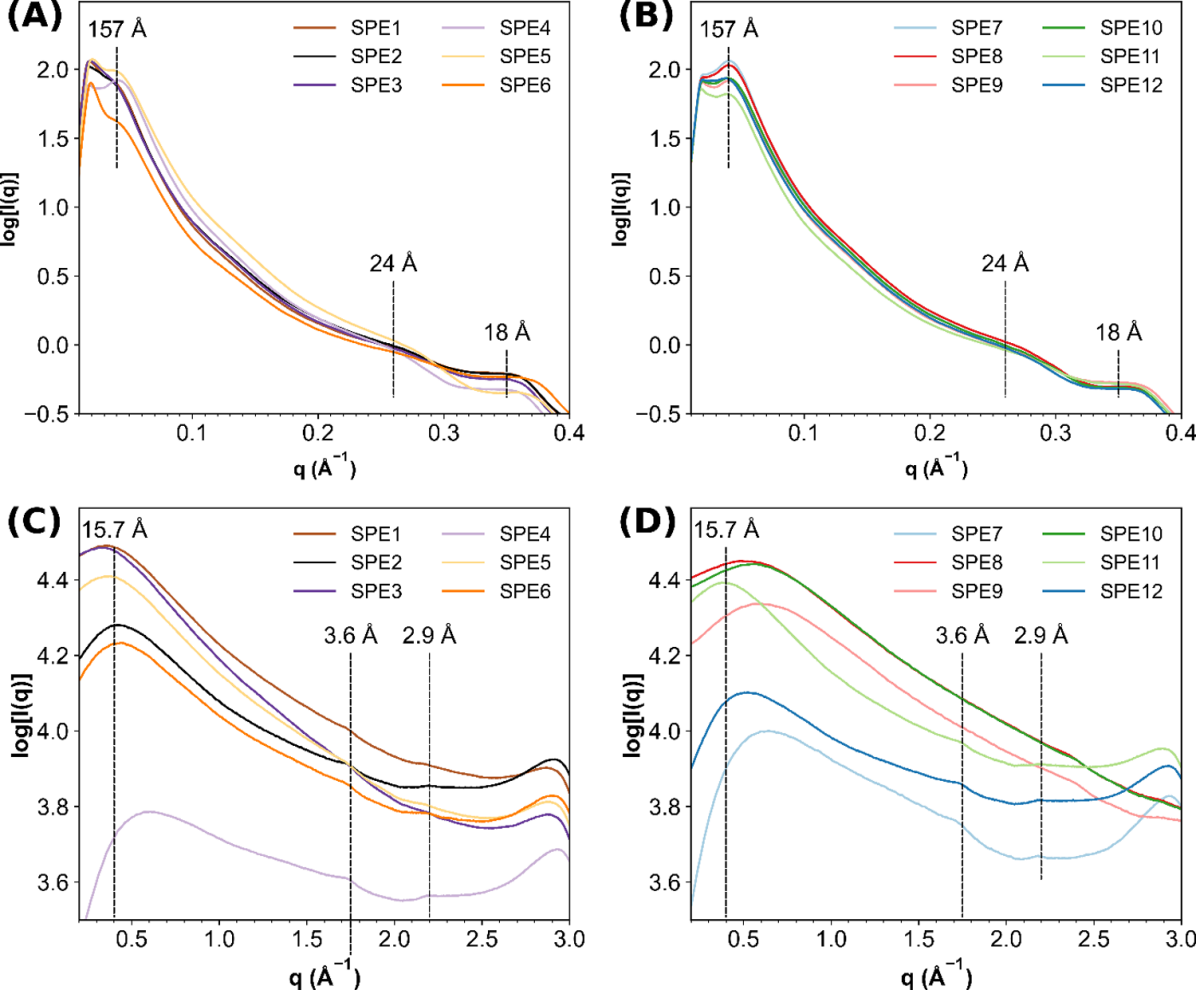
(A) SAXS intensity profiles of **SPE1**-**6**; (B) SAXS intensity profiles of **SPE7**-**12**. The data sets were acquired at room temperature. (C) WAXS intensity profiles of **SPE1**-**6**; (D) WAXS intensity profiles of **SPE7**-**12**. The data sets were acquired at room temperature.

SAXS analysis was used to investigate the phase separation in these materials.^16,44−46^ At room temperature, SAXS analysis of the bulk SPEs displayed broad Bragg scattering peaks indicative of nanophase separation between the immiscibility of the hard hydrogen-bonded chain extender and the soft PTMG backbone on the order of 157 Å. In addition, broad scattering peaks were also observed at 24 and 18 Å (Figure 1). Further insight into the assembly of the hard domains was determined by WAXS analysis, which showed broad reflections at ca. 15.7 Å. Furthermore, reflections (ca. 3.6 Å), characteristic of π–π stacking assemblies, were also evident (see Figure 1).^45^

### 3.4 Rheological Analysis

Small amplitude oscillatory shear experiments were carried out to gain a more detailed understanding of the bulk material properties of the SPEs. Oscillatory frequency sweeps were conducted between 0 and 150 °C, and time–temperature superpositions (TTS) were performed. All the SPEs exhibited a crossover frequency with an inversion of storage (*G*′) and loss modulus (*G*′′). At frequencies above the crossover, *G*′ was dominant, and the materials behaved as elastomers. Increasing the density of the nitroarylurea hydrogen bonding motifs with respect to 1,3-benzenedimethanol led to an elongated rubbery plateau and longer terminal relaxation times. Evaluation of the relaxation times of the PUs for their flow transition at different temperatures was performed through frequency sweep experiments every 10 °C from 0 to 150 °C (see Figures S74–S85).

The shear strain tolerance of the SPEs was determined by oscillatory amplitude sweeps at 1 Hz, and all subsequent frequency sweep experiments were therefore conducted at 0.1% shear strain. van Gurp–Palmen plots were utilized to confirm the validity of performing a time–temperature superposition (see Figures S86–S97). Time–temperature superposition (TTS) was performed for all the SPEs, and the curves were shifted to different reference temperatures (0, 50, 100, and 150 °C; see Figures S98–S109 for analysis of **SPE1**-**12**).

The relaxation time of the SPEs was determined by taking the reciprocal of frequency (ω) at the point of inversion of *G*′ (red) and *G*′′ (blue) (see Table 3) according to τ = 2π/ω.^47,48^ The crossover point of both *G*′ and *G*′′ is a generally accepted method to indirectly determine the average bond lifetime, τ, of supramolecular networks.^18,49^ As anticipated, the viscoelastic properties of the SPEs were found to be temperature dependent. For example, at temperatures below 30 °C, the SPEs were found to have a relaxation time on the order of days to weeks; however, upon heating to 40 °C, the relaxation time of the SPEs were found to decrease to hours. It has been shown that a bond lifetime ranging from 0.1 to 100 s is beneficial for optimum self-healing.^47,50−53^ As expected, it was found that the supramolecular bond lifetime, τ, could be tuned across the *-para* and *-meta* nitro series of SPEs. It was found that, as the concentration of the pendant supramolecular assembly moiety increased, so did the length of the rubbery plateau; this extension of the rubbery plateau is an expected consequence of increasing the strength of the interactions between polymer chains through a combination of π–π stacking interactions, plus urea-to-nitro and urea-to-urea hydrogen bonds from the pendant assembly motif. When the plateau moduli were interrogated, no significant trend in change was observed; however, a clear trend emerges in terms of supramolecular bond lifetimes (see Table 3). As the concentration of **1** or **2** increases, so does the supramolecular bond lifetime, which is a direct consequence of the interactions from the pendant assembly units. This effect is pronounced, and at 2.5 mol % loading (**SPE1**), a supramolecular bond lifetime of 1800 s is observed and an almost exponential trend becomes apparent as there are 2 orders of magnitude increase between **SPE1** and **SPE6**. Furthermore, there are 3 orders of magnitude differences between **SPE7** and **SPE12**. Evaluation of the apparent activation energies (*E*_*a*_) for polymer-chain slippage was estimated by applying an Arrhenius fit to the TTS data (see Table 3);^54^ it was found that, as the percentage of supramolecular motif increased, so did the apparent activation energy required to induce polymer slippage. For example, Arrhenius activation energies of 13.9 and 12.7 kJ mol^–1^ were estimated from the TTS data for **SPE1** and **SPE7**, respectively, when compared to those for **SPE6** and **SPE12**, which required 21.6 and 21.8 kJ mol^–1^, respectively. This observation is rationalized by increasing the concentration of the pendant hydrogen bonding units to the polymer backbone as more sites are available for self-assembly and, therefore, more energy is required to induce chain disassembly and slippage.

**Table 3:** Plateau Moduli (*G*_*p*_), Supramolecular Bond Lifetimes (τ), and *E*_a_ of Supramolecular Polymer Networks at 50 °C^a^

SPE	*G*_*p*_ (×10^5^ Pa)^b^	τ (s)	*E*_a_ (kJ mol^–1^)
**SPE1**	9.16	1800	13.9
**SPE2**	1.99	1200	13.6
**SPE3**	2.02	5500	16.7
**SPE4**	1.13	6087	18.7
**SPE5**	1.29	238000	17.9
**SPE6**	8.88	246100	21.6
**SPE7**	8.62	600	12.7
**SPE8**	8.68	1900	13.7
**SPE9**	9.63	9300	14.3
**SPE10**	2.02	72400	17.5
**SPE11**	5.07	116000	16.6
**SPE12**	2.01	279300	21.8

a Rounded to nearest 100 s.

b *G*_*p*_ is equal to the storage modulus at the minimum of the loss tangent.^55^

### 3.5 Mechanical Properties

**Figure 2:**
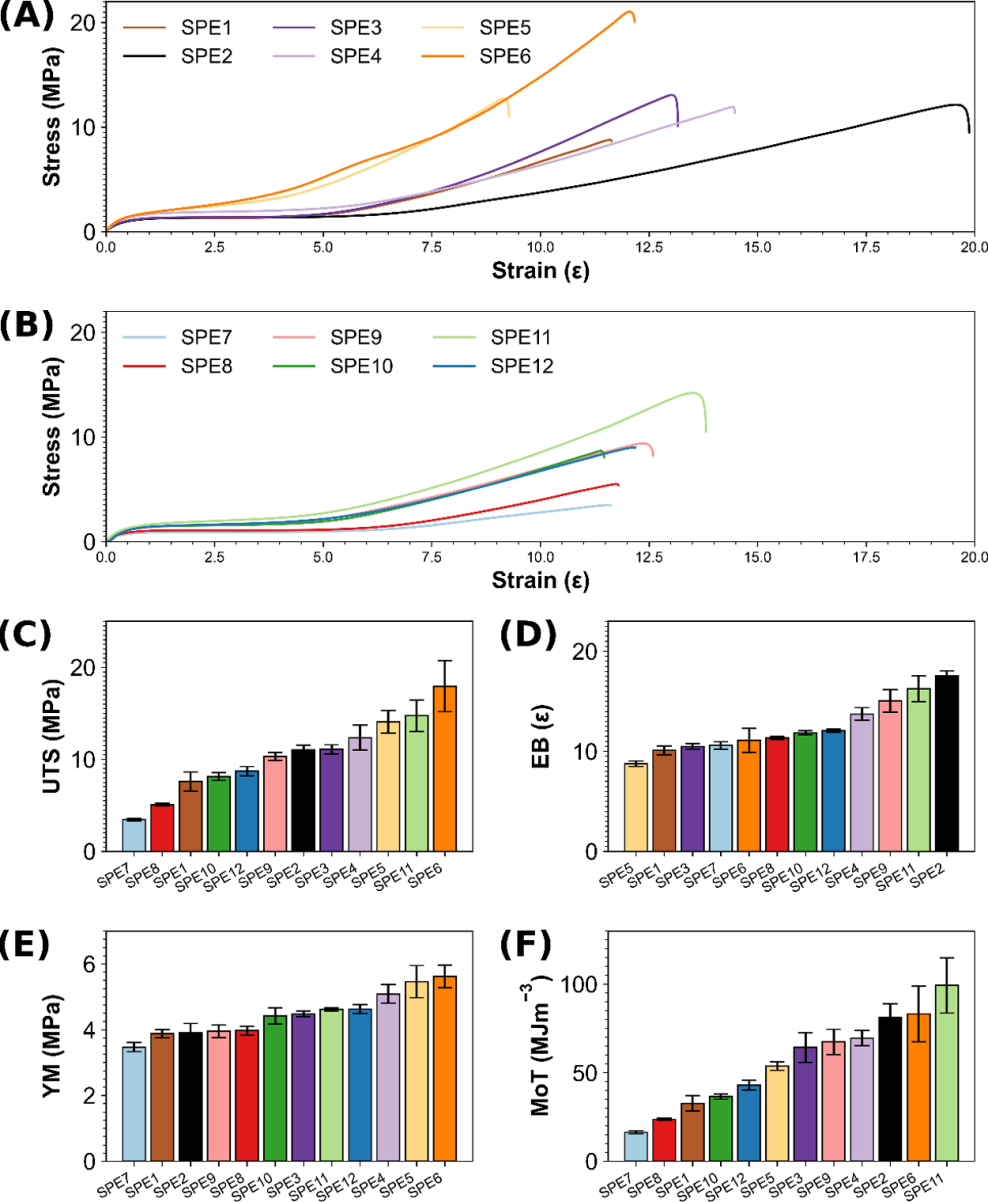
Representative stress–strain curves of supramolecular elastomers (A) **SPE1**-**6** and (B) **SPE7**-**12**. Comparison of (C) ultimate tensile strength (UTS), (D) elongation at break (EB), (E) Young’s modulus (YM), and (F) modulus of toughness (MoT). The error shown is the standard deviation of the three repeats for each sample.

The mechanical properties of the SPEs prepared in this study varied widely depending on the different amounts of hydrogen-bonding motifs pendant to the polymeric backbones (Figure 2A–F). As shown in the stress–strain curves in Figure 2A,B, increasing the amount of the aromatic nitro-urea assembly motif improved mechanical properties, such as elongation at break and ultimate tensile strength. Increasing the concentration of the self-assembly unit **2** from 2.5 mol % (**SPE1**) to 15 mol % (**SPE6**) led to a significant increase in UTS from 7.57 to 17.93 MPa. Generally, the -*meta* nitro substituted pendant groups provided SPEs with greater UTS at the same loading as the -*para* regioisomers (excluding **SPE11**), and the elongation at break shows the reverse trend (excluding **SPE2**). Furthermore, where there is usually a significant trade-off between elasticity and ultimate tensile strength in elastomeric materials, the same trend could not be applied to the comparison of **SPE1** to **SPE6**, which had comparable elongation at break values of 10.9 and 11.1, respectively. The same trend is observed for the *-para* nitro series **SPE7** to **SPE12**. The exceptional tensile strength of the polymers in this study can be attributed to the strong, highly directional assembly of the bis-aromatic urea motif pendant to the polymeric backbones.^39^ Previous studies reported by Bao and co-workers on telechelic polymer cross-linked by metal–ligand interactions showed enhanced elasticity and tensile strength and associated it with the reversible formation of intramolecular loops within the same chain and intermolecular loops between chains.^56^ A similar effect could be seen here, as the aromatic nitro-urea hydrogen bonding units self-assemble. In an unstrained state, the intrachain loops result in the folding of the polymer backbone.^56,57^

### 3.6 Self-Healing Properties

Supramolecular elastomers, which rely on noncovalent interactions such as hydrogen bonding for their mechanical properties, can be processed similarly to conventional thermoplastics as the noncovalent interactions are temperature dependent; heating and then cooling the polymer allows for the dissociation and reassociation of the supramolecular motifs.^1−3^ The proficiency of the SPEs to heal after being cut into two separate pieces was assessed by tensile testing. Rectangular specimens were cut in half with a scalpel, immediately butted together, and placed in an oven at 90 °C for 2 h. The samples were removed from the oven and allowed to cool to room temperature over 5 h before being assessed by tensile testing. Upon comparing the pristine tensile data to the tensile data for the samples which were cut in half and healed, it was found that healing efficiencies were, on average, greater than 90% for the SPEs chain extended with **1** (i.e., the nitro substituent in the *-para* position) and in some cases even exceeded 100% (see Table 4 and Figures S110–S121).

**Table 4:** Healing Data for the SPEs^a^

end-group	healed UTS (MPa)	healed EB (ε)	healed Young’s modulus (MPa)	healed modulus of toughness (MJm^–3^)
**SPE1**	7.57/5.38/71	10.09/12.09/120	3.88/3.68/95	32.61/23.66/73
**SPE2**	11.05/8.22/74	17.53/10.57/60	3.90/4.31/111	81.23/33.70/41
**SPE3**	11.08/9.34/84	10.47/11.98/114	4.48/4.19/94	64.25/39.98/65
**SPE4**	12.36/7.88/64	13.72/13.87/101	5.09/3.69/73	69.49/41.35/60
**SPE5**	14.09/9.13/65	8.75/8.30/95	5.46/5.13/94	53.71/31.49/59
**SPE6**	17.93/12.68/71	11.10/9.15/82	5.63/6.01/107	83.15/45.29/54
**SPE7**	3.44/3.25/94	10.39/9.58/92	3.47/3.30/95	16.29/14.43/89
**SPE8**	5.08/5.11/101	10.16/10.62/105	3.97/3.47/87	23.64/22.06/93
**SPE9**	9.50/10.87/115	14.11/13.03/92	3.95/3.75/95	59.50/56.24/95
**SPE10**	7.81/8.69/111	10.69/12.28/115	4.49/3.84/85	33.96/41.71/123
**SPE11**	14.73/8.29/56	14.56/10.09/69	4.68/4.85/104	99.29/36.28/37
**SPE12**	8.71/9.57/110	10.82/10.73/99	4.41/4.90/111	43.02/43.19/100

a The order of data in the table for each entry is as follows: pristine SPE/healed SPE/% healing. The samples were analyzed in triplicate.

Healing efficiencies greater than 100% are a well-known phenomenon observed in self-healing hydrogen-bonded materials and is usually a case of the material having not reached an energetic minimum prior to testing of the pristine material.^58,59^ Assessing the first *-meta* nitro series of SPEs (**SPE1**-**6**) (see Figure S122), modest recoveries were observed in terms of UTS, ranging from 64% to 84% recovery of tensile strength. In terms of elongation at break, **SPE1** recovered 120% of the pristine elasticity, and the remaining SPEs ranged from 60% to 114% in terms of recovery. When the *-para* nitro series (**SPE7**-**12**) (see Figure S123), at only 2.5 mol % loading (**SPE7**), were assessed, healing efficiencies for UTS, EB, YM, and MoT ranged from 89% to 95% and at higher loading ranged from 99% to 111% in terms of recovery of mechanical properties. Gratifyingly, when the loading of the supramolecular motif was 15 mol % (**SPE12**), an appreciable drop off in healing efficiency was not observed because of the rigidity imposed by a higher density of pendant hydrogen bond motifs.^60^ A comparison between **SPE6** and **SPE11** to examples of recently published self-healing polyurethanes shows that our SPEs outperform many in terms of UTS and EB both within their pristine and healed states; see Figure S124.^61−69^

**Figure 3:**
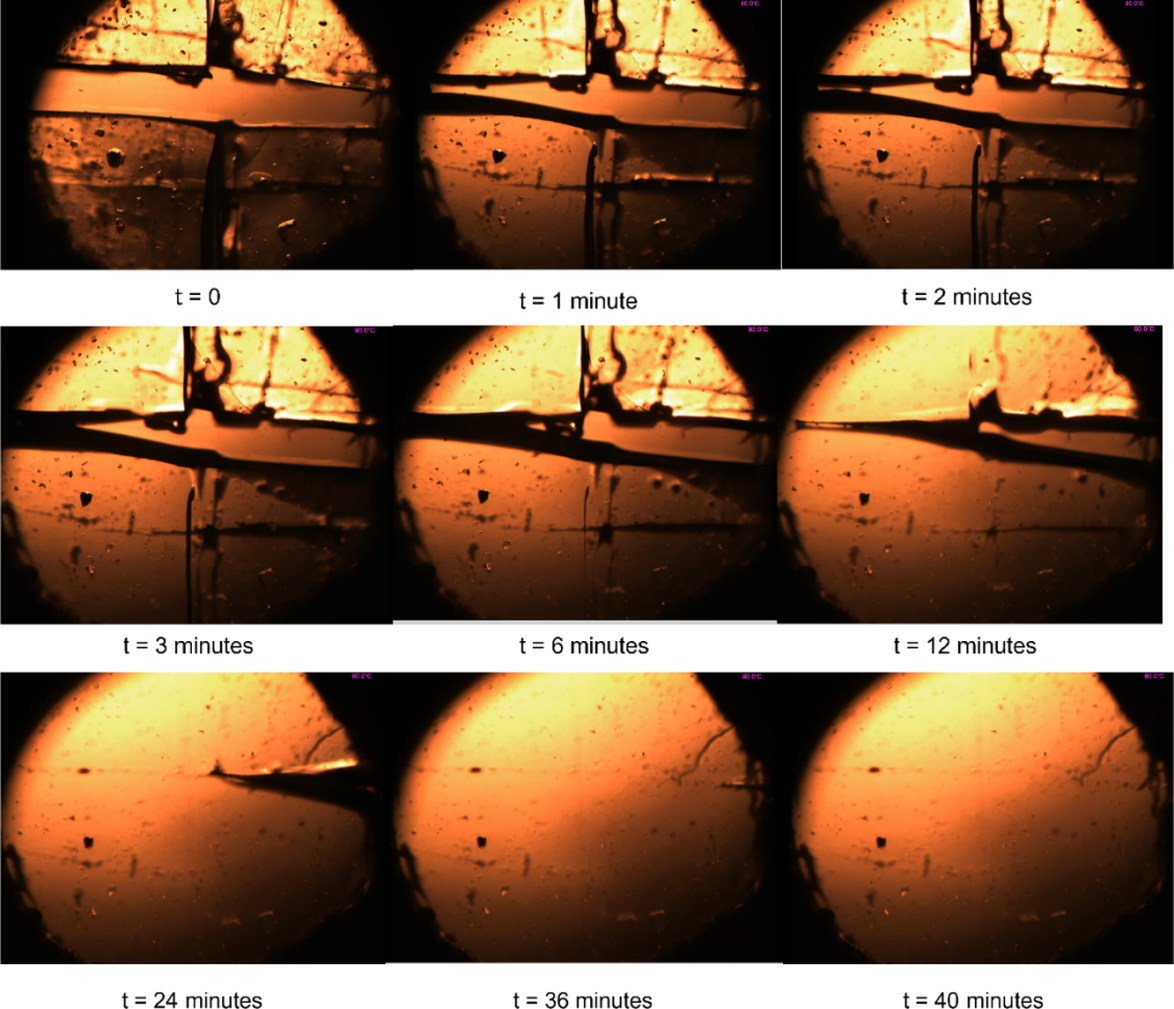
Variable-temperature hot-stage microscopy of **SPE1**. At *t* = 0, a crosscut was applied with a scalpel; the hot stage was then heated to 90 °C, and almost complete disappearance of the cut was observed within 40 min, with only a faint score mark observable on the surface.

Variable temperature optical microscopy was utilized to visualize the healing process in real-time (see Figure 3). **SPE1** was divided into four pieces by cutting a cross into the polymer film before placing it on the hot-stage microscope stage. The hot stage was rapidly brought up to a temperature of 90 °C, and almost immediately (*t* = 1 min), the supramolecular elastomer began to flow and fill the void space between the cut component. Between 3 and 12 min, the polymeric material started to “zipper” up, and at ∼24 min, the polymeric material was almost completely healed. After 40 min, a faint scratch remains where the elastomer was previously cut into four pieces, consistent with other healing studies involving supramolecular polyurethanes.^70^

The adhesive shear strength of **SPE12** was tested via lap shear adhesion tests on aluminum substrates. Lap shear samples were clamped and placed in an oven at 90 °C for 2 h, and the samples were removed from the oven and allowed to cool to room temperature over 5 h before being assessed. **SPE12** exhibited a lap shear strength of 2.91 ± 0.16 MPa, exemplifying the ability of these SPEs for use as coatings; see Figure S125.

## 4 Conclusions

This paper described a rational approach to varying the physical properties of supramolecular elastomer by increasing the hydrogen-bonding density within the material by successfully incorporating two supramolecular motifs pendant to the backbone of PTMG polyurethane through a simple two-step polyurethane synthesis process. Detailed rheological analysis revealed that the average supramolecular bond lifetime could be tuned over 3 orders of magnitude by increasing the concentration of the supramolecular motif from 2.5 to 15 mol %. Furthermore, polymer-chain slippage was evaluated, and the required energy, as determined by Arrhenius plots, increased with increasing concentration of the pendant assembly moieties. Remarkably different mechanical properties were achieved by tuning the strength of the noncovalent assembly. The mechanical properties were easily attenuated by increasing the percentage of the pendant supramolecular motif relative to a nonfunctionalized chain extender. The Young’s moduli of the materials synthesized were found to increase in a linear fashion with respect to the increasing percentage of the strongly associated bis-aryl urea pendant chains because of the formation of more hydrogen-bonding interactions. The UTS of the *-para* nitro series increased from 3.44 ± 0.12 at 2.5 mol % loading to 9.50 ± 0.77 MPa at 7.5 mol %, and at higher loading, there appeared to be diminishing returns; at 15 mol %, an UTS of 8.71 ± 0.50 was achieved. Typically, there is a trade-off between UTS and elongation at break in supramolecular elastomers; however, in these studies, it was found that, regardless of the percentage loading of the pendant supramolecular motif, the elastomers exhibited an elongation at break ranging between 10.16 ± 0.23 and 14.56 ± 0.58 times its original length. Furthermore, the ability of the supramolecular elastomers to heal was assessed, and at only 2.5 mol % loading, healing efficiencies ranged from 89% to 95%; at higher loading, the recovery of mechanical properties ranged from 99% to 111%. Comparing both regioisomers at the same loading, a trend was evident: to create materials with higher UTS, *-meta* nitro substitution is preferred; however, if elasticity is the desired mechanical property and higher healing efficiency is required, then *-para* nitro substitution delivers materials with improved performance and greater elongation to break. Further refinement of such supramolecular elastomers offers a route to novel materials suited for use in applications such as cable coatings.

## Data Availability

Data available upon request.

## Supporting Information Available

Additional experimental details and methods, including spectroscopic characterization of the polyurethanes, GPC data, crystallographic data, and rheology data (PDF)

## Accession Codes

The supplementary crystallographic data for **1** (CCDC 2091253) and **2** (CCDC 2254715) can be obtained free of charge via www.ccdc.cam.ac.uk/data_request/cif, or by emailing data_request@ccdc.cam.ac.uk, or by contacting The Cambridge Crystallographic Data Centre, 12 Union Road, Cambridge CB2 1EZ, UK; fax: + 44 1223 336033.
