# Supplementary material for: Healable Supramolecular Polyurethane Elastomers Possessing Pendant Bis-Aromatic Urea Recognition Units for Use in Repairable Coatings
Source: ACS Appl Polym Mater. 2024 Dec 5;6(24):15242–52. doi: 10.1021/acsapm.4c03135 (PMC11686464; doi:10.1021/acsapm.4c03135)
Supplement: Supplementary file 1 — ap4c03135_si_001.pdf [file ap4c03135_si_001.pdf]

SUPPORTING INFORMATION

Healable supramolecular polyurethane elastomers possessing pendant bis-aromatic urea recognition units for use in repairable coatings

Adam D. O'Donnell,[a] Matthew Hyder,[a] Ann M. Chippindale,[a] Josephine L. Harries,[b] Ian M. German,[c] and Wayne Hayes[a,*]

[a]Department of Chemistry, University of Reading, Whiteknights, Reading, RG6 6DX, U.K.

[b]Domino UK Ltd, Trafalgar Way, Bar Hill, Cambridge, CB23 8TU, U.K.

[c]Kinectrics Inc., 17-18 Frederick Sanger Road, The Surrey Research Park, Guildford, Surrey, GU2 7YD, U.K.

*Corresponding author: email address: w.c.hayes@reading.ac.uk

Table of Contents

Synthesis of bis-benzylic alcohols 1 and 2. ....S6

General synthesis of SPEs...S6

Figure S 1 ^{1}H NMR spectrum of compound **1** S9

Figure S 2 ^{13}C{H} NMR spectrum of compound **1** S9

Figure S 3 ^{1}H-^{1}H COSY analysis of compound **1** S10

Figure S 4 HMBC analysis of compound **1**...S10

Figure S 5 HRMS analysis of compound **1**...S11

Figure S 6 ^{1}H NMR spectrum of compound **2** S11

Figure S 7 ^{13}C{H} NMR spectrum of compound **2** S12

Figure S 8 ^{1}H-^{1}H COSY analysis of compound **2** S12

Figure S 9 HRMS analysis of compound **2**...S13

Figure S 10 The asymmetric unit of **1** with ellipsoids drawn at 50% probability..........S14

Table S 1 Crystallographic details for Compound **1**...S15

Table S 2 Selected bond lengths and angles for Molecules **a - e** in Compound **1**......S16

Table S 3 Hydrogen-bond interactions (Å, °) for compound **1**.....................................S22

Table S 4 Intermolecular short contacts (Å) for Compound **1**. S23

Figure S 11 Asymmetric unit of Compound **2** containing one molecule of the urea obtained from single-crystal X-ray diffraction analysis showing the atom labelling scheme. Thermal ellipsoids drawn at 50% probability. S23

Table S 5 Crystallographic details for Compound **2**...S24

Table S 6 Selected bond lengths (Å) and angles (°) for Compound **2** S25

Table S 7 Hydrogen-bond interactions (Å, °) for Compound **2**....................................S26

Table S 8 Intermolecular short contacts (Å) for Compound **2**. S26

Figure S 12 Packing diagrams for Compound **2.** S27
Figure S 13 The asymmetric unit of **2** with ellipsoids drawn at 50% probability........ S28
Figure S 14 ^{1}H NMR spectrum of **SPE1** S29
Figure S 15 ^{13}C{H} NMR spectrum of **SPE1** S29
Figure S 16 GPC eluogram of **SPE1** S30
Figure S 17 ^{1}H NMR spectrum of **SPE2** S30
Figure S 18 ^{13}C{H} NMR spectrum of **SPE2** S31
Figure S 19 GPC eluogram of **SPE2** S31
Figure S 20 ^{1}H NMR spectrum of **SPE3** S32
Figure S 21 ^{13}C{H} NMR spectrum of **SPE3** S32
Figure S 22 GPC eluogram of **SPE3** S33
Figure S 23 ^{1}H NMR spectrum of **SPE4** S33
Figure S 24 ^{13}C{H} NMR spectrum of **SPE4** S34
Figure S 25 GPC eluogram of **SPE4** S34
Figure S 26 ^{1}H NMR spectrum of **SPE5** S35
Figure S 27 ^{13}C{H} NMR spectrum of **SPE5** S35
Figure S 28 GPC eluogram of **SPE5** S36
Figure S 29 ^{1}H NMR spectrum of **SPE6** S36
Figure S 30 ^{13}C{H} NMR spectrum of **SPE6** S37
Figure S 31 GPC eluogram of **SPE6** S37
Figure S 32 ^{1}H NMR spectrum of **SPE7** S38
Figure S 33 ^{13}C{H} NMR spectrum of **SPE7** S38
Figure S 34 GPC eluogram of **SPE7** S39
Figure S 35 ^{1}H NMR spectrum of **SPE8** S39
Figure S 36 ^{13}C{H} NMR spectrum of **SPE8** S40
Figure S 37 GPC eluogram of **SPE8** S40
Figure S 38 ^{1}H NMR spectrum of **SPE9** S41
Figure S 39 ^{13}C{H} NMR spectrum of **SPE9** S41
Figure S 40 GPC eluogram of **SPE9** S42
Figure S 41 ^{1}H NMR spectrum of **SPE10** S42
Figure S 42 ^{13}C{H} NMR spectrum of **SPE10** S43
Figure S 43 GPC eluogram of **SPE10** S43

Figure S 44 ^{1}H NMR spectrum of **SPE11**. S44
Figure S 45 ^{13}C{H} NMR spectrum of **SPE11** S44
Figure S 46 GPC eluogram of **SPE11** S45
Figure S 47 ^{1}H NMR spectrum of **SPE12** S45
Figure S 48 ^{13}C{H} NMR spectrum of **SPE12** S46
Figure S 49 GPC eluogram of **SPE12** S46
Figure S 50 Deconvolution analysis of IR spectroscopic data; shown for **SPE1** S47
Figure S 51 Deconvolution analysis of IR spectroscopic data; shown for **SPE6** S47
Figure S 52 Deconvolution analysis of IR spectroscopic data; shown for **SPE7** S48
Figure S 53 Deconvolution analysis of IR spectroscopic data; shown for **SPE12** S48
Figure S 54 The urea deconvolution analysis of IR spectroscopic data S49
Figure S 55 DSC analysis of **SPE5** S49
Figure S 56 DSC analysis of **SPE6** S50
Figure S 57 DSC analysis of **SPE8** S50
Figure S 58 DSC analysis of **SPE9** S51
Figure S 59 DSC analysis of **SPE10** S51
Figure S 60 DSC analysis of **SPE11** S52
Figure S 61 DSC analysis of **SPE12** S52
Figure S 62 DSC analysis of **SPE1** from -70 to 200 °C S53
Figure S 63 DSC analysis of **SPE2** from -70 to 200 °C S53
Figure S 64 DSC analysis of **SPE3** from -70 to 200 °C S54
Figure S 65 DSC analysis of **SPE4** from -70 to 200 °C S54
Figure S 66 DSC analysis of **SPE5** from -70 to 200 °C S55
Figure S 67 DSC analysis of **SPE6** from -70 to 200 °C S55
Figure S 68 DSC analysis of **SPE7** from -70 to 200 °C S56
Figure S 69 DSC analysis of **SPE8** from -70 to 200 °C S56
Figure S 70 DSC analysis of **SPE9** from -70 to 200 °C S57
Figure S 71 DSC analysis of **SPE10** from -70 to 200 °C S57
Figure S 72 DSC analysis of **SPE11** from -70 to 200 °C S58
Figure S 73 DSC analysis of **SPE12** from -70 to 200 °C S58
Figure S 74 Frequency sweeps of **SPE1** S59
Figure S 75 Frequency sweeps of **SPE2** S59

Figure S 76 Frequency sweeps of **SPE3**....................S60
Figure S 77 Frequency sweeps of **SPE4**....................S60
Figure S 78 Frequency sweeps of **SPE5**....................S61
Figure S 79 Frequency sweeps of **SPE6**....................S61
Figure S 80 Frequency sweeps of **SPE7**....................S62
Figure S 81 Frequency sweeps of **SPE8**....................S62
Figure S 82 Frequency sweeps of **SPE9**....................S63
Figure S 83 Frequency sweeps of **SPE10**....................S63
Figure S 84 Frequency sweeps of **SPE11**....................S64
Figure S 85 Frequency sweeps of **SPE12**....................S64
Figure S 86 van Gurp-Palmen plot of **SPE1**....................S65
Figure S 87 van Gurp-Palmen plot of **SPE2**....................S65
Figure S 88 van Gurp-Palmen plot of **SPE3**....................S66
Figure S 89 van Gurp-Palmen plot of **SPE4**....................S66
Figure S 90 van Gurp-Palmen plot of **SPE5**....................S67
Figure S 91 van Gurp-Palmen plot of **SPE6**....................S67
Figure S 92 van Gurp-Palmen plot of **SPE7**....................S68
Figure S 93 van Gurp-Palmen plot of **SPE8**....................S68
Figure S 94 van Gurp-Palmen plot of **SPE9**....................S69
Figure S 95 van Gurp-Palmen plot of **SPE10**....................S69
Figure S 96 van Gurp-Palmen plot of **SPE11**....................S70
Figure S 97 van Gurp-Palmen plot of **SPE12**....................S70
Figure S 98 Master curves of **SPE1**....................S71
Figure S 99 Master curves of **SPE2**....................S72
Figure S 100 Master curves of **SPE3**....................S73
Figure S 101 Master curves of **SPE4**....................S74
Figure S 102 Master curves of **SPE5**....................S75
Figure S 103 Master curves of **SPE6**....................S76
Figure S 104 Master curves of **SPE7**....................S77
Figure S 105 Master curves of **SPE8**....................S78
Figure S 106 Master curves of **SPE9**....................S79
Figure S 107 Master curves of **SPE10**....................S80

Figure S 108 Master curves of **SPE11** S81
Figure S 109 Master curves of **SPE12** S82
Figure S 110 Stress-strain curves of the pristine and healed **SPE1**. S83
Figure S 111 Stress-strain curves of the pristine and healed **SPE2**. S83
Figure S 112 Stress-strain curves of the pristine and healed **SPE3**. S84
Figure S 113 Stress-strain curves of the pristine and healed **SPE4**. S84
Figure S 114 Stress-strain curves of the pristine and healed **SPE5**. S85
Figure S 115 Stress-strain curves of the pristine and healed **SPE6**. S85
Figure S 116 Stress-strain curves of the pristine and healed **SPE7**. S86
Figure S 117 Stress-strain curves of the pristine and healed **SPE8**. S86
Figure S 118 Stress-strain curves of the pristine and healed **SPE9**. S87
Figure S 119 Stress-strain curves of the pristine and healed **SPE10**. S87
Figure S 120 Stress-strain curves of the pristine and healed **SPE11**. S88
Figure S 121 Stress-strain curves of the pristine and healed **SPE12**. S88
Figure S 122 Comparison of healing efficiencies of **SPE1**-**6**. S89
Figure S 123 Comparison of healing efficiencies of **SPE7**-**12**. S90
Figure S 124 Comparison of pristine and healed ultimate tensile strength and elongation at break for **SPE6** and **SPE11** with literature self-healing polyurethanes....................S91
Figure S 125 Lap Shear adhesion of **SPE12** on Aluminium. S91
References...S92

Synthesis of bis-benzylic alcohols 1 and 2. The bis-benzylic alcohols **1** and **2** were generated by suspending the bis acid-ureas (5.0 g, 14.48 mmol), respectively, in THF (60 mL) and cooling the solution down to 0 °C. Borane tetrahydrofuran complex solution (58 mL, 1M in THF) was then added dropwise at 0 °C. The reaction mixture was then stirred at 20 °C for 16 hours. The mixture was cooled to 0 °C, and MeOH was added until hydrogen evolution ceased. The reaction mixture was evaporated in *vacuo,* redissolved in MeOH (150 mL), evaporated to dryness and further purified by gradient flash column chromatography (5-50% EtOH: hexane) to yield the diols **1** (3.81 g, 83%), and **2** (3.31 g, 72%), respectively.

1-(3,5-Bis(hydroxymethyl)phenyl)-3-(4-nitrophenyl)urea (**1**). m.p. 225-227 °C; FTIR (ATR, cm^{-1}): 3344, 3308, 3221, 2995, 2923, 2872, 1717, 1707, 1689, 1601, 1612, 1548 , 1294. ^{1}H NMR (400 MHz, d_6-DMSO) δ: 9.42 (s, 1H), 8.94 (s, 1H), 8.23–8.14 (AA'XX' system, 2H), 7.76–7.65 (AA'XX' system, 2H), 7.32 (s, 2H), 6.93 (s, 1H), 5.20 (t, *J* = 5.7 Hz, 2H), 4.47 (d, *J* = 5.7 Hz, 4H). ^{13}C NMR (100 MHz, d_6-DMSO) δ: 151.9, 146.5, 143.1, 140.9, 138.7, 125.2, 118.7, 117.4, 115.0, 62.9. HRMS (ESI, m/z): calcd for $C_{15}H_{16}N_3O_5$ ($[M+H]^+$) 318.1084, found 318.1083.

1-(3,5-Bis(hydroxymethyl)phenyl)-3-(3-nitrophenyl)urea (**2**). m.p. 201-203 °C; FTIR (ATR cm^{-1}): 3373, 3301, 3259, 2995, 2934, 2851, 1670, 1611, 1596, 1547, 1343. ^{1}H NMR (400 MHz, d_6-DMSO) δ: 9.18 (s, 1H), 8.84 (s, 1H), 8.60 (t, *J* = 2.2 Hz, 1H), 7.81 (dd, *J* = 8.2, 2.3 Hz, 1H), 7.73–7.63 (dd, *J* = 8.2, 2.3 Hz, 1H), 7.55 (t, *J* = 8.2, 1H), 7.33 (s, 2H), 6.92 (s, 1H), 4.96 (br. s, 2H) 4.47 (appt. s, 4H). ^{13}C NMR (100 MHz, d_6-DMSO) δ: 152.4, 148.2, 143.1, 141.1, 138.9, 130.1, 124.2, 118.5, 116.2, 114.9, 112.0, 63.0. HRMS (ESI, m/z): calcd for $C_{15}H_{16}N_3O_5$ ($[M+H]^+$) 318.1084, found 318.1083.

General synthesis of SPEs Poly(tetramethylene ether) glycol (PTMG) (M_n = 2000 g/mol) was dried under vacuum at 80 °C for 2 hours. In the bulk, PTMG was mixed with 2.05 equivalents of 4,4′-methylenebis(cyclohexyl isocyanate) (HMDI) and a catalytic quantity of dibutyltin dilaurate (DBTDL) (0.01 eq.) at 70 °C for 3 hours with gentle stirring. The colourless pre-polymer was cooled to 20 °C and dissolved in anhydrous DMAc (100 mL). 1,3-Benzenedimethanol and either **1** or **2** were then dissolved in DMAc (10 mL) in the correct stoichiometry (see Table 1) and added in one portion. The reaction mixture was then stirred at 20 °C for 4 hours, at which point the infrared stretch associated with the isocyanate terminal units was no longer present. A further volume of DMAc (100 mL) was then added, and the reaction mixture was heated to 60 °C to decrease the viscosity of the polymer solution, which was then precipitated into water (1500 mL), the resultant polymer was redissolved in THF (100 mL) and precipitated into water (3 × 1000 mL) to remove excess DMAc. The polymer was then dried *in vacuo* to yield a yellow elastomeric material.

SPE 1: FTIR (ATR, cm^{-1}): 3325, 2935, 2853, 2797, 1720, 1700, 1660, 1226; ^{1}H NMR (400 MHz, $CDCl_3$) δ: 8.08 (m, 1H), 7.92 – 7.69 (m, 1H), 7.26 (m, 297H), 6.95 (m, 4H), 4.98 (m, 343H), 4.68 (m, 171H), 4.38 (m, 19H), 4.00 (m, 196H), 3.75 (m, 143H), 3.37 (m, 5803H), 2.14 (m, 140H), 1.93 (m, 240H), 1.57 (m, 7184H), 1.06 (m, 1310H). ^{13}C NMR (100 MHz, $CDCl_3$) δ: 156.0, 155.6, 137.0, 128.8, 70.6, 70.3, 66.3, 64.4, 50.5, 50.3, 47.1, 44.1, 43.0,

33.8, 33.6, 33.4, 32.7, 32.5, 32.1, 30.1, 29.7, 29.3, 28.2, 28.1, 26.5, 26.3, 25.9. GPC (DMF) M_n = 184000 g mol^{-1}, M_w = 103200 g mol^{-1}, Đ = 1.78.

SPE 2: FTIR (ATR, cm^{-1}): 3328, 2931, 2854, 2801, 1720, 1700, 1660, 1226; ^{1}H NMR (400 MHz, $CDCl_3$) δ: 8.26 (m, 1H), 7.85 (m, 1H), 7.32 (m, 152H), 6.98 (m, 5H), 5.06 (m, 117H), 4.71 (m, 110H), 4.03 (m, 87H), 3.77 (m, 63H), 3.40 (m, 2479H), 1.97 (m, 104H), 1.86 (m, 98H), 1.73–1.45 (m, 2963H), 1.32 (m, 293H), 1.13–0.74 (m, 465H). ^{13}C NMR (100 MHz, $CDCl_3$) δ: 156.0, 155.6, 137.1, 128.9, 127.9, 70.8, 70.7, 70.4, 66.4, 64.5, 50.4, 47.2, 43.1, 33.8, 33.7, 33.5, 32.8, 32.1, 30.4, 29.8, 29.4, 28.1, 26.6, 26.3, 26.0. GPC (DMF) M_n = 82800 g mol^{-1}, M_w = 176000 g mol^{-1}, Đ = 2.13.

SPE 3: FTIR (ATR, cm^{-1}): 3318, 2934, 2854, 280, 1717, 1700, 1660, 1226; ^{1}H NMR (400 MHz, $CDCl_3$) δ: 8.18 (m, 1H), 7.95–7.73 (m, 2H), 7.30 (m, 60H), 6.98 (m, 1H), 5.06 (m, 48H), 4.97– 4.41 (m, 43H), 4.03 (m, 34H), 3.77 (m, 25H), 3.39 (m, 998H), 2.07–1.80 (m, 78H), 1.60 (m, 1185H), 1.41 (s, 51H), 1.23 (m, 48H), 1.15–0.77 (m, 187H). ^{13}C NMR (100 MHz, $CDCl_3$) δ: 156.0, 155.6, 137.1, 128.9, 127.9, 77.5, 77.4, 77.2, 77.0, 76.8, 70.8, 70.7, 70.4, 64.5, 50.4, 47.2, 43.0, 33.8, 33.7, 33.7, 33.5, 32.1, 29.8, 28.1, 26.8, 26.6, 26.3, 26.0. GPC (DMF) M_n = 122200 g mol^{-1}, M_w = 232200 g mol^{-1}, Đ = 1.90.

SPE 4: FTIR (ATR, cm^{-1}): 3338, 2935, 2853, 2802, 1717, 1700, 1660, 1227; ^{1}H NMR (400 MHz, $CDCl_3$) δ: 8.18 (m, 1H), 7.84 (m, 2H), 7.47–7.10 (m, 54H), 6.98 (m, 2H), 5.06 (s, 44H), 4.98–4.39 (m, 43H), 4.03 (s, 35H), 3.78 (s, 24H), 3.40 (s, 947H), 2.10–1.89 (m, 40H), 1.61 (d, J = 5.3 Hz, 1224H), 1.31–0.80 (m, 233H). ^{13}C NMR (100 MHz, $CDCl_3$) δ: 156.0, 155.6, 137.1, 128.9, 127.9, 70.8, 70.7, 70.4, 66.4, 64.6, 47.2, 33.9, 33.8, 33.6, 32.8, 32.2, 29.8, 29.4, 28.2, 26.6, 26.4, 26.0. GPC (DMF) M_n = 101900 g mol^{-1}, M_w = 187800 g mol^{-1}, Đ = 1.84.

SPE 5: FTIR (ATR, cm^{-1}): 3326, 2925, 2853, 2797, 1717, 1702, 1660, 1226; ^{1}H NMR (400 MHz, $CDCl_3$) δ: 8.18 (s, 1H), 7.85 (m, 2H), 7.47–7.07 (1m, 16H), 6.98 (m, 4H), 5.33 (m, 2H), 5.06 (s, 88H), 4.98–4.38 (m, 89H), 4.03 (s, 67H), 3.77 (s, 47H), 3.40 (s, 2025H), 1.97 (s, 92H), 1.86 (s, 100H), 1.60 (s, 2576H), 1.32–0.69 (m, 699H). ^{13}C NMR (100 MHz, $CDCl_3$) δ: 155.9, 155.5, 137.0, 128.8, 70.6, 70.3, 66.3, 64.4, 50.5, 50.3, 47.1, 33.6, 33.4, 32.5, 32.0, 30.3, 30.1, 29.7, 29.5, 29.3, 29.3, 28.0, 26.5, 26.2, 25.9, 25.5, 22.7, 14.1, 13.6. GPC (DMF) M_n = 184500 g mol^{-1}, M_w = 408500 g mol^{-1}, Đ = 2.21.

SPE 6: FTIR (ATR, cm^{-1}): 3322, 2939, 2853, 2797, 1717, 1700, 1660, 1226; ^{1}H NMR (400 MHz, $CDCl_3$) δ: 8.19 (s, 1H), 7.84 (s, 1H), 7.46–7.21 (m, 23H), 5.07 (s, 15H), 4.97–4.44 (m, 16H), 4.04 (s, 12H), 3.79 (s, 10H), 3.41 (s, 286H), 1.98 (s, 17H), 1.61 (s, 378H), 1.46–0.65 (m, 142H). ^{13}C NMR (100 MHz, $CDCl_3$) δ: 156.3, 155.5, 137.0, 128.8, 127.9, 127.8, 127.7, 121.7, 111.7, 70.6, 70.3, 66.2, 64.4, 50.3, 47.1, 33.7, 33.4, 32.0, 29.7, 28.0, 26.5, 26.2, 25.9, 22.7, 14.1, 1.0. GPC (DMF) M_n = 101200 g mol^{-1}, M_w = 179000 g mol^{-1}, Đ = 1.76.

SPE 7: FTIR (ATR, cm^{-1}): 3327, 2931, 2853, 2799, 1717, 1700, 1660, 1226; ^{1}H NMR (400 MHz, $CDCl_3$) δ: 8.63 (s, 1H), 8.10 (m, 1H), 7.52 (s, 2H), 7.28 (m, 249H), 6.97 (d, J = 23.8Hz, 5H), 5.03 (s, 197H), 4.98–4.44 (m, 202H), 4.01 (s, 149H), 3.75 (s, 106H), 3.37

(s, 4547H), 2.10 (s, 106H), 1.93 (s, 189H), 1.58 (s, 5484H), 1.43–1.19 (m, 461H), 1.18–0.71 (m, 929H). ^{13}C NMR (100 MHz, $CDCl_3$) δ: 156.0, 155.6, 137.1, 137.0, 137.0, 128.8, 127.8, 127.7, 127.7, 125.5, 70.8, 70.7, 70.6, 70.5, 70.3, 66.4, 64.4, 50.5, 50.3, 47.1, 47.0, 44.1, 43.9, 43.0, 41.7, 33.8, 33.7, 33.5, 33.4, 32.7, 32.6, 32.1, 31.9, 30.4, 30.1, 29.7, 29.6, 29.5, 29.3, 28.1, 26.7, 26.5, 26.3, 26.0, 25.9. GPC (DMF) M_n = 85400 g mol^{-1}, M_w = 150300 g mol^{-1}, Đ = 1.76.

SPE 8: FTIR (ATR, cm^{-1}): 3327, 2929, 2853, 2800, 1717, 1700, 1660, 1226; ^{1}H NMR (400 MHz, $CDCl_3$) δ: 8.59 (s, 1H), 8.18–7.93 (m, 3H), 7.52 (m, 164H), 6.97 (m, 5H), 5.04 (s, 135H), 4.95–4.47 (m, 124H), 4.01 (s, 104H), 3.75 (s, 69H), 3.38 (s, 2906H), 2.09 (s, 65H), 1.94 (s, 129H), 1.58 (s, 3474H), 1.39 (s, 140H), 1.22 (s, 185H), 1.14–0.75 (m, 552H). ^{13}C NMR (100 MHz, $CDCl_3$) δ: 156.0, 155.6, 137.0, 128.8, 127.9, 127.5, 126.3, 70.9, 70.9, 70.8, 70.7, 70.5, 70.3, 66.3, 64.4, 50.5, 50.3, 47.1, 44.1, 43.0, 41.7, 33.8, 33.7, 33.5, 32.7, 32.6, 32.1, 30.4, 30.1, 29.7, 29.5, 29.3, 28.1, 26.7, 26.6, 26.3, 26.1, 26.0, 25.5, 22.7. GPC (DMF) M_n = 91100 g mol^{-1}, M_w = 176300 g mol^{-1}, Đ = 1.94.

SPE 9: FTIR (ATR, cm^{-1}): 3327, 2931, 2853, 2802, 1717, 1700, 1660, 1226; ^{1}H NMR (400 MHz, $CDCl_3$) δ: 8.14 (s, 1H), 7.53 (m, 1H), 7.39–7.15 (m, 31H), 7.07–6.88 (m, 1H), 5.07 (s, 26H), 4.97–4.44 (m, 23H), 4.03 (s, 19H), 3.78 (s, 13H), 3.40 (s, 528H), 1.96 (s, 27H), 1.61 (s, 688H), 1.34–0.84 (m, 131H). ^{13}C NMR (100 MHz, $CDCl_3$) δ: 156.1, 155.6, 137.1, 128.9, 127.9, 70.7, 70.4, 66.4, 64.5, 50.4, 47.2, 33.8, 33.7, 33.6, 32.1, 29.8, 28.1, 26.6, 26.3, 26.0. GPC (DMF) M_n = 125800 g mol^{-1}, M_w = 236900 g mol^{-1}, Đ = 1.88.

SPE 10: FTIR (ATR, cm^{-1}): 3327, 2931, 2853, 2801, 1717, 1700, 1660, 1227; ^{1}H NMR (400 MHz, $CDCl_3$) δ: 8.38 (s, 1H), 8.13 (s, 1H), 7.86 (s, 1H), 7.66–7.05 (m, 37H), 7.08–6.88 (m, 2H), 5.06 (s, 27H), 4.97–4.37 (m, 27H), 4.04 (s, 23H), 3.78 (s, 19H), 3.40 (s, 555H), 2.11–1.89 (m, 30H), 1.60 (s, 704H), 1.09 (m, 134H). ^{13}C NMR (100 MHz, $CDCl_3$) δ: 156.1, 155.6, 137.1, 128.9, 127.9, 70.7, 70.4, 66.4, 64.6, 50.6, 50.4, 47.2, 44.2, 43.1, 33.9, 33.8, 33.6, 33.6, 32.8, 32.7, 32.2, 32.1, 29.8, 29.8, 28.2, 28.1, 28.0, 26.8, 26.6, 26.5, 26.3, 26.2, 26.0. GPC (DMF) M_n = 92800 g mol^{-1}, M_w = 165300 g mol^{-1}, Đ = 1.78.

SPE 11: FTIR (ATR, cm^{-1}): 3326, 2928, 2853, 2801, 1716, 1700, 1660, 1227; ^{1}H NMR (400 MHz, $CDCl_3$) δ: 8.15 (s, 1H), 7.54 (m, 1H), 7.46–7.18 (m, 20H), 7.10–6.84 (m, 2H), 5.07 (s, 11H), 4.97–4.31 (m, 13H), 4.04 (s, 9H), 3.78 (s, 6H), 3.63 (s, 2H), 3.41 (s, 221H), 2.05–1.89 (m, 10H), 1.82–1.44 (m, 305H), 1.43–1.02 (m, 63H), 1.03–0.77 (m, 20H). ^{13}C NMR (100 MHz, $CDCl_3$) δ: 156.1, 155.6, 137.1, 128.9, 70.7, 70.4, 66.4, 64.5, 33.8, 33.6, 32.1, 29.8, 28.1, 26.6, 26.3, 26.0, 22.8. GPC (DMF) M_n = 123800 g mol^{-1}, M_w = 242800 g mol^{-1}, Đ = 1.96.

SPE 12: FTIR (ATR, cm^{-1}): 3327, 2930, 2853, 2801, 1716, 1700, 1660, 1227; ^{1}H NMR (400 MHz, $CDCl_3$) δ: 8.39 (s, 1H), 8.13 (s, 2H), 7.82 (s, 1H), 7.66–6.88 (m, 30H), 5.07 (s, 23H), 4.95–4.34 (m, 20H), 4.03 (s, 16H), 3.78 (s, 11H), 3.40 (s, 440H), 1.96 (s, 20H), 1.61 (s, 575H), 1.32–0.65 (m, 116H). ^{13}C NMR (100 MHz, $CDCl_3$) δ: 155.9, 155.9, 137.0, 137.0, 128.8, 128.8, 127.9, 70.6, 70.2, 66.3, 64.4, 50.3, 47.1, 33.6, 33.4, 32.0, 29.7, 29.7, 28.0, 26.5, 26.5, 26.2, 25.9. GPC (DMF) M_n = 82600 g mol^{-1}, M_w = 149300 g mol^{-1}, Đ = 1.81.

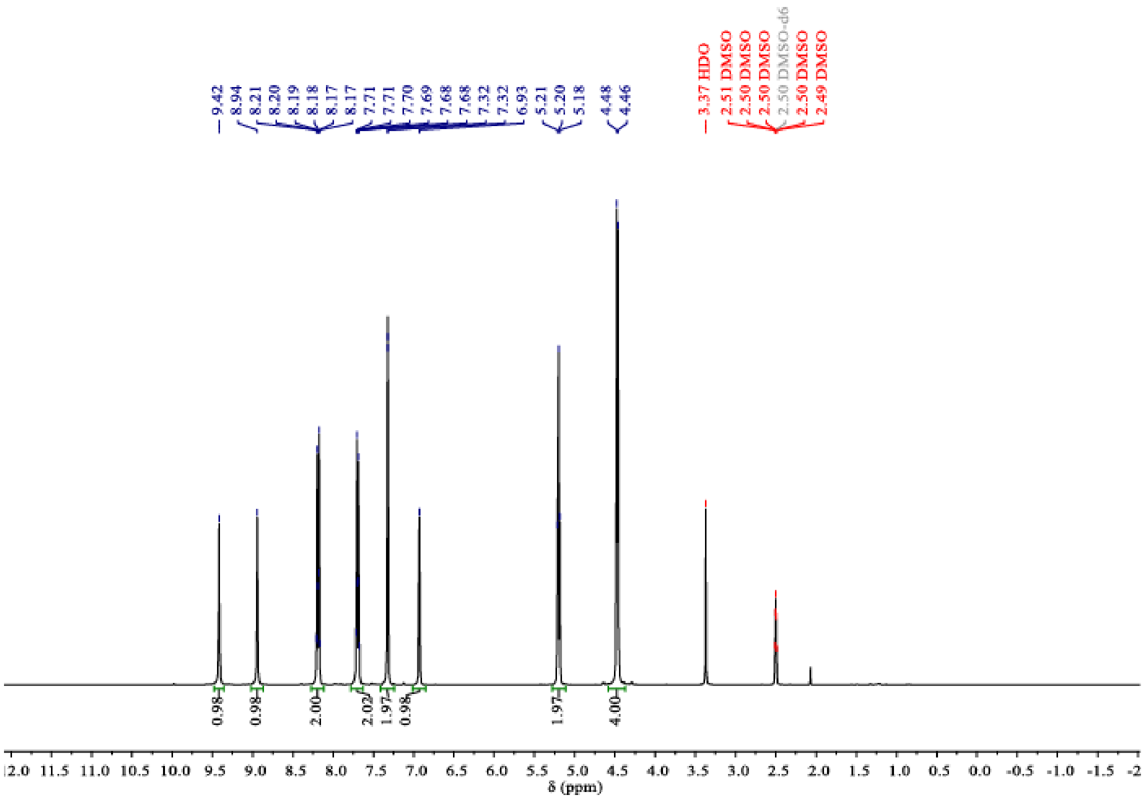

Figure S 1 ^{1}H NMR spectrum of compound **1** (400 MHz, DMSO-*d6*, 298 K).

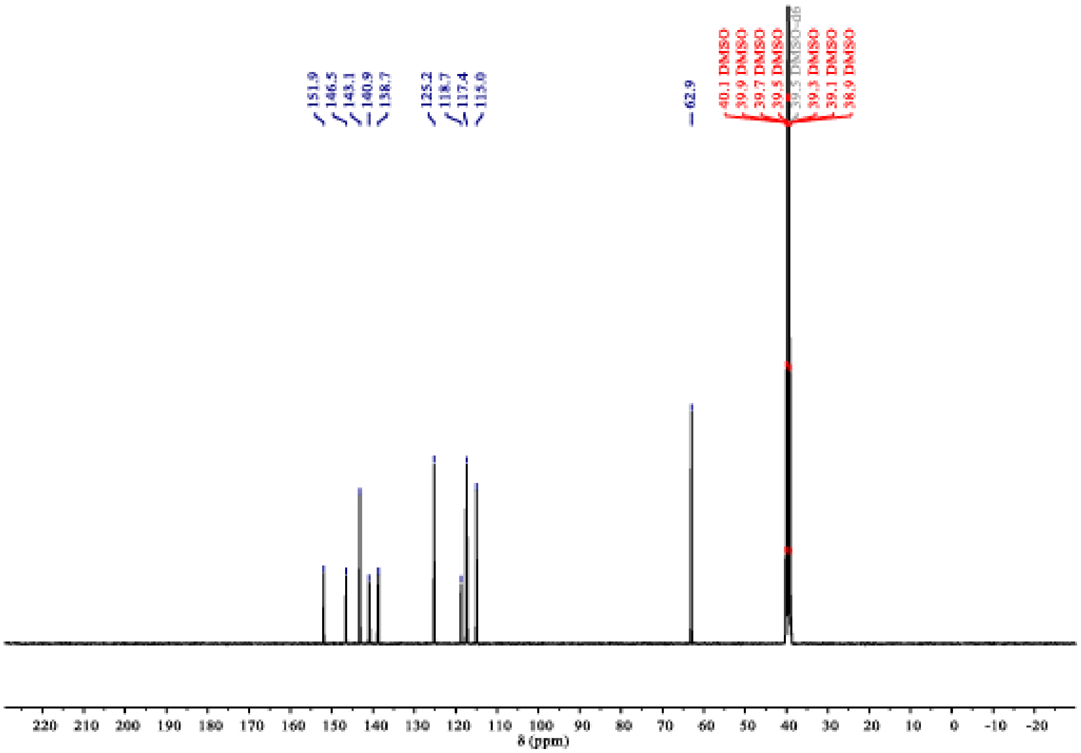

Figure S 2 ^{13}C{H} NMR spectrum of compound **1** (100 MHz, DMSO-*d6*, 298 K).

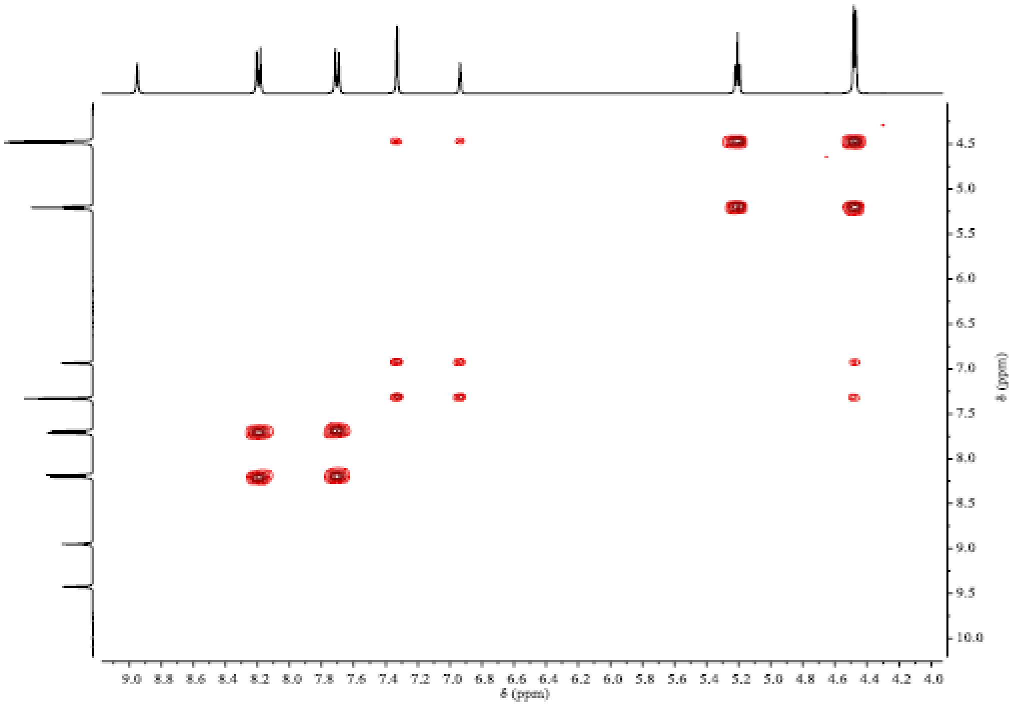

Figure S 3 ^{1}H-^{1}H COSY analysis of compound **1** (400 MHz, DMSO-*d6*, 298 K).

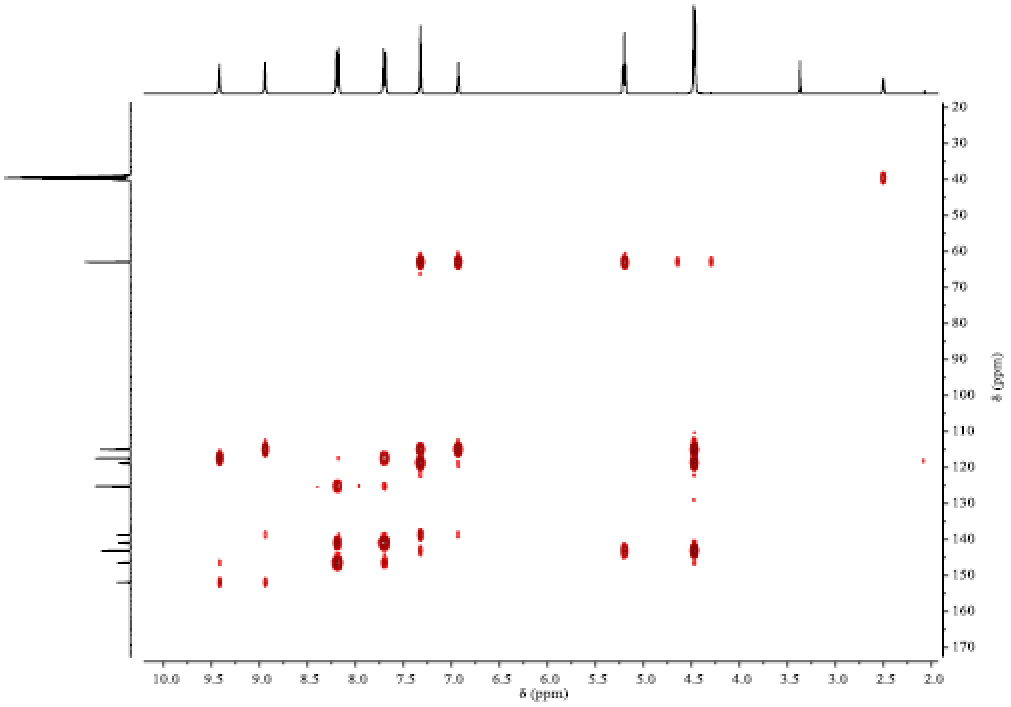

Figure S 4 HMBC analysis of compound **1** (400, 100 MHz, DMSO-*d6*, 298 K).

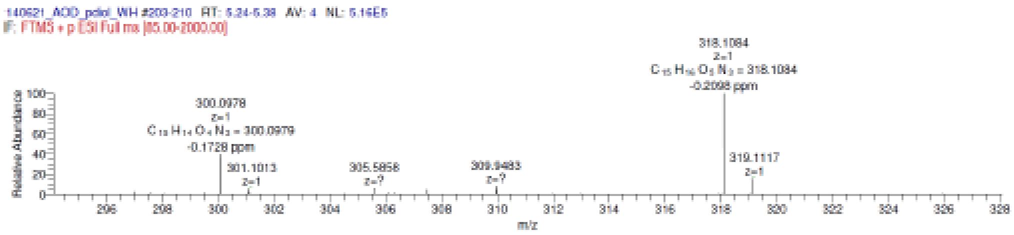

Figure S 5 HRMS analysis of compound **1**.

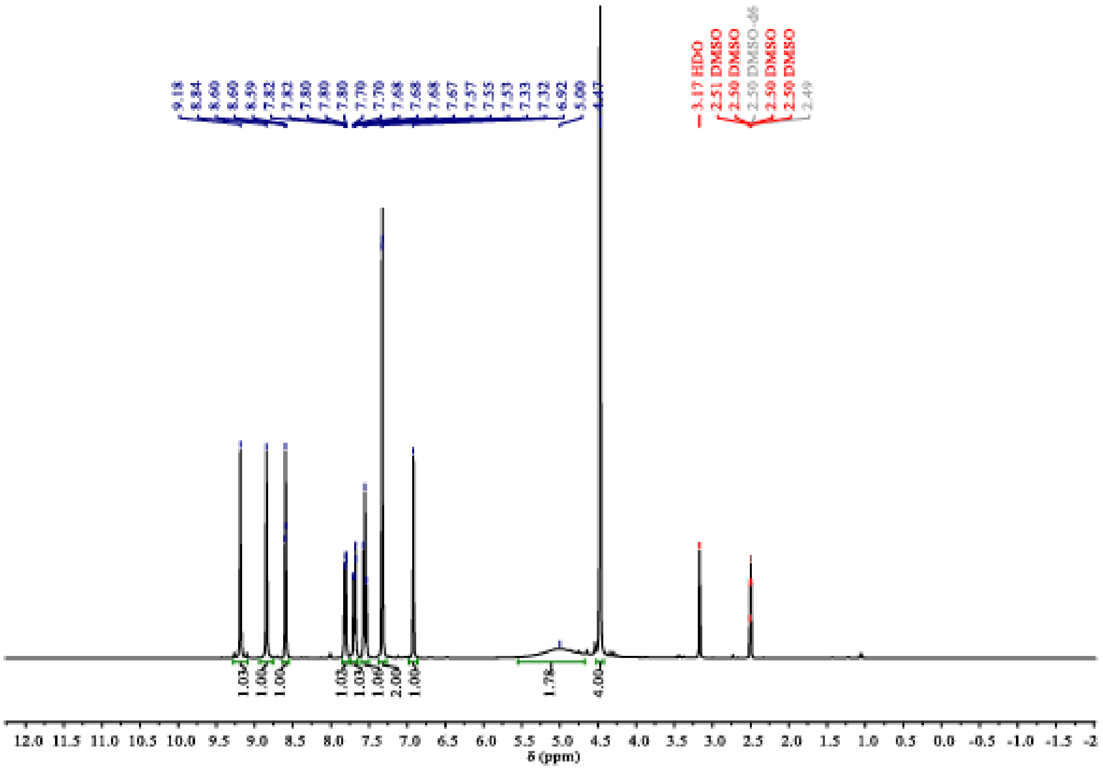

Figure S 6 ^{1}H NMR spectrum of compound **2** (400 MHz, DMSO-*d6*, 298 K).

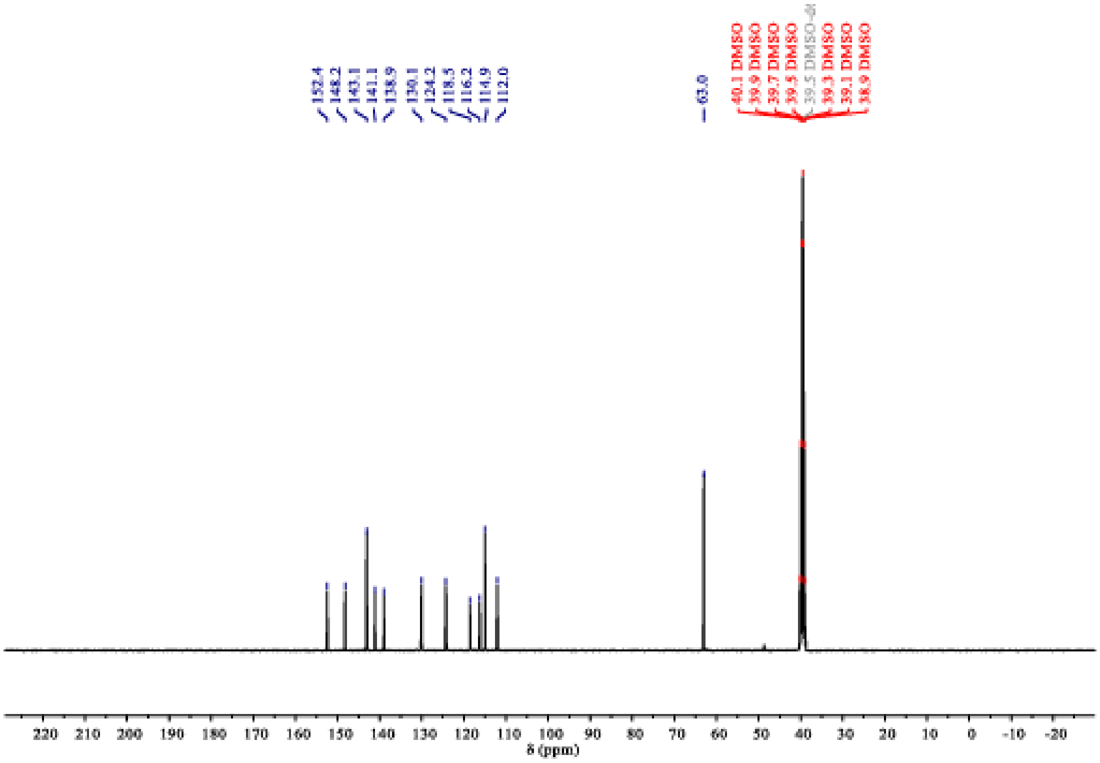

Figure S 7 $^{13}C\{H\}$ NMR spectrum of compound **2** (100 MHz, DMSO-*d6*, 298 K).

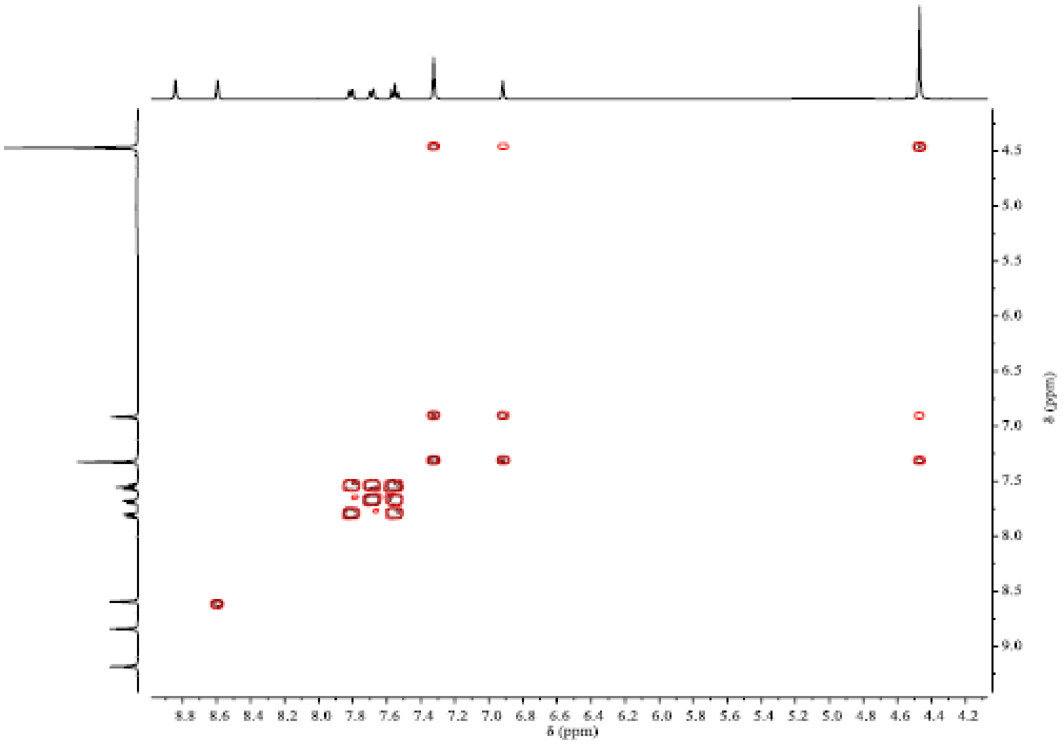

Figure S 8 ^{1}H-^{1}H COSY analysis of compound **2** (400 MHz, DMSO-*d6*, 298 K).

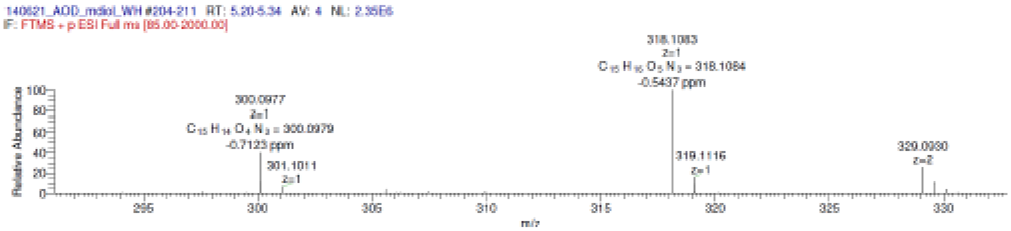

Figure S 9 HRMS analysis of compound **2**.

Crystallographic Data for Compounds 1 and 2

Compound 1:

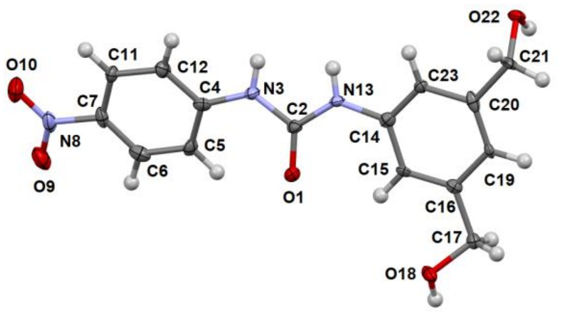

molecule a

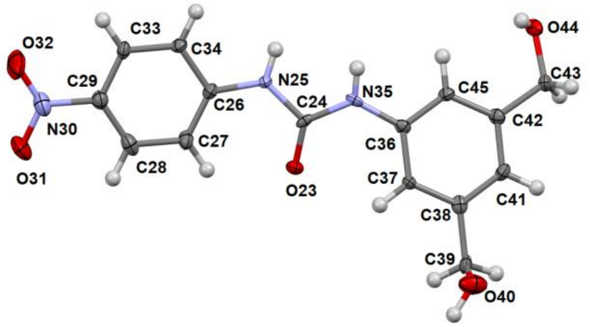

molecule b

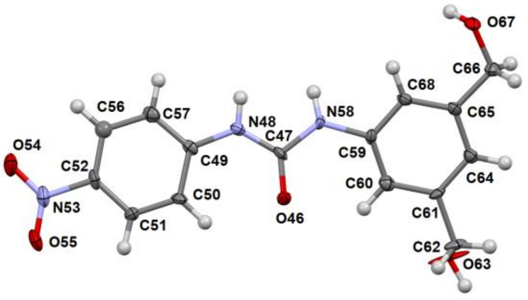

molecule c

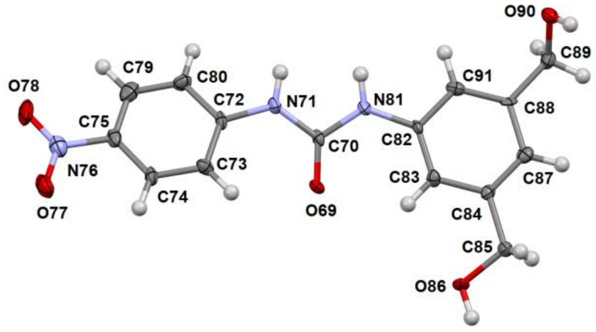

molecule d

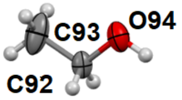

molecule e (ethanol)

Figure S 10 The asymmetric unit of compound **1** obtained from single-crystal X-ray diffraction analysis contains four distinct molecules (**a** - **d**) of the urea and one molecule of ethanol (**e**). Atom labelling schemes are shown for each of the molecules. Thermal ellipsoids drawn at 50% probability.

Table S 1 Crystallographic details for Compound **1**

Formula	$(C_{15}H_{15}N_3O_5)_4$. C_2H_5OH
M_r	1315.27
Crystal system	monoclinic
Space group	$P\ 2_1$
Z	2
a /Å	13.64163(1)
b/Å	8.06633(1)
c /Å	27.33949(1)
β /°	92.9696(11)
V / Å^3	3004.340(4)
D_{calc} / g cm^{-3}	1.501
Crystal habit	Colourless block
Crystal dimensions /mm	0.0865 × 0.186 × 0.228
Radiation	Cu K_α (1.54184 Å)
T /K	100
μ /mm^{-1}	0.937
R(*F*), *Rw*(*F*)	3.64, 4.91
CCDC cif deposition number	CCDC 2091253

Table S 2 Selected bond lengths (Å) and angles (°) for Molecules **a - e** in Compound **1**

Molecule a

O(1) – C(2)	1.241(4)	C(2) – N(3) – C(4)	128.8(3)
O(9) – N(8)	1.227(4)	O(9) – N(8) – O(10)	123.7(3)
O(10) – N(8)	1.239(4)	O(9) – N(8) – C(7)	117.8(3)
O(18) – C(17)	1.416(4)	O(10) – N(8) – C(7)	118.5(3)
O(22) – C(21)	1.440(4)	C(2) – N(13) – C(14)	129.9(3)
N(3) – C(2)	1.388(4)	O(1) – C(2) – N(3)	123.0(3)
N(3) – C(4)	1.397(4)	O(1) – C(2) – N(13)	125.4(3)
N(8) – C(7)	1.471(4)	N(3) – C(2) – N(13)	111.6(3)
N(13) – C(2)	1.339(4)	N(3) – C(4) – C(5)	123.8(3)
N(13) – C(14)	1.406(4)	N(3) – C(4) – C(12)	116.4(3)
C(4) – C(5)	1.392(4)	C(5) – C(4) – C(12)	119.8(3)
C(4) – C(12)	1.389(5)	C(4) – C(5) – C(6)	119.0(3)
C(5) – C(6)	1.390(4)	C(5) – C(6) – C(7)	120.1(3)
C(6) – C(7)	1.372(5)	N(8) – C(7) – C(6)	119.7(3)
C(7) – C(11)	1.406(4)	N(8) – C(7) – C(11)	117.8(3)
C(11) – C(12)	1.386(4)	C(6) – C(7) – C(11)	122.5(3)
C(14) – C(15)	1.418(4)	C(7) – C(11) – C(12)	116.3(3)
C(14) – C(23)	1.380(4)	C(4) – C(12) – C(11)	122.3(3)
C(15) – C(16)	1.369(4)	N(13) – C(14) – C(15)	122.9(3)
C(16) – C(17)	1.513(4)	N(13) – C(14) – C(23)	117.0(3)
C(16) – C(19)	1.434(4)	C(15) – C(14) – C(23)	120.0(3)
C(19) – C(20)	1.355(5)	C(14) – C(15) – C(16)	119.3(3)
C(20) – C(21)	1.522(4)	C(15) – C(16) – C(17)	120.9(3)
C(20) – C(23)	1.417(4)	C(15) – C(16) – C(19)	120.4(3)

		C(17) – C(16) – C(19)	118.7(3)
		O(18) – C(17) – C(16)	108.2(2)
		C(16) – C(19) – C(20)	120.0(3)
		C(19) – C(20) – C(21)	121.4(3)
		C(19) – C(20) – C(23)	119.9(3)
		C(21) – C(20) – C(23)	118.8(3)
		O(22) – C(21) – C(20)	112.1(2)
		C(14) – C(23) – C(20)	120.3(3)

Molecule b

O(23) – C(24)	1.220(4)	C(24) – N(25) – C(26)	125.8(3)
O(31) – N(30)	1.251(4)	O(31) – N(30) – O(32)	122.8(3)
O(32) – N(30)	1.225(4)	O(31) – N(30) – C(29)	118.1(3)
O(40) – C(39)	1.422(4)	O(32) – N(30) – C(29)	119.0(3)
O(44) – C(43)	1.422(4)	C(24) – N(35) – C(45)	127.1(3)
N(25) – C(24)	1.403(3)	O(23) – C(24) – N(25)	123.5(3)
N(25) – C(26)	1.393(4)	O(23) – C(24) – N(35)	124.3(3)
N(30) – C(29)	1.440(4)	N(25) – C(24) – N(35)	112.2(3)
N(35) – C(24)	1.350(4)	N(25) – C(26) – C(27)	125.3(3)
N(35) – C(36)	1.393(4)	N(25) – C(26) – C(34)	114.5(3)
C(26) – C(27)	1.371(4)	C(27) – C(26) – C(34)	120.2(3)
C(26) – C(34)	1.423(4)	C(26) – C(27) – C(28)	119.6(3)
C(27) – C(28)	1.414(4)	C(27) – C(28) – C(29)	119.9(3)
C(28) – C(29)	1.378(5)	N(30) – C(29) – C(28)	119.6(3)
C(29) – C(33)	1.389(4)	N(30) – C(29) – C(33)	119.1(3)
C(33) – C(34)	1.394(4)	C(28) – C(29) – C(33)	121.2(3)
C(36) – C(37)	1.409(4)	C(29) – C(33) – C(34)	119.3(3)

C(36) – C(45)	1.401(4)	C(26) – C(34) – C(33)	119.7(3)
C(37) – C(38)	1.405(4)	N(35) – C(36) – C(37)	123.9(3)
C(38) – C(39)	1.495(4)	N(35) – C(36) – C(45)	116.9(3)
C(38) – C(41)	1.384(4)	C(37) – C(36) – C(45)	119.2(3)
C(41) – C(42)	1.398(4)	C(36) – C(37) – C(38)	119.4(3)
C(42) – C(43)	1.508(4)	C(37) – C(38) – C(39)	119.5(3)
C(42) – C(45)	1.403(4)	C(37) – C(38) – C(41)	120.6(3)
		C(39) – C(38) – C(41)	119.9(3)
		O(40) – C(39) – C(38)	110.0(3)
		C(38) – C(41) – C(42)	120.8(3)
		C(41) – C(42) – C(43)	118.7(3)
		C(41) – C(42) – C(45)	118.8(3)
		C(43) – C(42) – C(45)	122.4(3)
		O(44) – C(43) – C(42)	114.4(3)
		C(36) – C(45) – C(42)	121.1(3)

Molecule c

O(46) – C(47)	1.227(4)	C(47) – N(48) – C(49)	129.4(3)
O(54) – N(53)	1.217(4)	O(54) – N(53) – O(55)	124.4(3)
O(55) – N(53)	1.241(4)	O(54) – N(53) – C(52)	118.9(3)
O(63) – C(62)	1.382(4)	O(55) – N(53) – C(52)	116.7(3)
O(67) – C(66)	1.424(4)	C(47) – N(58) – C(59)	126.2(3)
N(48) – C(47)	1.340(4)	O(46) – C(47) – N(48)	123.7(3)
N(48) – C(49)	1.396(4)	O(46) – C(47) – N(58)	123.4(3)
N(53) – C(52)	1.477(4)	N(48) – C(47) – N(58)	112.9(3)
N(58) – C(47)	1.389(4)	N(48) – C(49) – C(50)	123.2(3)
N(58) – C(59)	1.440(4)	N(48) – C(49) – C(57)	118.4(3)

C(49) – C(50)	1.425(4)	C(50) – C(49) – C(57)	118.4(3)
C(49) – C(57)	1.384(5)	C(49) – C(50) – C(51)	120.2(3)
C(50) – C(51)	1.358(4)	C(50) – C(51) – C(52)	119.1(3)
C(51) – C(52)	1.384(5)	N(53) – C(52) – C(51)	118.6(3)
C(52) – C(56)	1.385(5)	N(53) – C(52) – C(56)	119.0(3)
C(56) – C(57)	1.369(4)	C(51) – C(52) – C(56)	122.3(3)
C(59) – C(60)	1.374(4)	C(52) – C(56) – C(57)	117.9(3)
C(59) – C(68)	1.397(4)	C(49) – C(57) – C(56)	122.0(3)
C(60) – C(61)	1.393(4)	N(58) – C(59) – C(60)	124.0(3)
C(61) – C(62)	1.514(4)	N(58) – C(59) – C(68)	115.3(3)
C(61) – C(64)	1.389(4)	C(60) – C(59) – C(68)	120.7(3)
C(64) – C(65)	1.393(4)	C(59) – C(60) – C(61)	119.5(3)
C(65) – C(66)	1.517(4)	C(60) – C(61) – C(62)	120.3(3)
C(65) – C(68)	1.379(4)	C(60) – C(61) – C(64)	119.9(3)
		C(62) – C(61) – C(64)	119.9(3)
		O(63) – C(62) – C(61)	111.4(3)
		C(61) – C(64) – C(65)	120.5(3)
		C(64) – C(65) – C(66)	118.2(3)
		C(64) – C(65) – C(68)	119.2(3)
		C(66) – C(65) – C(68)	122.6(3)
		O(67) – C(66) – C(65)	113.8(3)
		C(59) – C(68) – C(65)	120.2(3)

Molecule d

O(69) – C(70)	1.219(4)	C(70) – N(71) – C(72)	127.7(3)
O(77) – N(76)	1.239(4)	O(77) – N(76) – O(78)	122.1(3)
O(78) – N(76)	1.222(4)	O(77) – N(76) – C(75)	119.6(3)

O(86) – C(85)	1.426(4)	O(78) – N(76) – C(75)	118.2(3)
O(90) – C(89)	1.433(4)	C(70) – N(81) – C(82)	127.6(3)
N(71) – C(70)	1.364(4)	O(69) – C(70) – N(71)	124.9(3)
N(71) – C(72)	1.399(4)	O(69) – C(70) – N(81)	124.7(3)
N(76) – C(75)	1.449(4)	N(71) – C(70) – N(81)	110.4(3)
N(81) – C(70)	1.392(3)	N(71) – C(72) – C(73)	124.1(3)
N(81) – C(72)	1.415(4)	N(71) – C(72) – C(80)	115.8(3)
C(72) – C(73)	1.399(4)	C(73) – C(72) – C(80)	119.9(3)
C(72) – C(80)	1.417(5)	C(72) – C(73) – C(74)	119.7(3)
C(73) – C(74)	1.387(4)	C(73) – C(74) – C(75)	119.0(3)
C(74) – C(75)	1.393(5)	N(76) – C(75) – C(74)	118.7(3)
C(75) – C(79)	1.368(5)	N(76) – C(75) – C(79)	119.7(3)
C(79) – C(80)	1.369(5)	C(74) – C(75) – C(79)	121.6(3)
C(82) – C(83)	1.371(4)	C(75) – C(79) – C(80)	120.5(3)
C(82) – C(91)	1.412(4)	C(72) – C(80) – C(79)	119.2(3)
C(83) – C(84)	1.413(4)	N(81) – C(82) – C(83)	124.7(3)
C(84) – C(85)	1.507(4)	N(81) – C(82) – C(91)	115.5(3)
C(84) – C(87)	1.360(4)	C(83) – C(82) – C(91)	119.7(3)
C(87) – C(88)	1.423(4)	C(82) – C(83) – C(84)	120.0(3)
C(88) – C(89)	1.497(4)	C(83) – C(84) – C(85)	119.6(3)
C(88) – C(91)	1.371(4)	C(83) – C(84) – C(87)	120.2(3)
		C(85) – C(84) – C(87)	120.3(3)
		O(86) – C(85) – C(84)	110.4(2)
		C(84) – C(87) – C(88)	120.4(3)
		C(87) – C(88) – C(89)	121.4(3)
		C(87) – C(88) – C(91)	119.1(3)
		C(89) – C(88) – C(91)	119.5(3)

		O(90) – C(89) – C(88)	112.1(2)
		C(82) – C(91) – C(88)	120.7(3)

Ethanol Molecule e

O(94) – C(93)	1.322(4)	O(94) – C(93) – C(22)	111.4(3)
C(92) – C(93)	1.515(3)		

Table S 3 Hydrogen-bond interactions (Å, °) for compound **1**.

D – H•••A	*D* – H	H•••*A*	*D* •••*A*	*D* – H•••*A*
N(3) – H(31)•••O(90)^{i}	0.85	2.06	2.877(5)	160
N(13) – H(131)•••O(90)^{i}	0.85	2.10	2.912(5)	160
N(25) – H(251)•••O(44)^{ii}	0.85	2.01	2.832(5)	162
N(35) – H(351)•••O(44)^{ii}	0.85	2.01	2.832(5)	162
N(48) – H(481)•••O(67)^{iii}	0.85	2.03	2.859(5)	163
N(58) – H(581)•••O(67)^{iii}	0.85	2.28	3.048(5)	151
N(71) – H(711)•••O(22)^{iv}	0.85	2.06	2.877(5)	160
N(81) – H(811)•••O(22)^{iv}	0.85	2.08	2.882(5)	156
O(18) – H(181)•••O(1)^{iii}	0.892	1.874	2.762(5)	173(3)
O(22) – H(221)•••O(40)^{v}	0.890	1.901	2.698(5)	148(3)
O(40) – H(401)•••O(46)^{vi}	0.89	1.92	2.671(5)	141
O(44) – H(441)•••O(86)^{vii}	0.891	1.836	2.706(5)	165(3)
O(63) – H(631)•••O(23)^{viii}	0.892	1.815	2.645(5)	154(3)
O(67) – H(671)•••O(18)^{ix}	0.89(2)	1.824	2.703(5)	171(3)
O(86) – H(861)•••O(69)^{x}	0.888	1.978	2.759(5)	146(2)
O(90) – H(901)•••O(63)^{iv}	0.90(2)	1.754	2.648(5)	171(3)
O(94) – H(941)•••O(63)^{vi}	0.90	2.33	3.226(5)	177.0

Symmetry Codes: (i) *x*, *y* – 1, *z*; (ii) – *x* – 1, 1/2+*y*, 1 – *z*; (iii) 1 – *x*, *y* – 1/2, – *z*;
(iv) *x*, 1+*y*, *z*; (v) *x* + 1, *y* – 1, *z*; (vi) *x* – 1, *y*, *z*; (vii) – *x*, *y* – 1/2, 1 – *z*;
(viii) *x* + 1, *y*, *z*; (ix) 1 – *x*, 1/2+*y*, – *z*; (x) 1 – *x*, 1/2+*y*, 1 – *z*.

Table S 4 Intermolecular short contacts (Å) for Compound **1**.

C(2) – C(59)	3.335(4)
C(19) – C(49)	3.341(4)
C(19) – C(57)	3.389(4)
C(23) – C(47)	3.392(4)
C(33) – C(74)	3.327(4)

Compound 2:

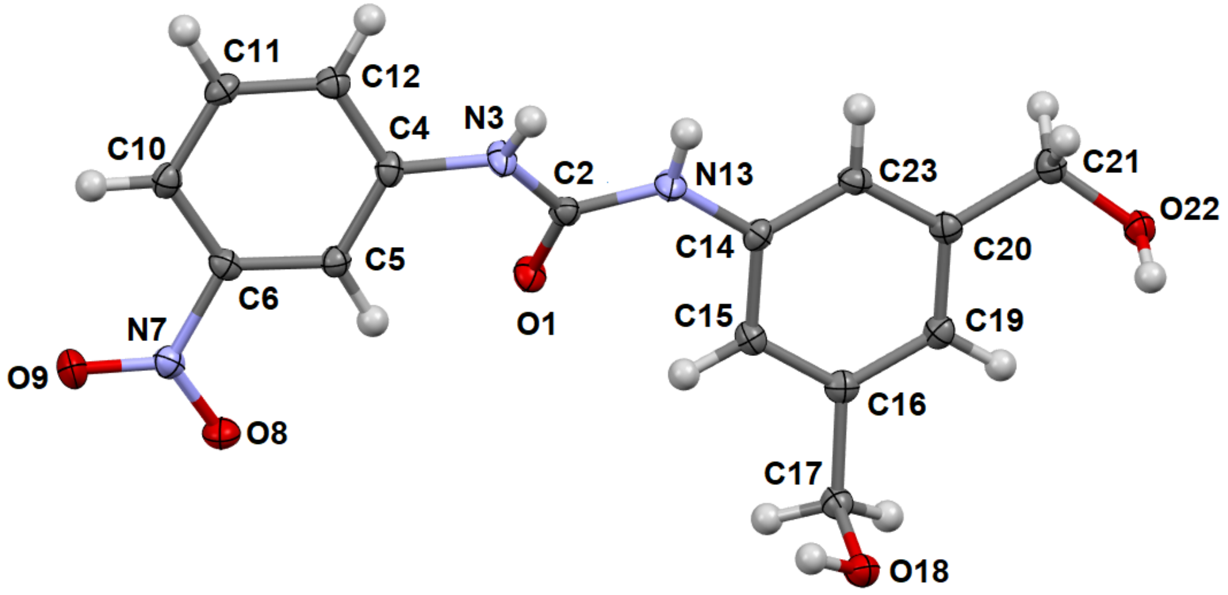

Figure S 11 Asymmetric unit of Compound **2** containing one molecule of the urea obtained from single-crystal X-ray diffraction analysis showing the atom labelling scheme. Thermal ellipsoids drawn at 50% probability.

Table S 5 Crystallographic details for Compound **2**

Formula	$C_{15} H_{15} N_3 O_5$
M_r	317.30
Crystal system	orthorhombic
Space group	$P\ 2_1\ 2_1\ 2_1$
Z	4
a /Å	4.739410(9)
b/Å	7.106140(10)
c /Å	41.69148(3)
V / Å^3	1404.123(3)
D_{calc} / g cm^{-3}	1.501
Crystal habit	Colourless rod
Crystal dimensions /mm	0.025 × 0.026 × 0.071
Radiation	Cu K_α (1.54184 Å)
T /K	100
μ /mm^{-1}	0.968
R(*F*), *Rw*(*F*)	2.92, 3.79
CCDC cif deposition number	CCDC 2254715

Table S 6 Selected bond lengths (Å) and angles (°) for Compound **2**

O(1) – C(2)	1.2256(19)	C(2) – N(3) – C(4)	125.37(13)
O(8) – N(7)	1.2310(17)	O(8) – N(7) – O(9)	123.39(13)
O(9) – N(7)	1.2297(17)	O(8) – N(7) – C(6)	118.02(12)
O(18) – C(17)	1.4415(19)	O(9) – N(7) – C(6)	118.58(12)
O(22) – C(21)	1.4233(18)	C(2) – N(13) – C(14)	126.75(14)
N(3) – C(2)	1.373(2)	O(1) – C(2) – N(3)	123.08(13)
N(3) – C(4)	1.4069(19)	O(1) – C(2) – N(13)	124.05(14)
N(7) – C(6)	1.4674(19)	N(3) – C(2) – N(13)	112.86(13)
N(13) – C(2)	1.359(2)	N(3) – C(4) – C(5)	122.74(13)
N(13) – C(14)	1.420(2)	N(3) – C(4) – C(12)	117.53(13)
C(4) – C(5)	1.388(2)	C(5) – C(4) – C(12)	119.70(14)
C(4) – C(12)	1.403(2)	C(4) – C(5) – C(6)	117.98(14)
C(5) – C(6)	1.391(2)	N(7) – C(6) – C(5)	117.38(13)
C(6) – C(10)	1.383(2)	N(7) – C(6) – C(10)	118.97(13)
C(10) – C(11)	1.385(2)	C(5) – C(6) – C(10)	123.65(14)
C(11) – C(12)	1.392(2)	C(6) – C(10) – C(11)	117.53(14)
C(14) – C(15)	1.393(2)	C(10) – C(11) – C(12)	120.72(14)
C(14) – C(23)	1.394(2)	C(4) – C(12) – C(11)	120.41(14)
C(15) – C(16)	1.390(2)	N(13) – C(14) – C(15)	122.13(14)
C(16) – C(17)	1.505(2)	N(13) – C(14) – C(23)	117.56(14)
C(16) – C(19)	1.393(2)	C(15) – C(14) – C(23)	120.16(14)
C(19) – C(20)	1.392(2)	C(14) – C(15) – C(16)	119.49(13)
C(20) – C(21)	1.515(2)	C(15) – C(16) – C(17)	119.09(13)
C(20) – C(23)	1.394(2)	C(15) – C(16) – C(19)	120.34(14)
		C(17) – C(16) – C(19)	120.33(14)
		O(18) – C(17) – C(16)	110.22(13)

		C(16) – C(19) – C(20)	120.31(14)
		C(19) – C(20) – C(21)	121.66(13)
		C(19) – C(20) – C(23)	119.34(14)
		C(21) – C(20) – C(23)	118.99(14)
		C(14) – C(23) – C(20)	120.33(14)
		O(22) – C(21) – C(20)	113.42(13)

Table S 7 Hydrogen-bond interactions (Å, °) for Compound **2**.

D – H•••A	*D* – H	H•••*A*	*D* •••*A*	*D* – H•••*A*
N(3) – H(31)•••O(1)^{i}	0.86(2)	2.08(3)	2.836(2)	148(2)
N(13) – H(131)•••O(8)^{ii}	0.82(2)	2.22(2)	2.992 (2)	159.0(19)
O(18) – H(181)•••O(22)^{iii}	0.92(2)	1.91(2)	2.813(2)	169.0(19)
O(22) – H(221)•••O(18)^{iv}	0.91(2)	1.82(2)	2.727(2)	172.8(19)

Symmetry Codes: (i) 1+*x*, *y*, *z*; (ii) 1+*x*, 1+*y*, *z*; (iii) *x*, *y* – 1, *z*; (iv) 2– *x*, 1/2+*y*, 1/2 – *z*.

Table S 8 Intermolecular short contacts (Å) for Compound **2**.

C(16) – C(23)^{i}	3.360(2)

(i)

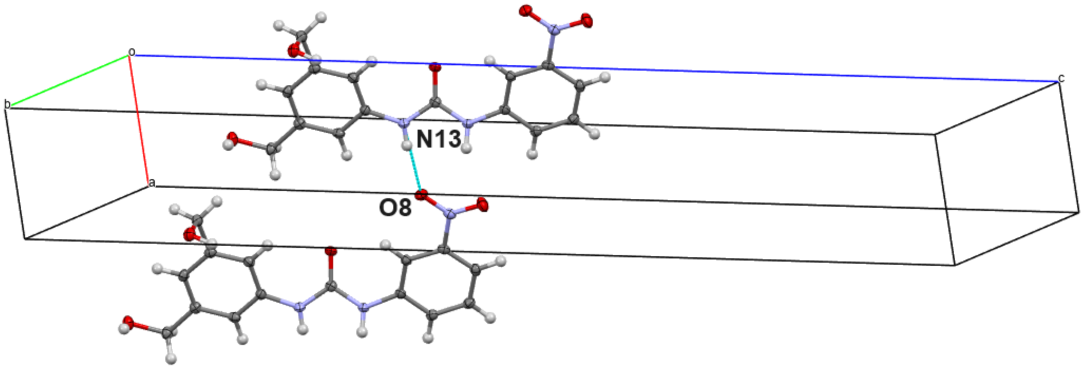

(ii)

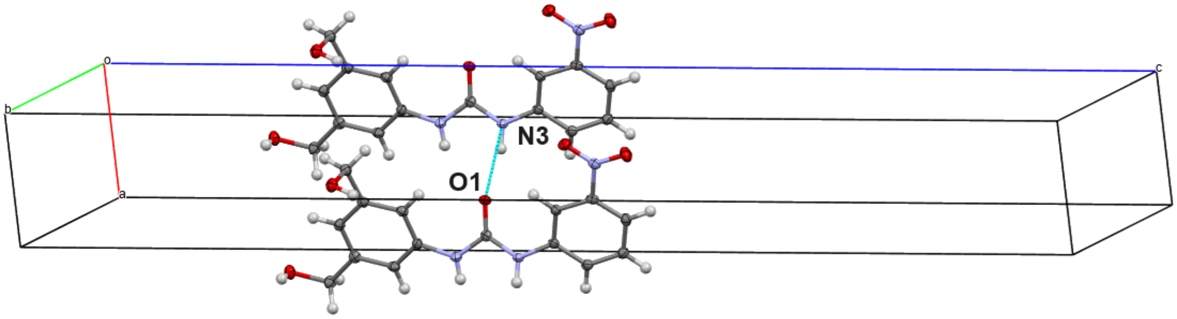

(iii)

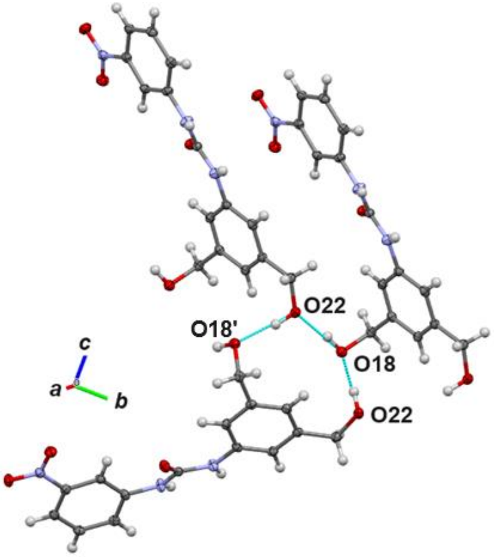

Figure S 12 Packing diagrams for Compound **2**: (i) Hydrogen bonding of a nitro moiety acting as an acceptor for the urea NH of an adjacent molecule (N(13)…O(8), 2.992(2) Å). (ii) off-set hydrogen bonding between two urea moieties (N(3)…O(1), 2.836(2) Å) and (iii) hydrogen bonding between hydroxyl groups in adjacent molecules (O(18)…O(22), 2.813(2) Å) and O(18)'…O(22), 2.727(2) Å).

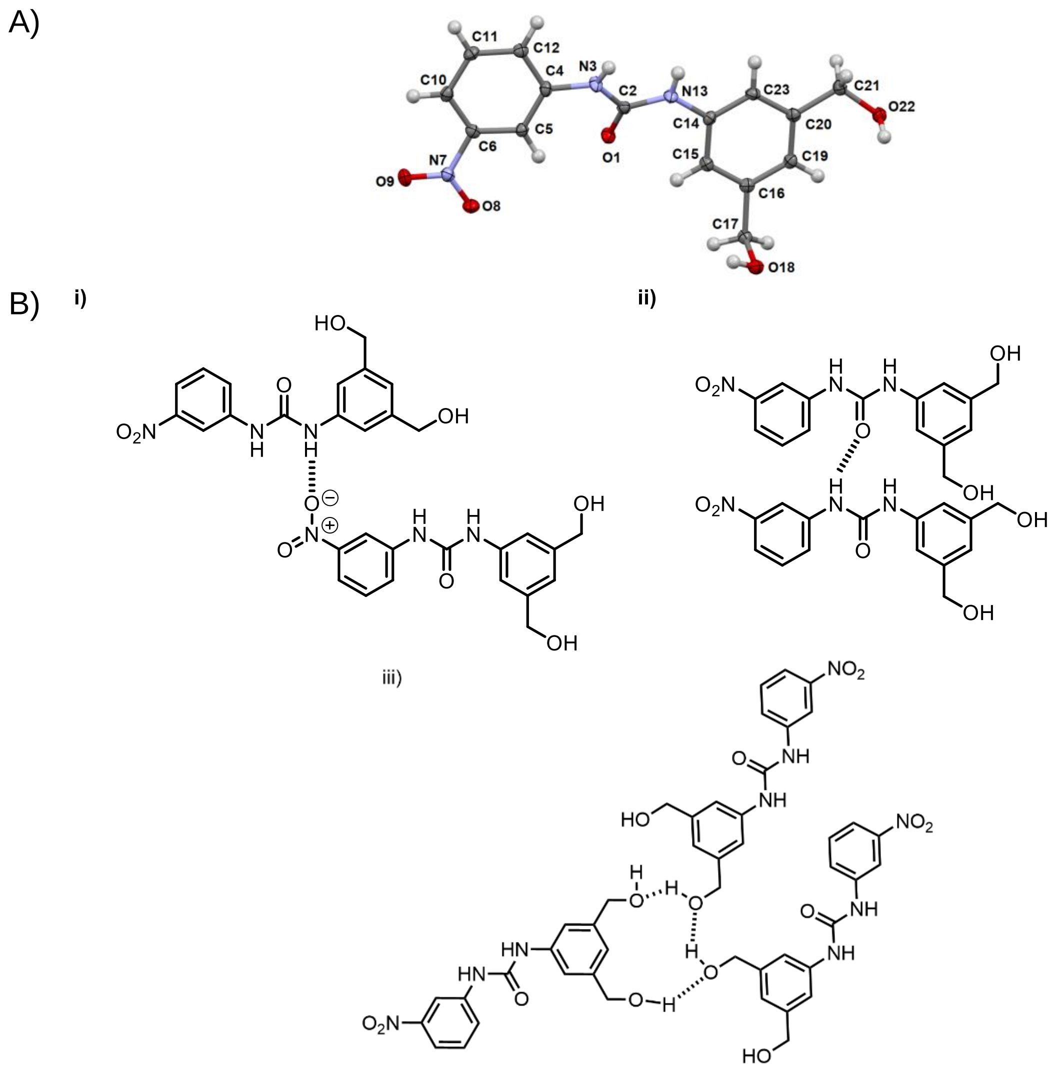

Figure S 13 A) The asymmetric unit of **2** with ellipsoids drawn at 50% probability; B) Schematic representation of the hydrogen-bonding motifs observed within the crystal structure of compound **2**. i) hydrogen bonding of a nitro moiety acting as an acceptor for the urea NH of an adjacent molecule. ii) off-set hydrogen bonding between two urea moieties. iii) hydrogen bonding between O-H groups in adjacent molecules.

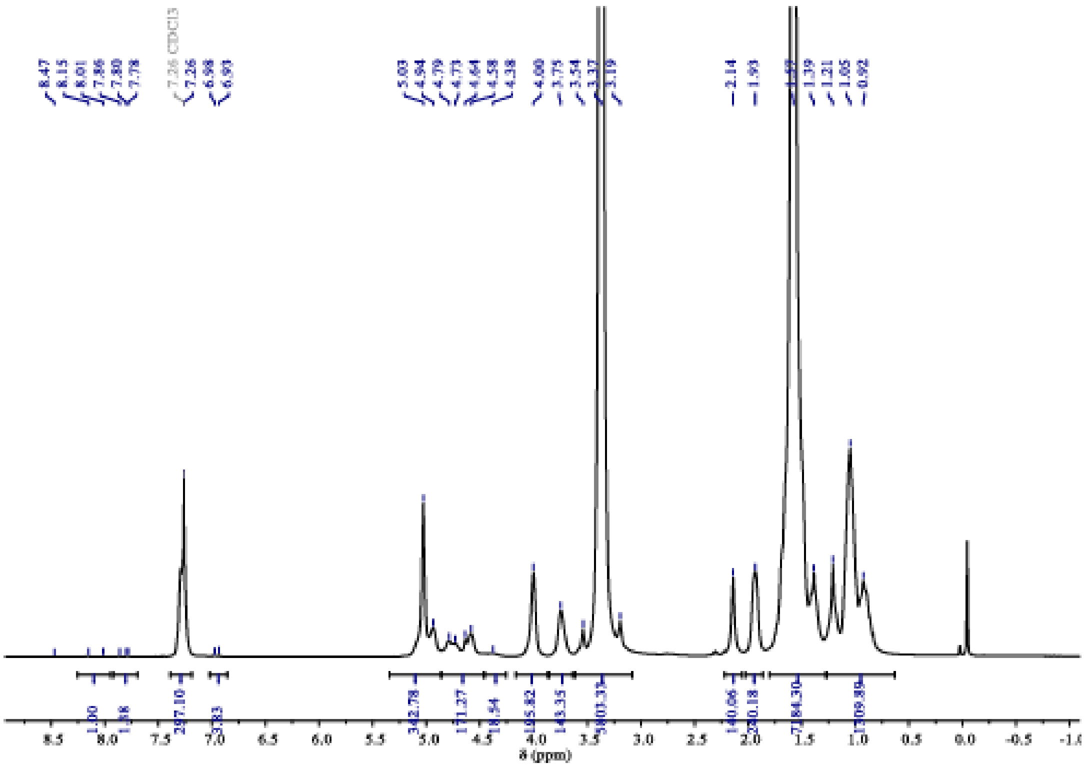

Figure S 14 ^{1}H NMR spectrum of **SPE1** (400 MHz, $CDCl_3$, 298 K).

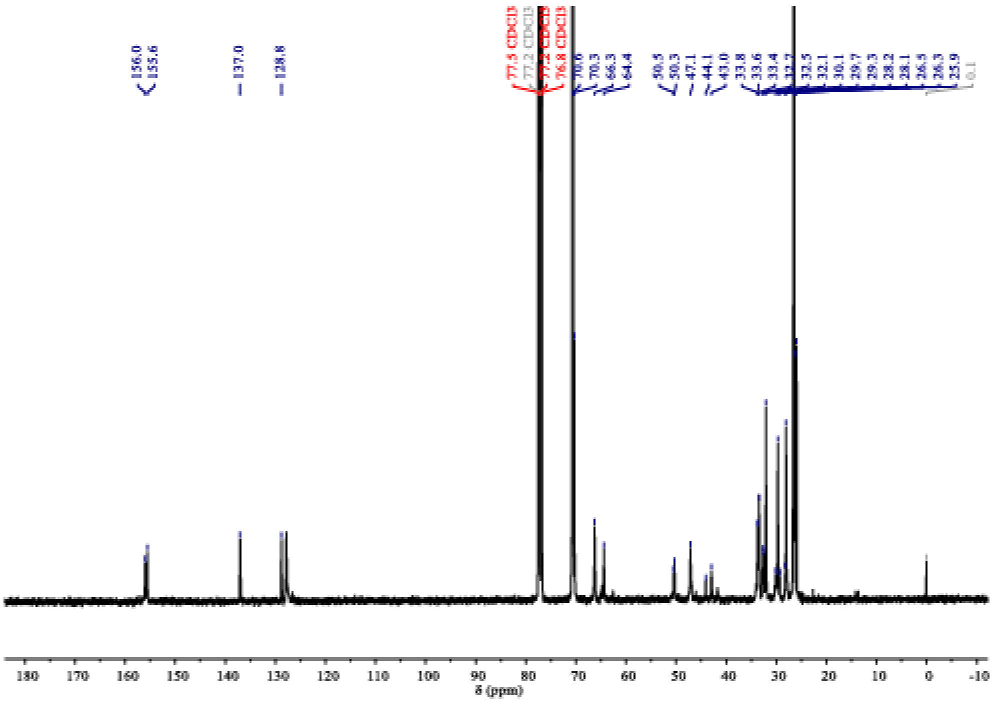

Figure S 15 $^{13}C\{H\}$ NMR spectrum of **SPE1** (100 MHz, $CDCl_3$, 298 K).

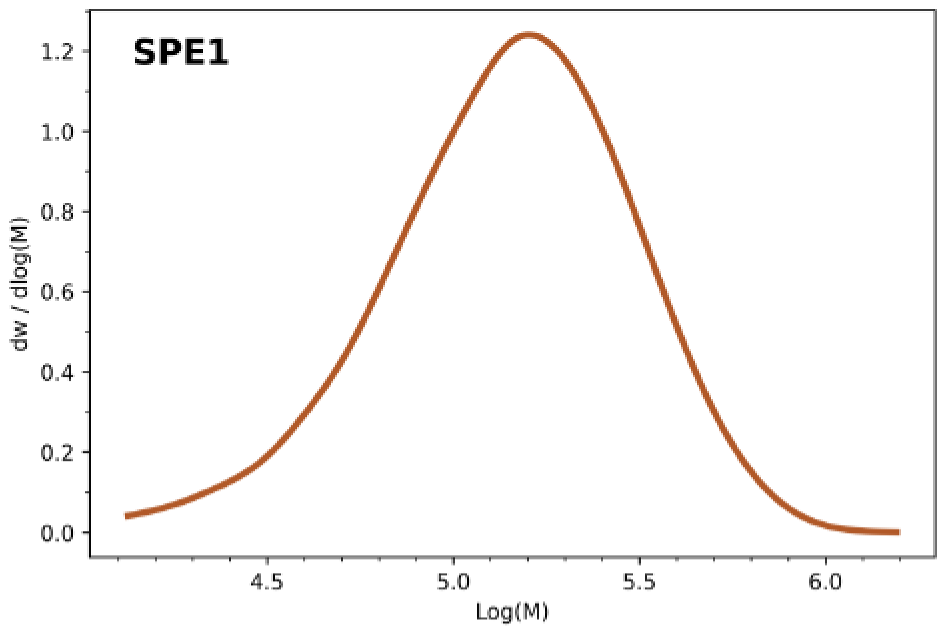

Figure S 16 GPC eluogram of **SPE1** (DMF, room temperature).

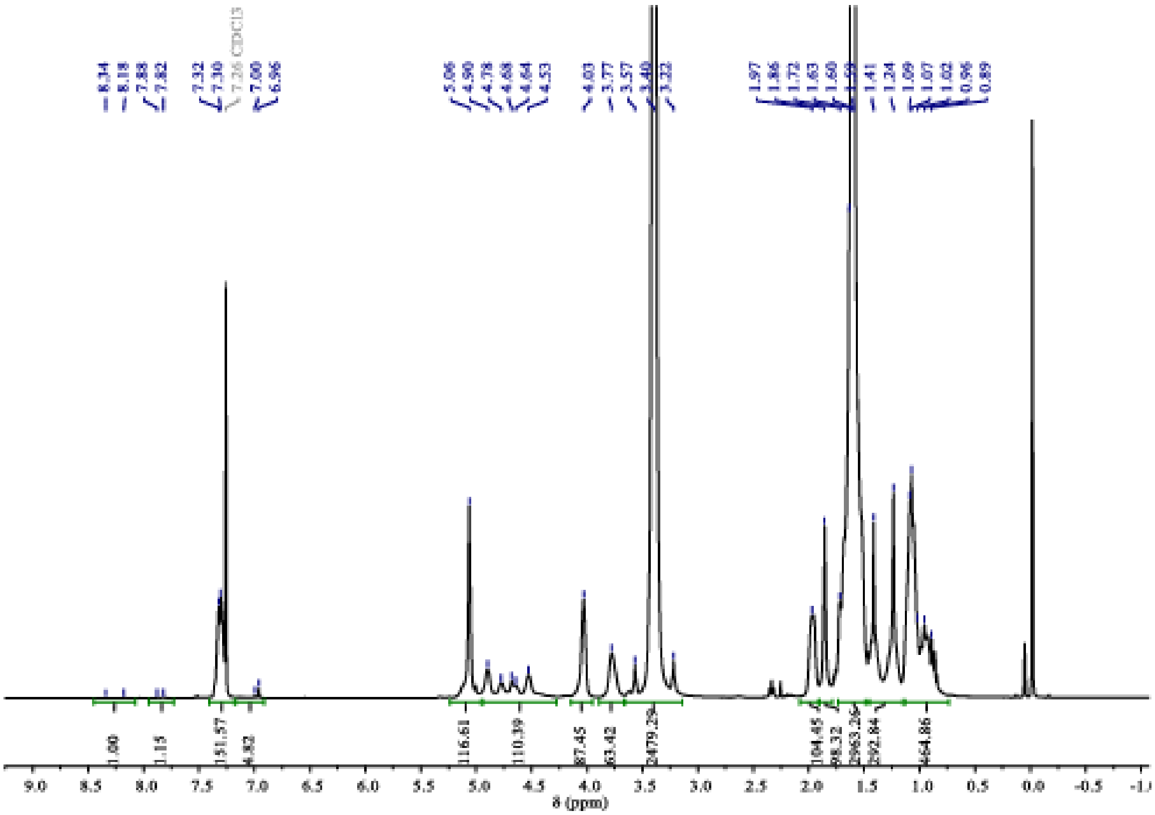

Figure S 17 ^{1}H NMR spectrum of **SPE2** (400 MHz, $CDCl_3$, 298 K).

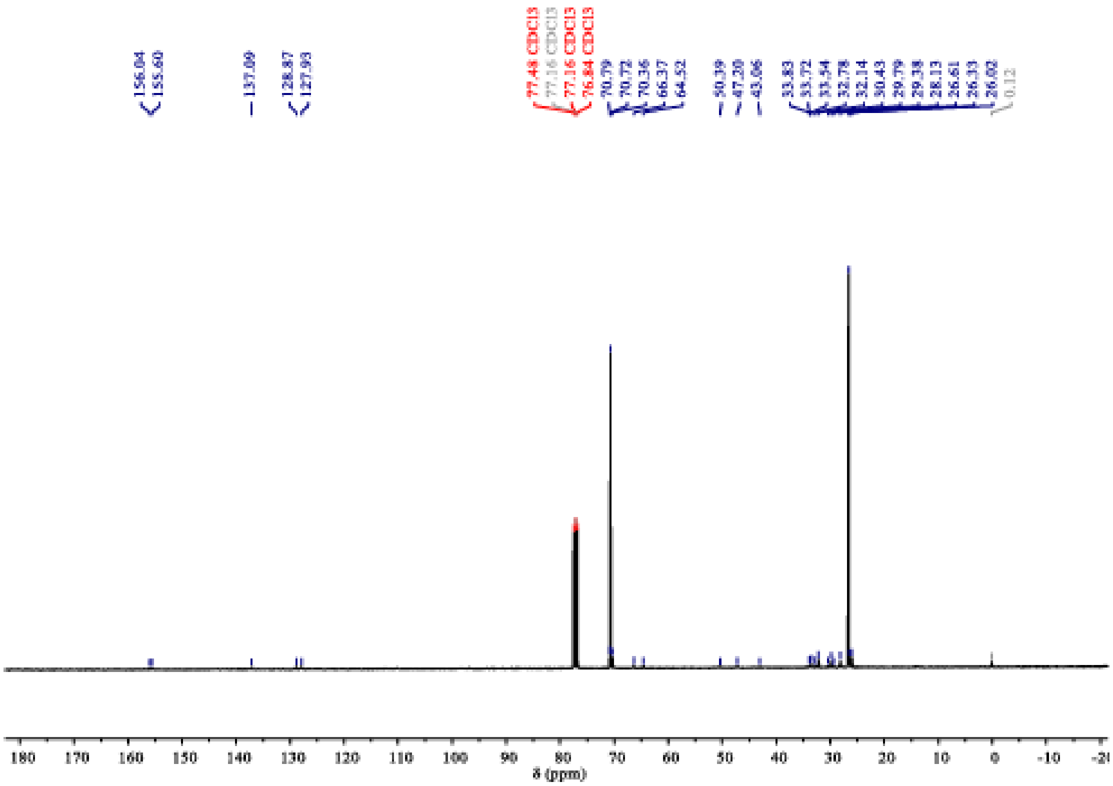

Figure S 18 $^{13}C\{H\}$ NMR spectrum of **SPE2** (100 MHz, $CDCl_3$, 298 K).

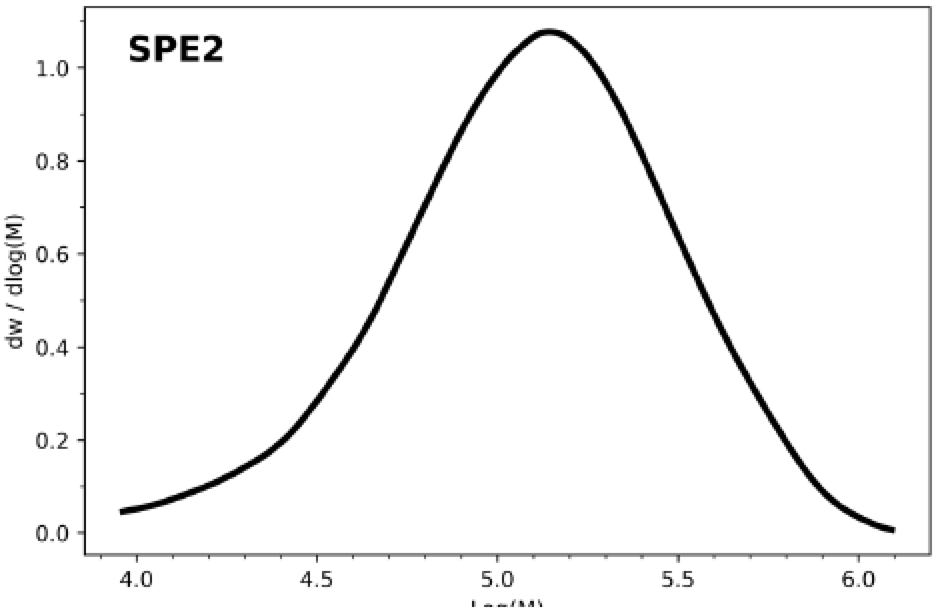

Figure S 19 GPC eluogram of **SPE2** (DMF, room temperature).

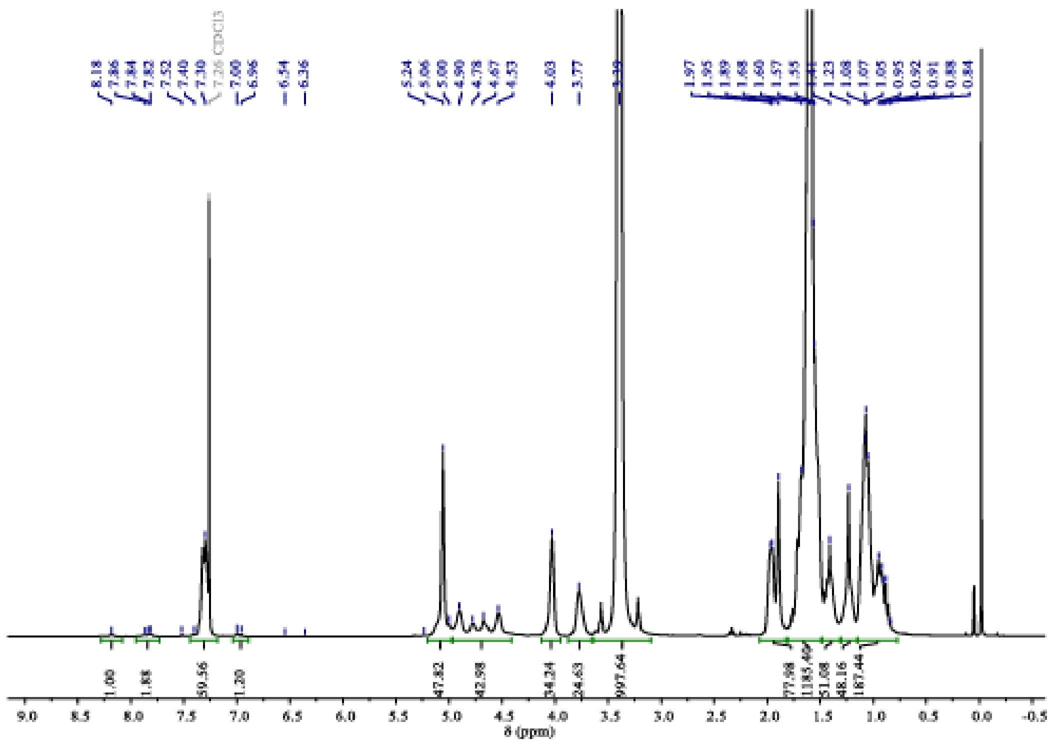

Figure S 20 ^{1}H NMR spectrum of **SPE3** (400 MHz, $CDCl_3$, 298 K).

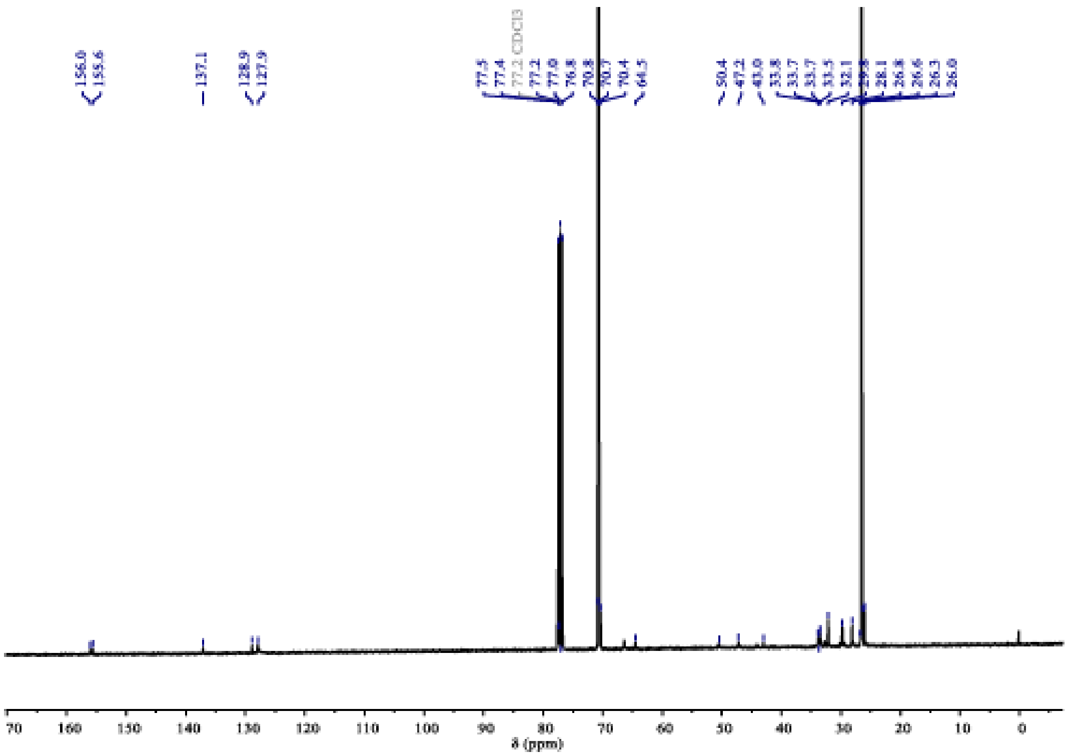

Figure S 21 ^{13}C{H} NMR spectrum of **SPE3** (100 MHz, $CDCl_3$, 298 K).

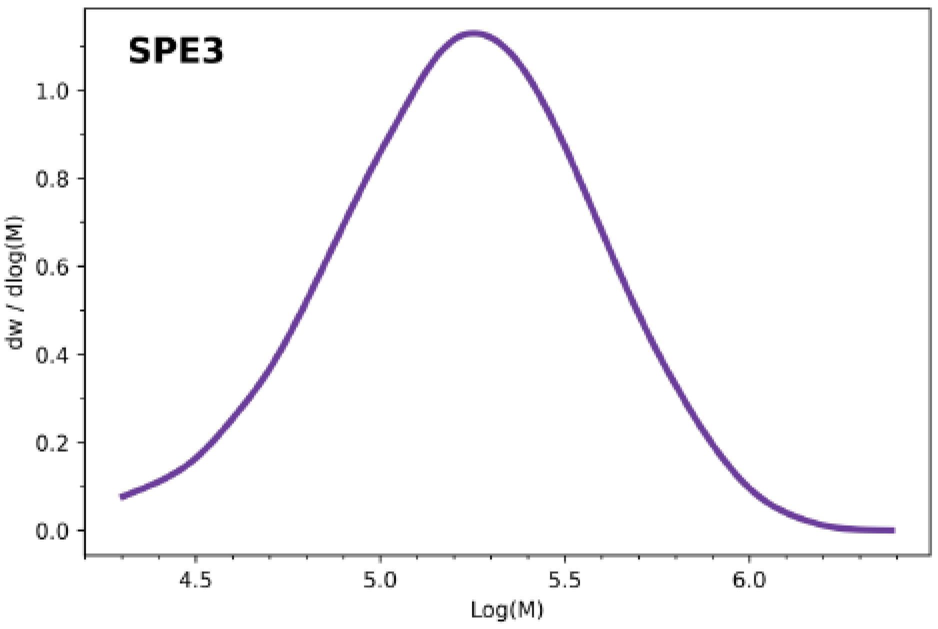

Figure S 22 GPC eluogram of **SPE3** (DMF, room temperature).

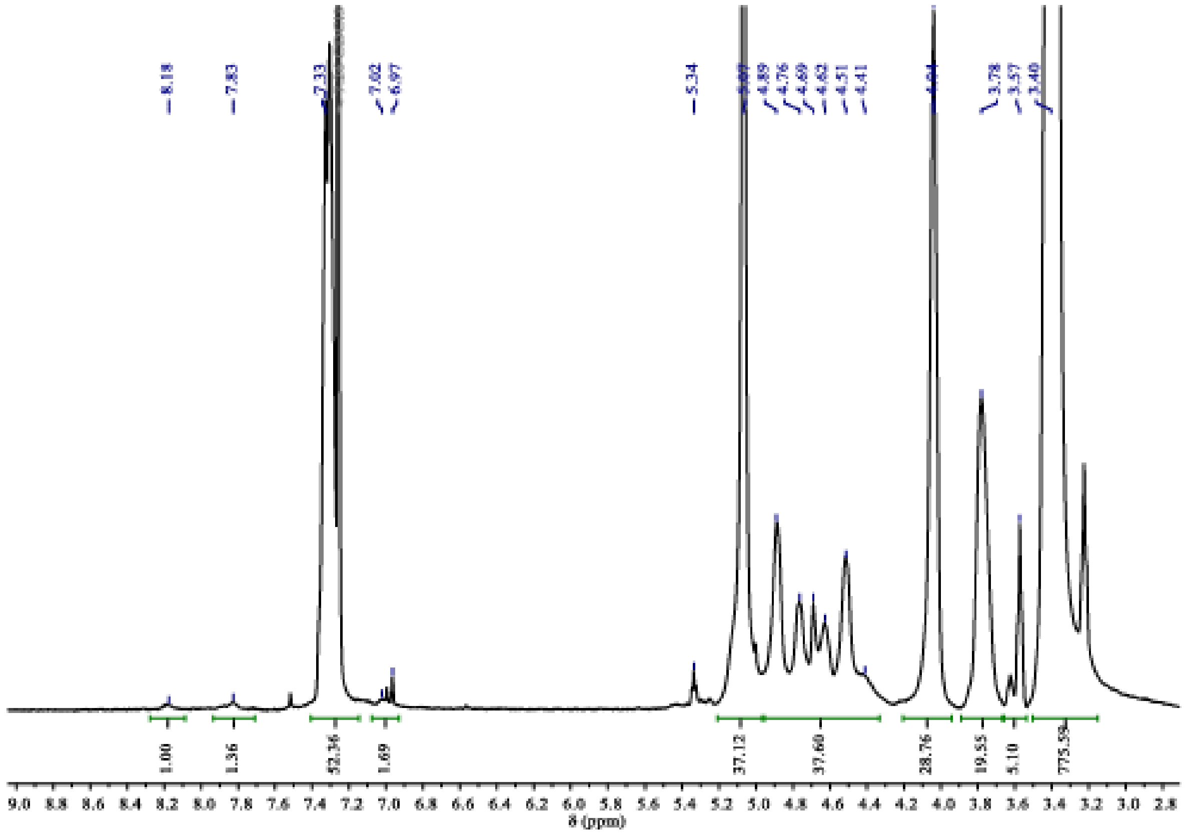

Figure S 23 ^{1}H NMR spectrum of **SPE4** (400 MHz, $CDCl_3$, 298 K).

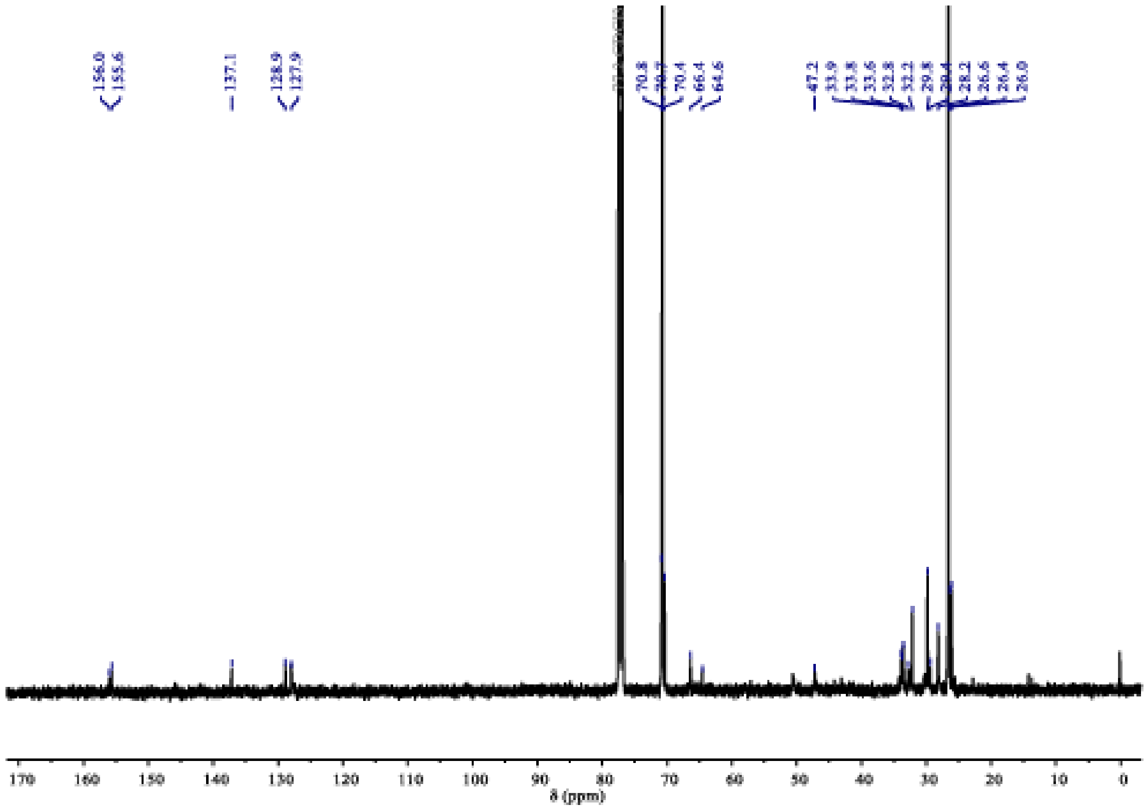

Figure S 24 $^{13}C\{H\}$ NMR spectrum of **SPE4** (100 MHz, $CDCl_3$, 298 K).

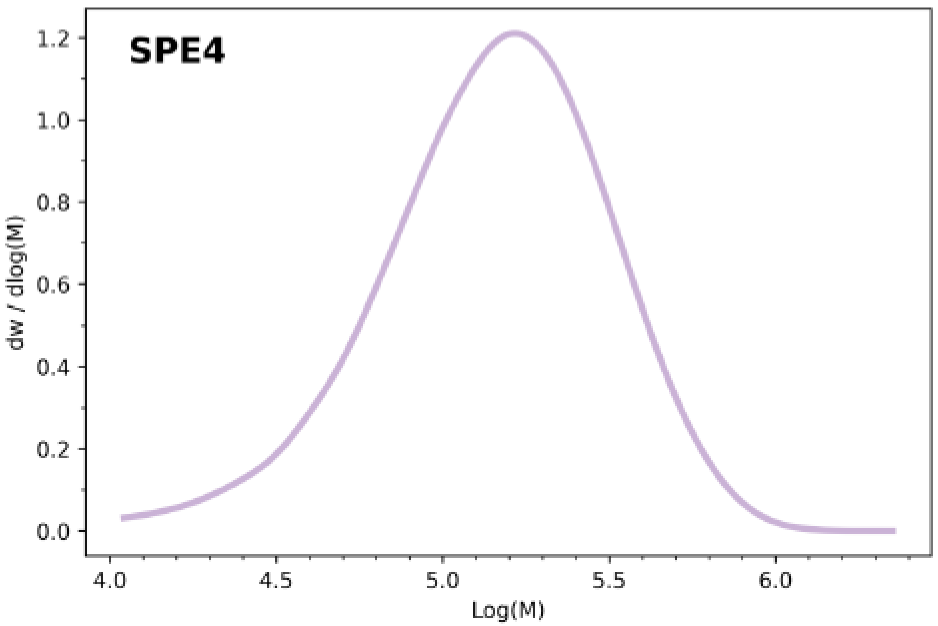

Figure S 25 GPC eluogram of **SPE4** (DMF, room temperature).

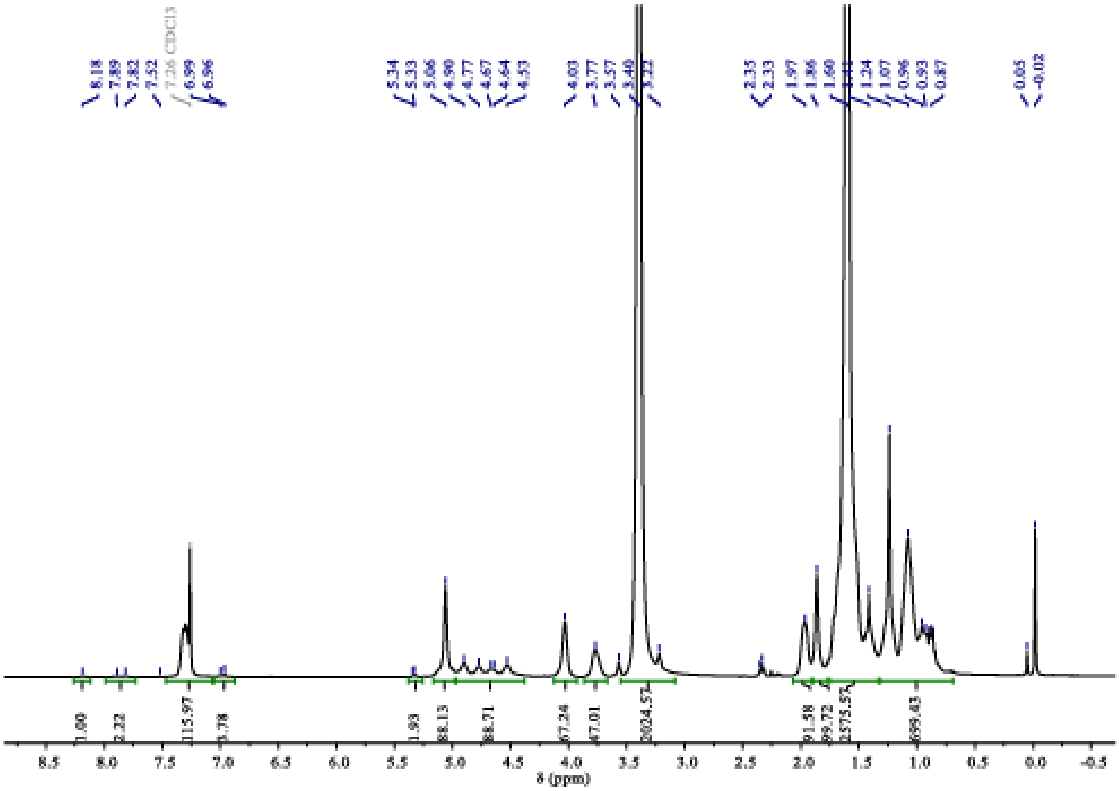

Figure S 26 ^{1}H NMR spectrum of **SPE5** (400 MHz, $CDCl_3$, 298 K)

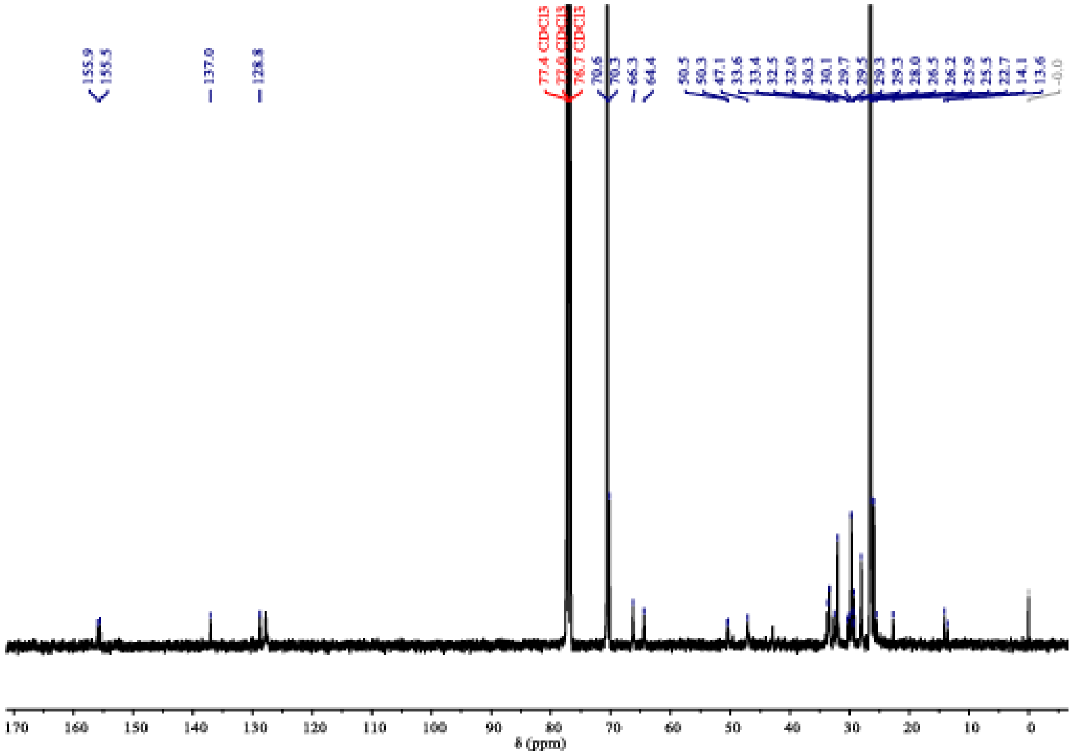

Figure S 27 ^{13}C{H} NMR spectrum of **SPE5** (100 MHz, $CDCl_3$, 298 K).

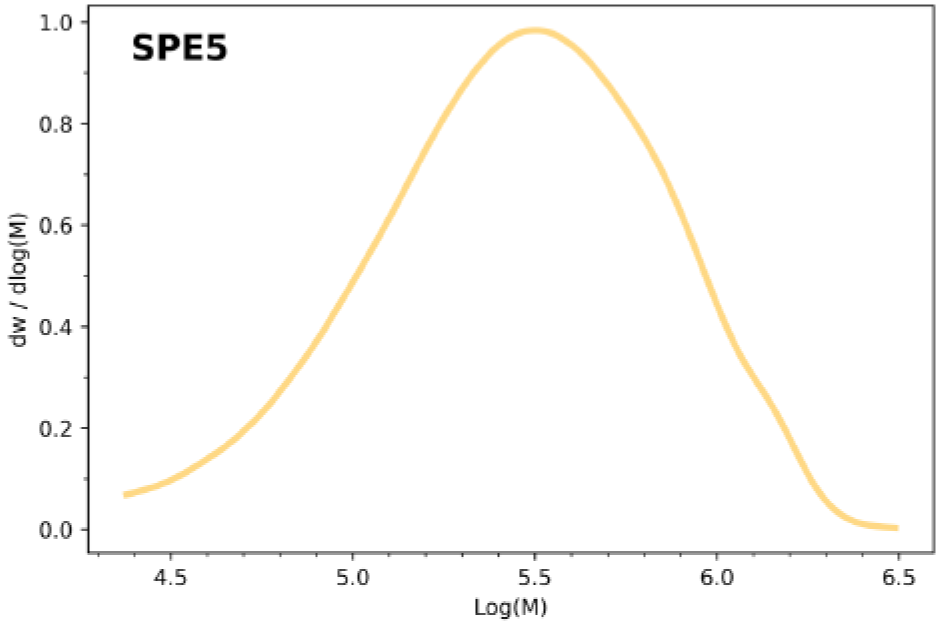

Figure S 28 GPC eluogram of **SPE5** (DMF, room temperature).

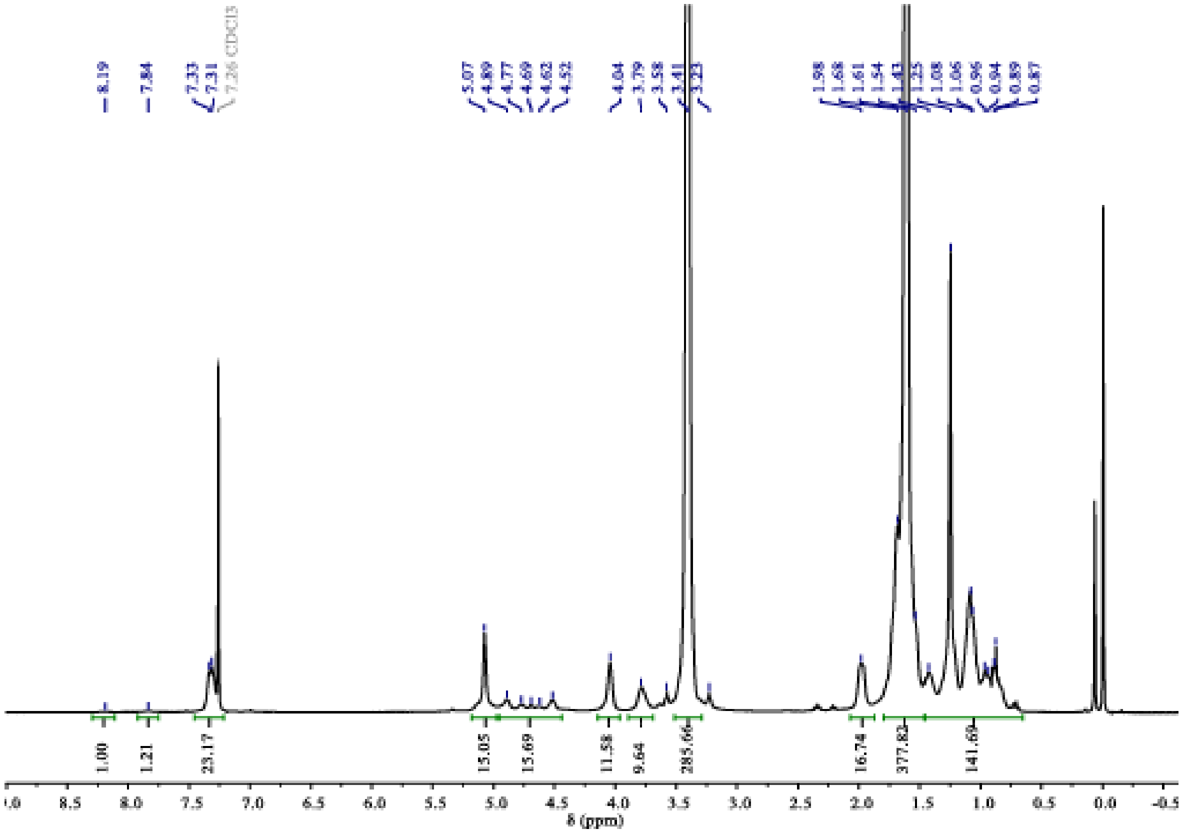

Figure S 29 ^{1}H NMR spectrum of **SPE6** (400 MHz, $CDCl_3$, 298 K).

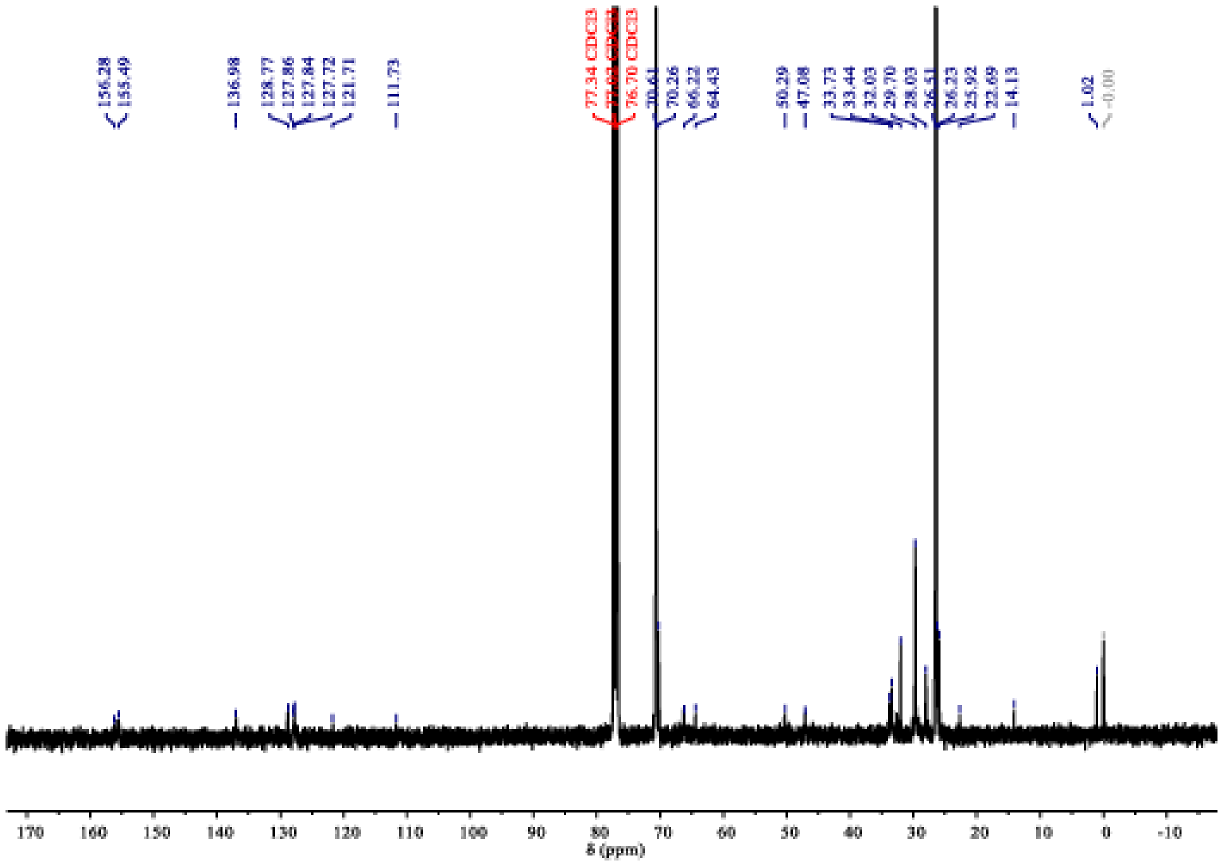

Figure S 30 $^{13}C\{H\}$ NMR spectrum of **SPE6** (100 MHz, $CDCl_3$, 298 K).

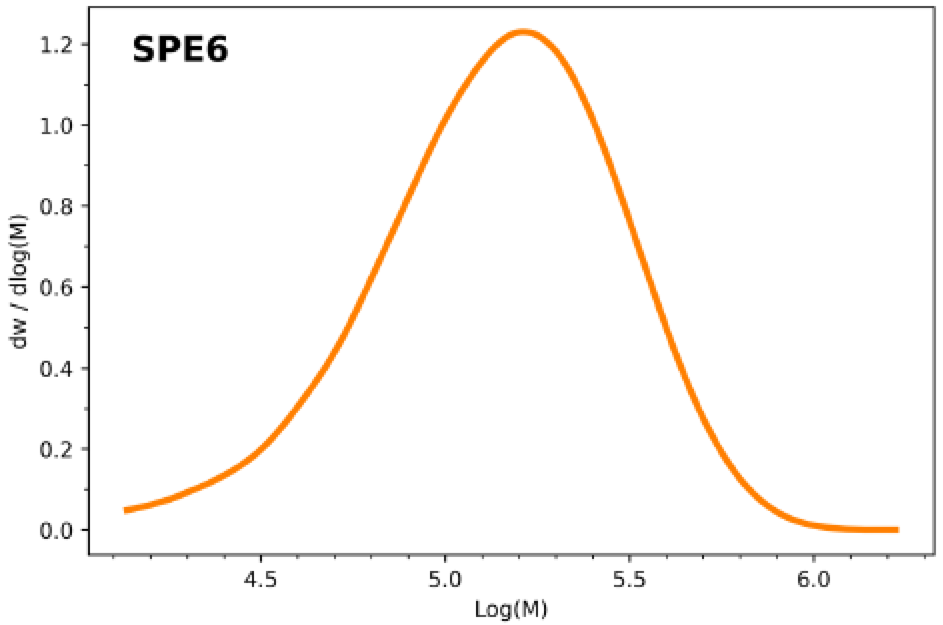

Figure S 31 GPC eluogram of **SPE6** (DMF, room temperature).

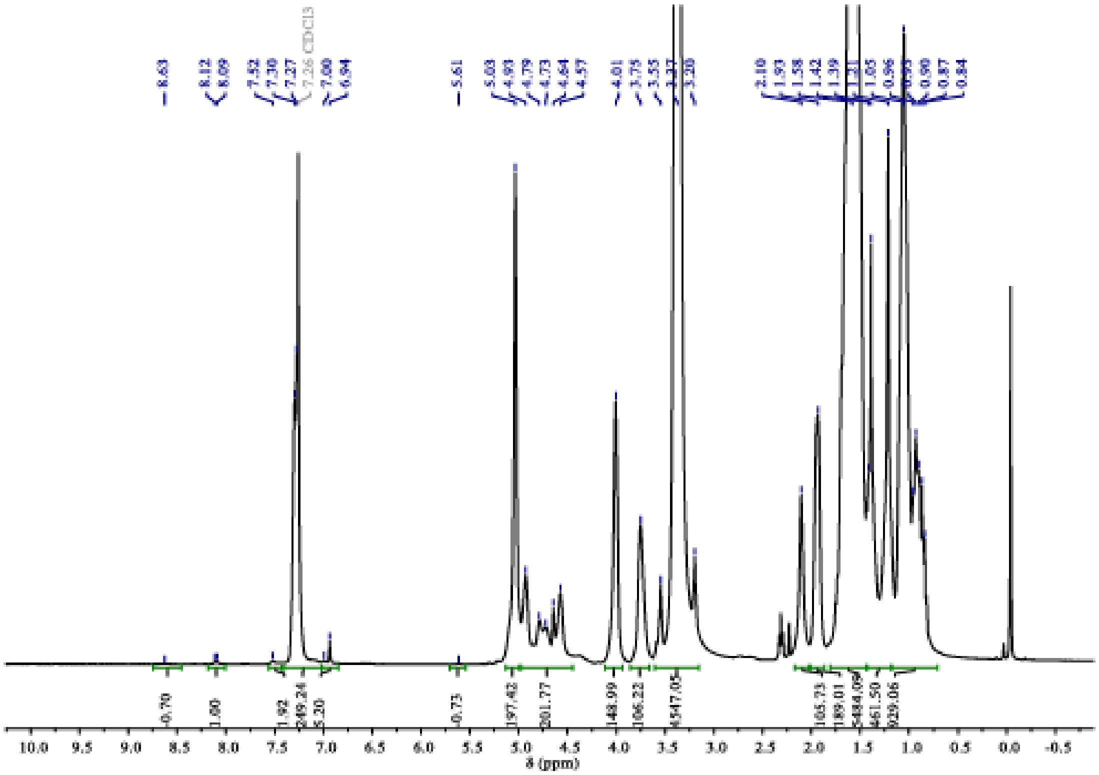

Figure S 32 ^{1}H NMR spectrum of **SPE7** (400 MHz, $CDCl_3$, 298 K).

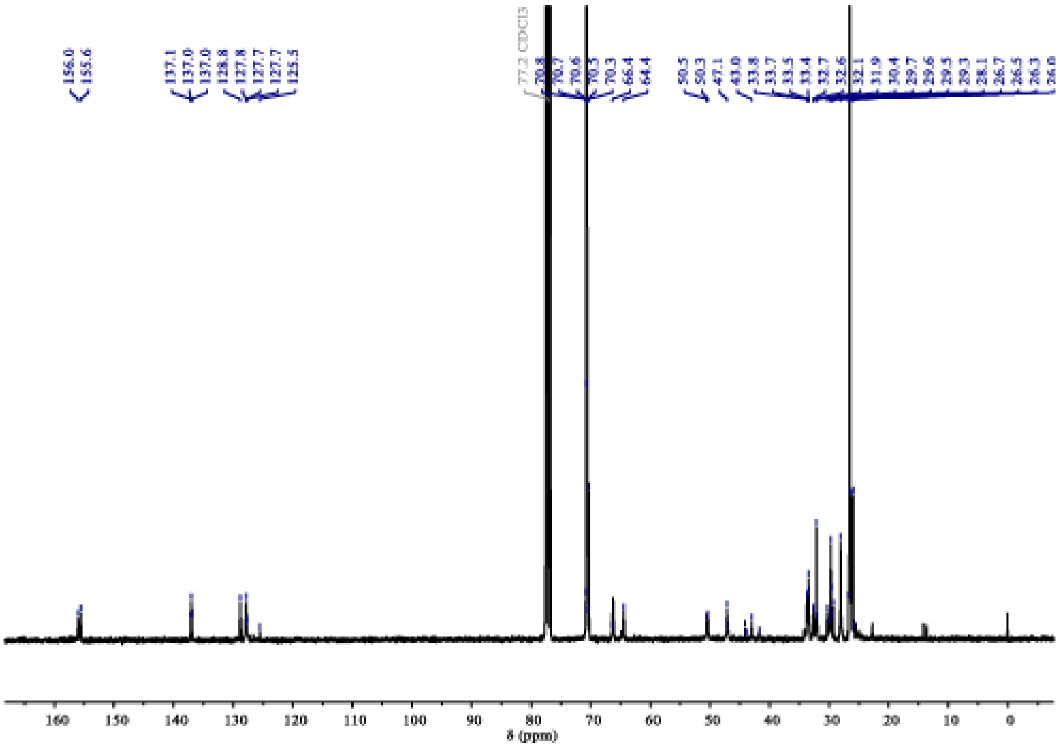

Figure S 33 ^{13}C{H} NMR spectrum of **SPE7** (100 MHz, $CDCl_3$, 298 K).

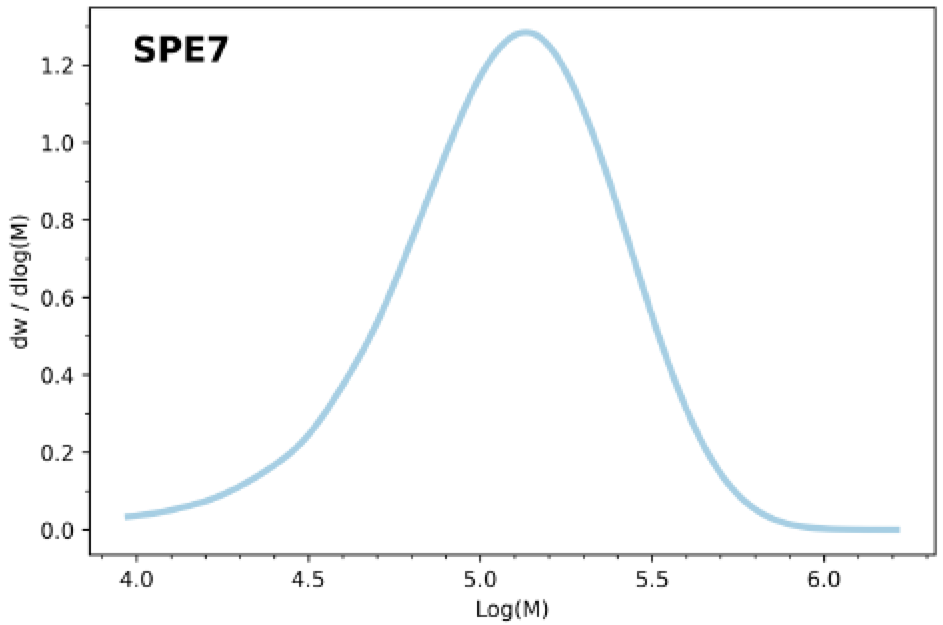

Figure S 34 GPC eluogram of **SPE7** (DMF, room temperature).

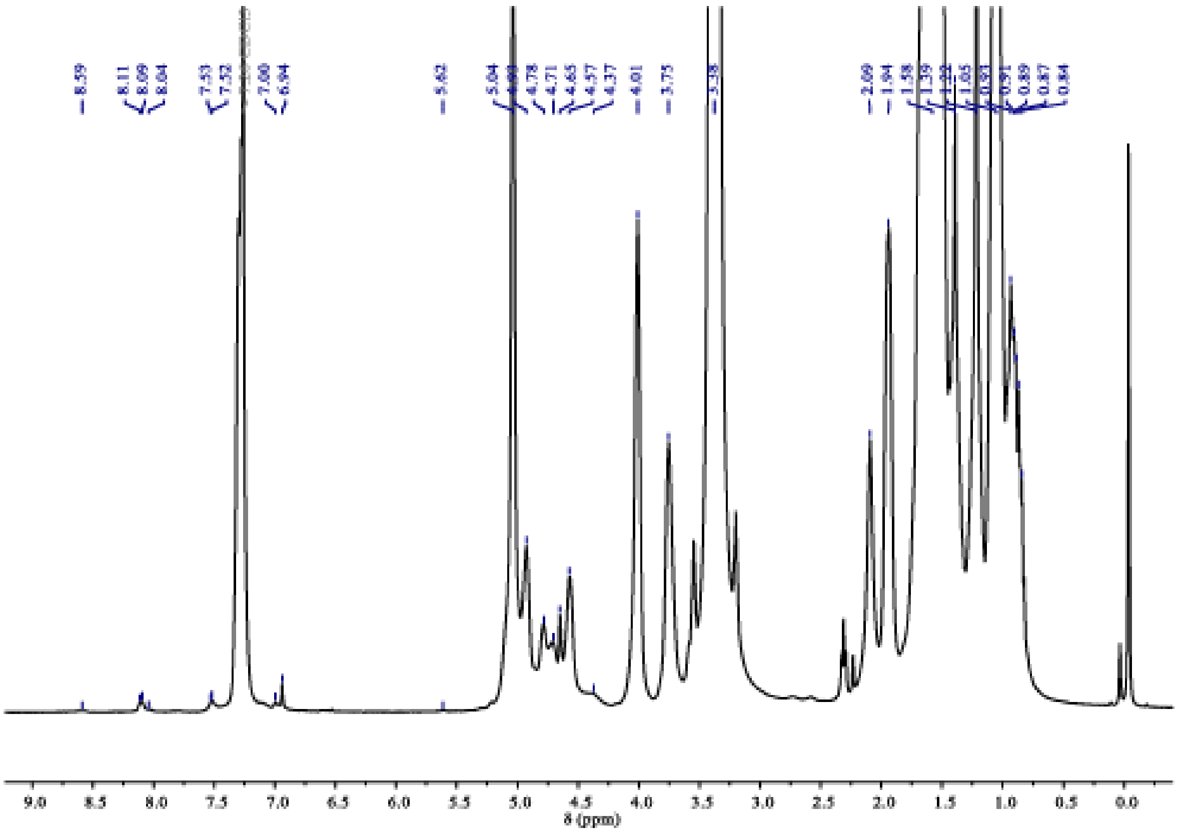

Figure S 35 ^{1}H NMR spectrum of **SPE8** (400 MHz, $CDCl_3$, 298 K).

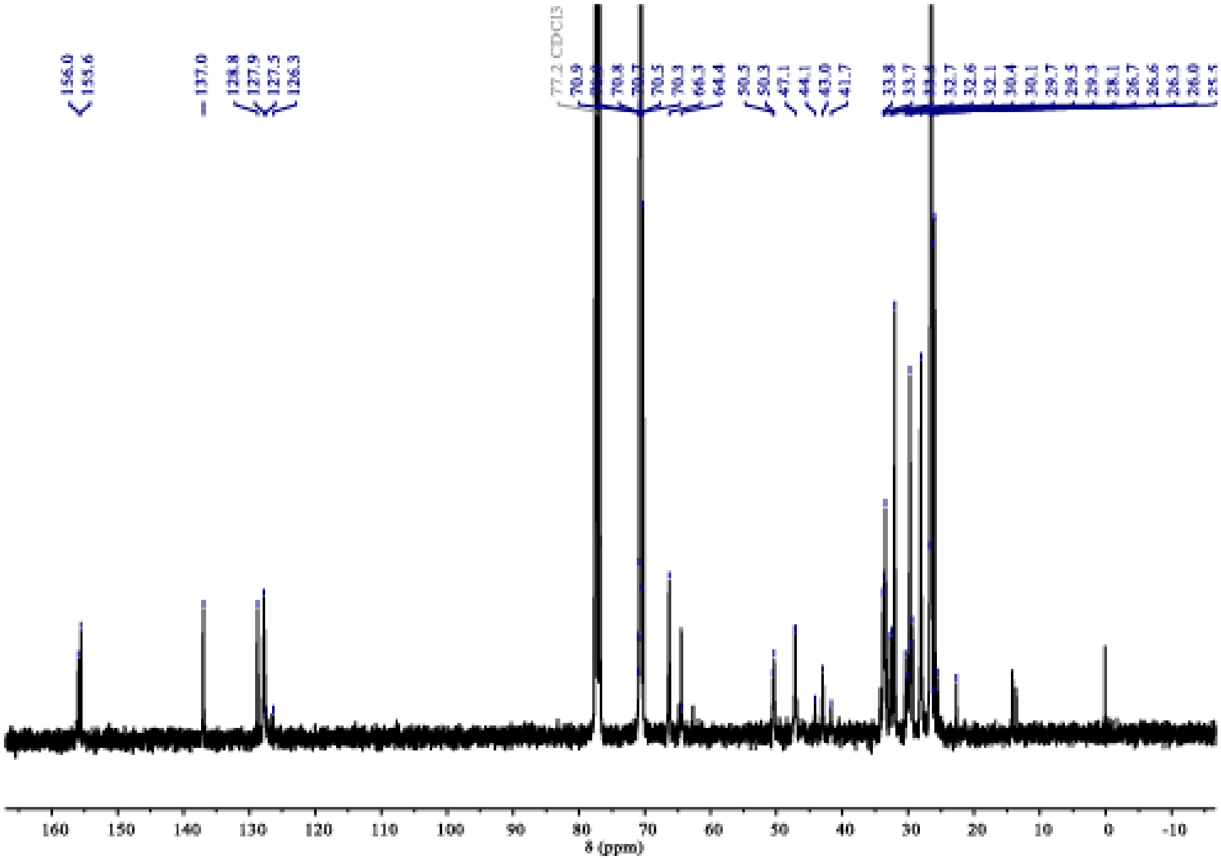

Figure S 36 $^{13}C\{H\}$ NMR spectrum of **SPE8** (100 MHz, $CDCl_3$, 298 K).

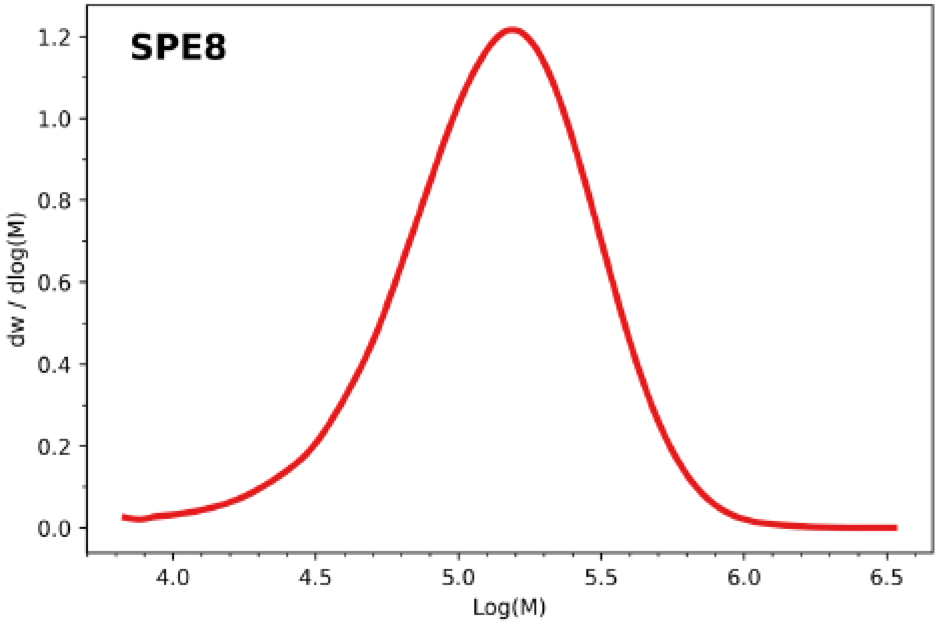

Figure S 37 GPC eluogram of **SPE8** (DMF, room temperature).

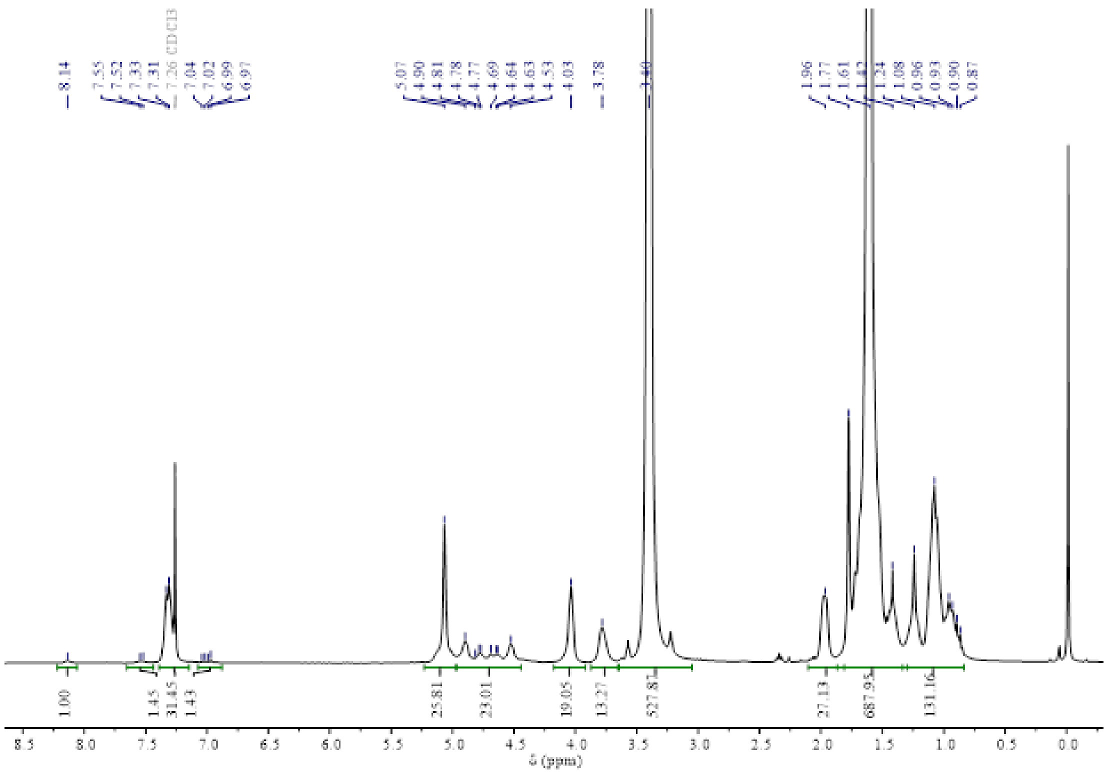

Figure S 38 ^{1}H NMR spectrum of **SPE9** (400 MHz, $CDCl_3$, 298 K).

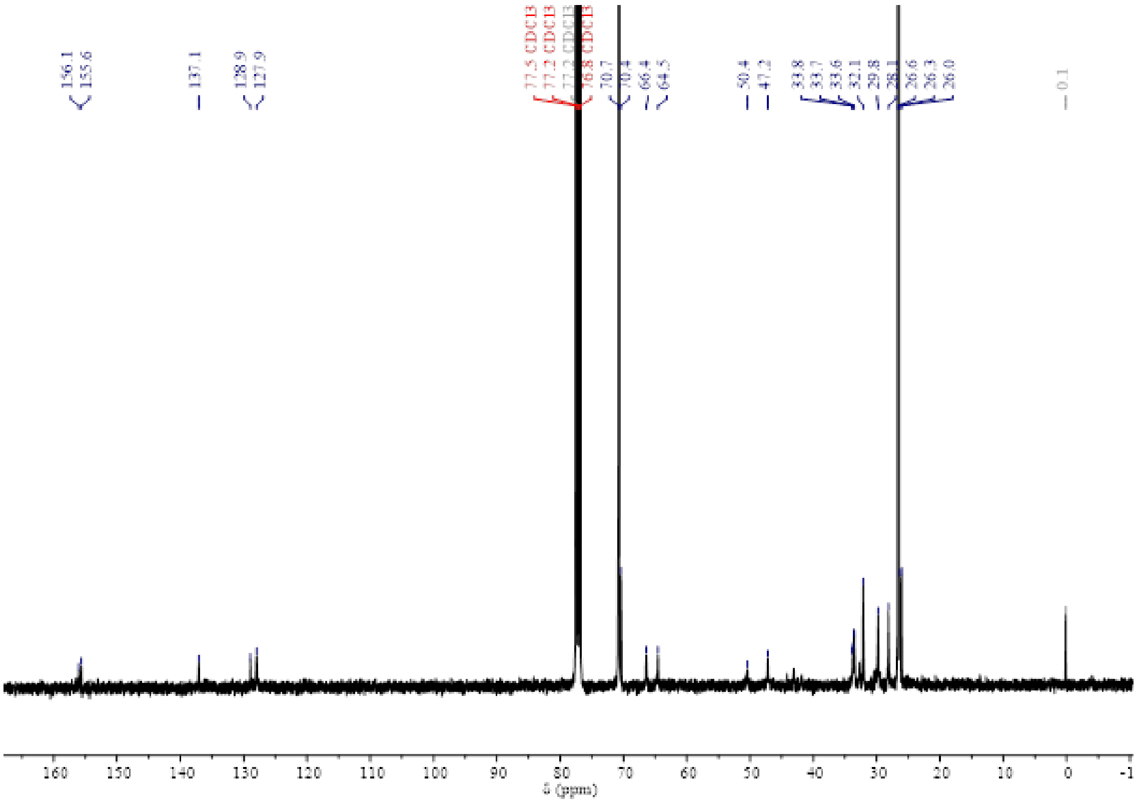

Figure S 39 $^{13}C\{H\}$ NMR spectrum of **SPE9** (100 MHz, $CDCl_3$, 298 K).

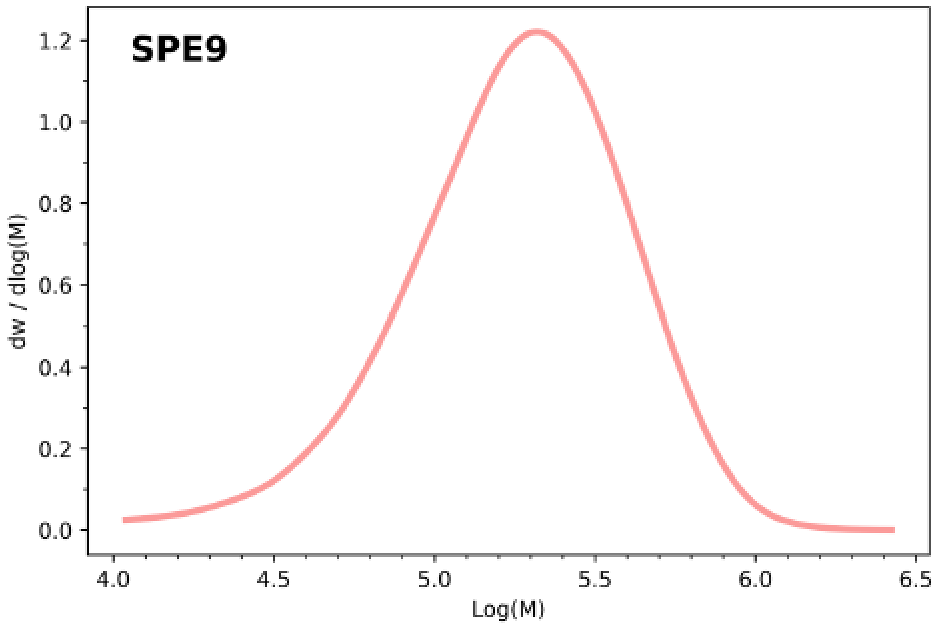

Figure S 40 GPC eluogram of **SPE9** (DMF, room temperature).

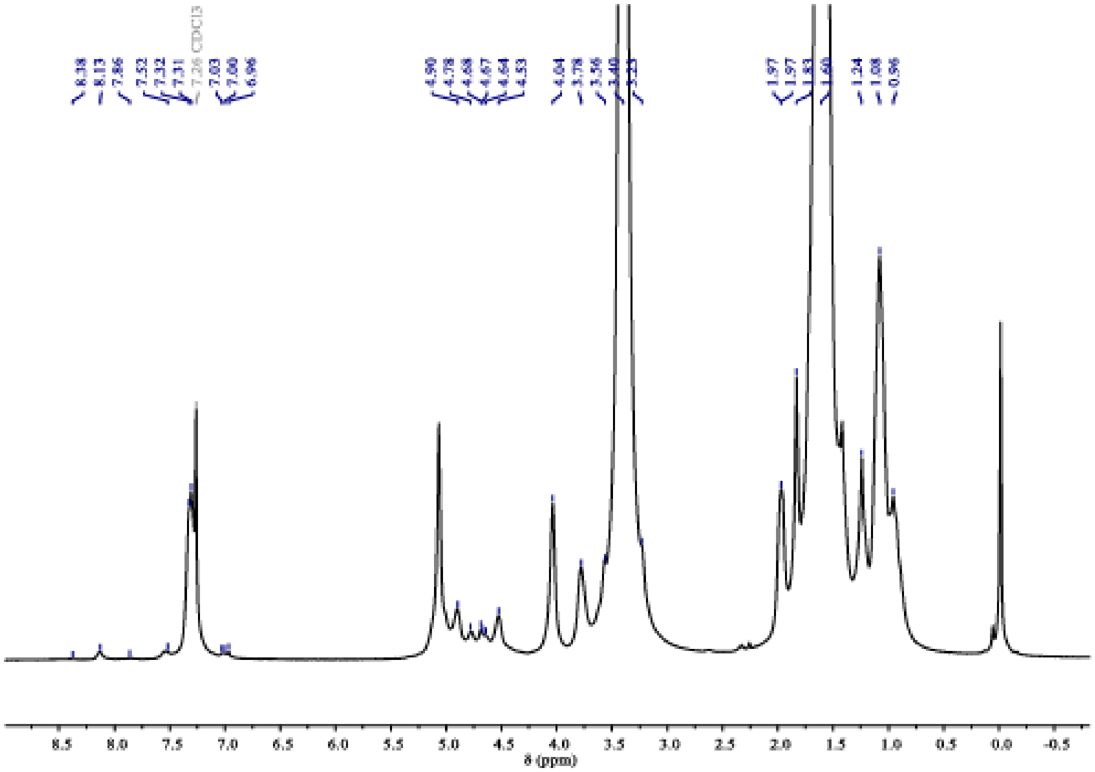

Figure S 41 ^{1}H NMR spectrum of **SPE10** (400 MHz, $CDCl_3$, 298 K).

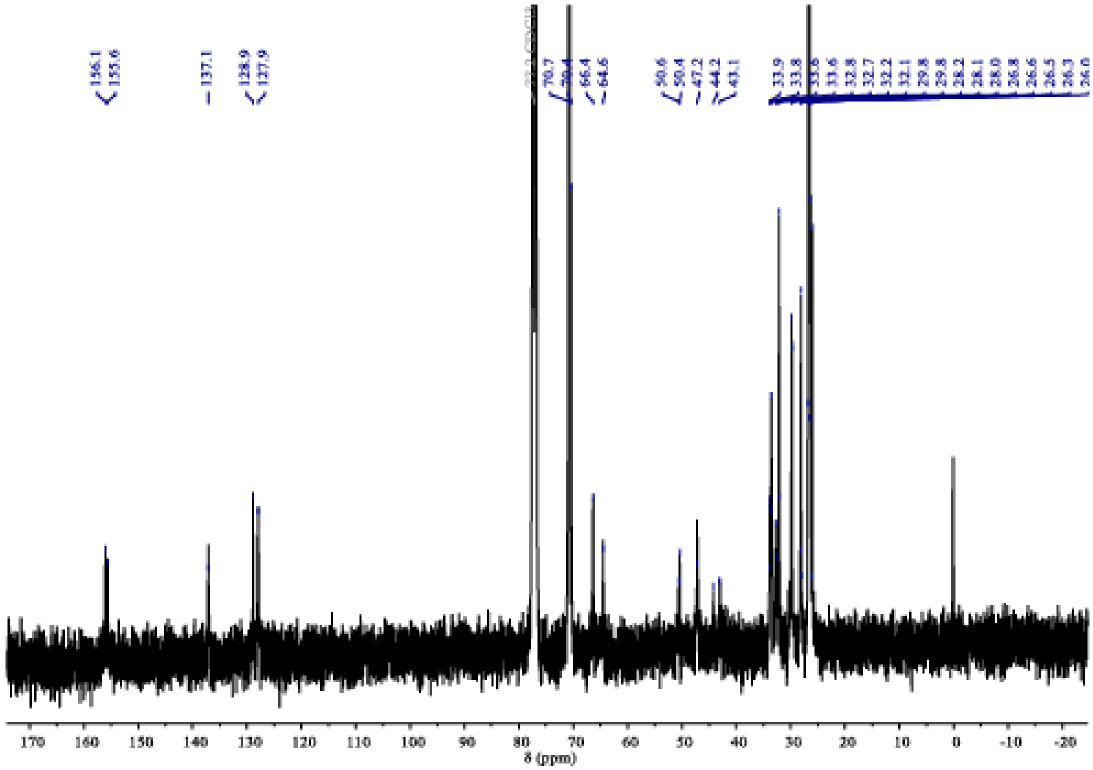

Figure S 42 $^{13}C\{H\}$ NMR spectrum of **SPE10** (100 MHz, $CDCl_3$, 298 K).

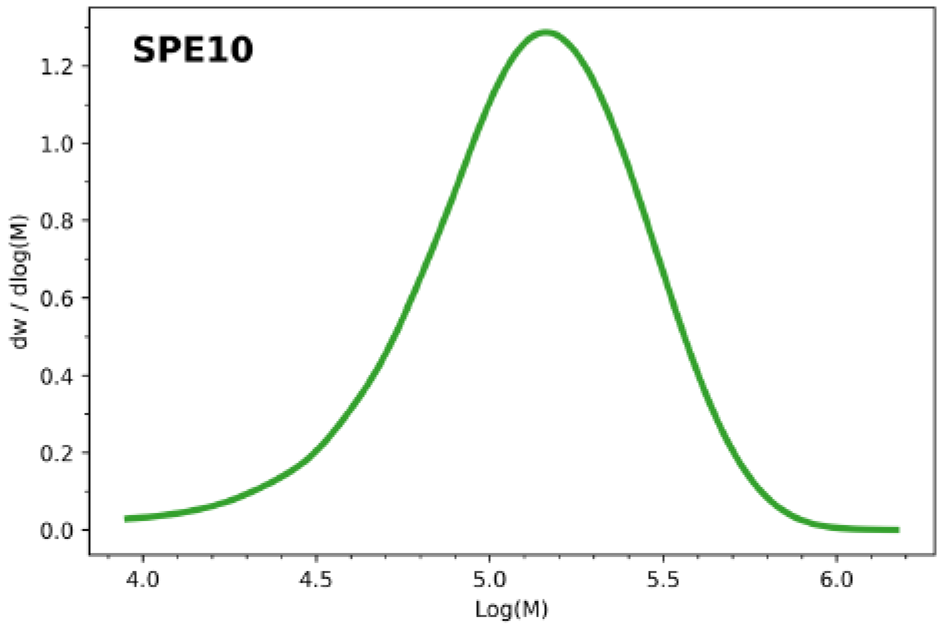

Figure S 43 GPC eluogram of **SPE10** (DMF, room temperature).

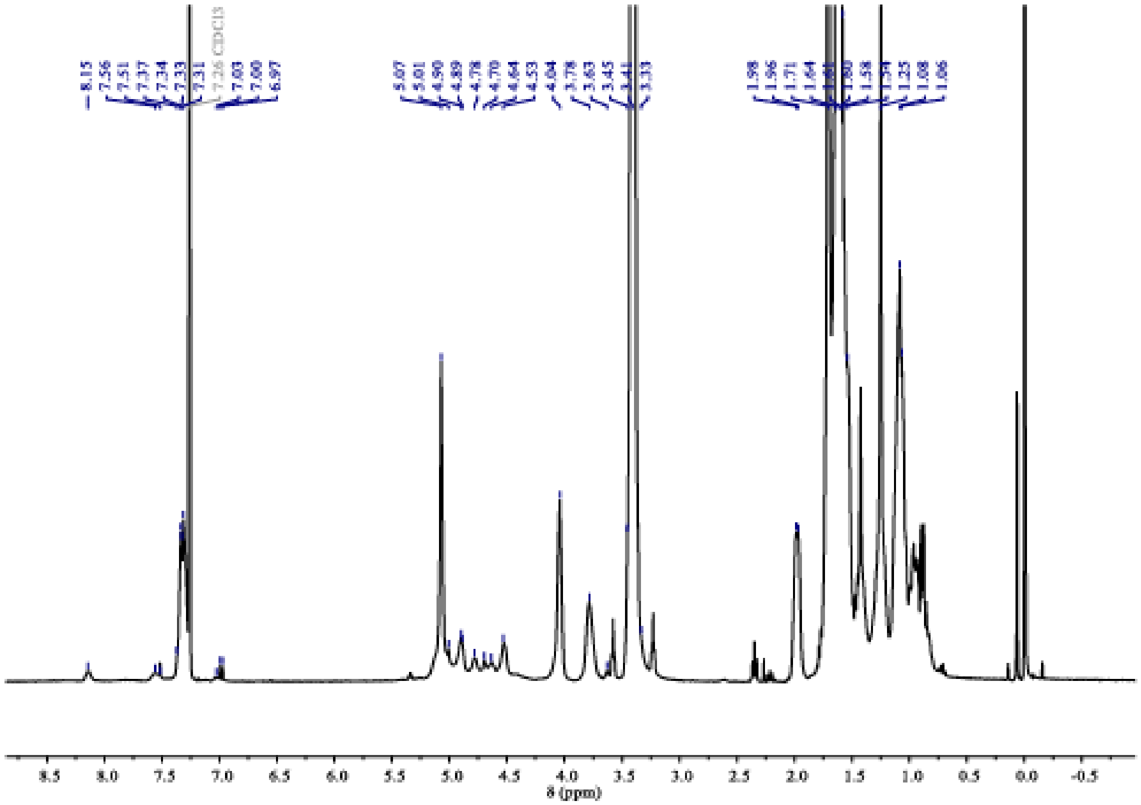

Figure S 44 ^{1}H NMR spectrum of **SPE11** (400 MHz, $CDCl_3$, 298 K).

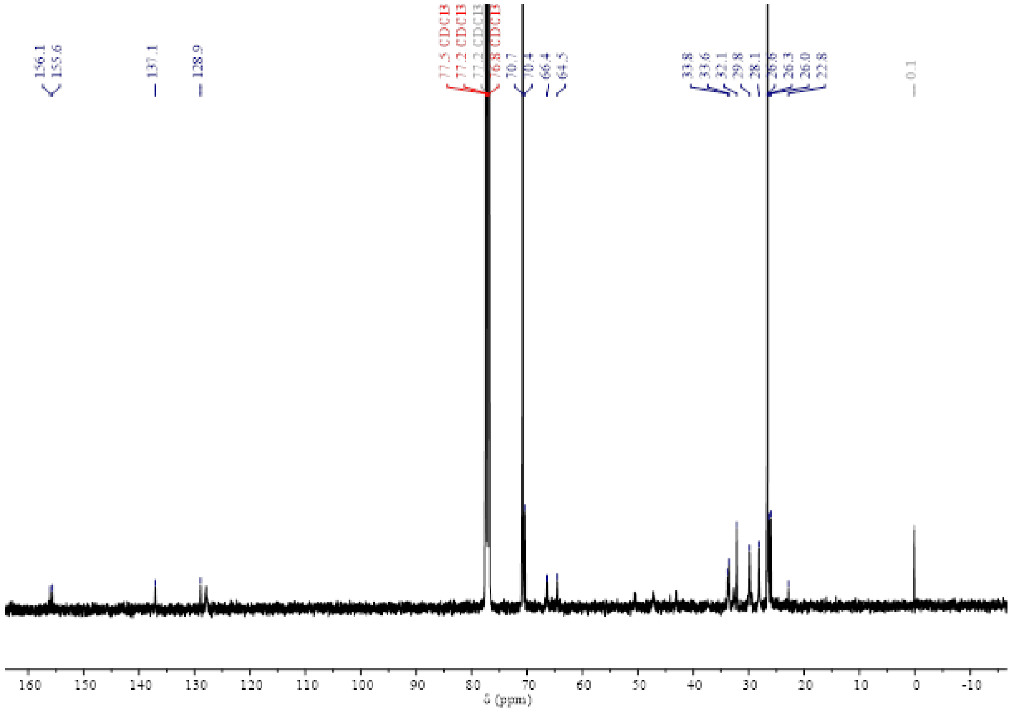

Figure S 45 ^{13}C{H} NMR spectrum of **SPE11** (100 MHz, $CDCl_3$, 298 K).

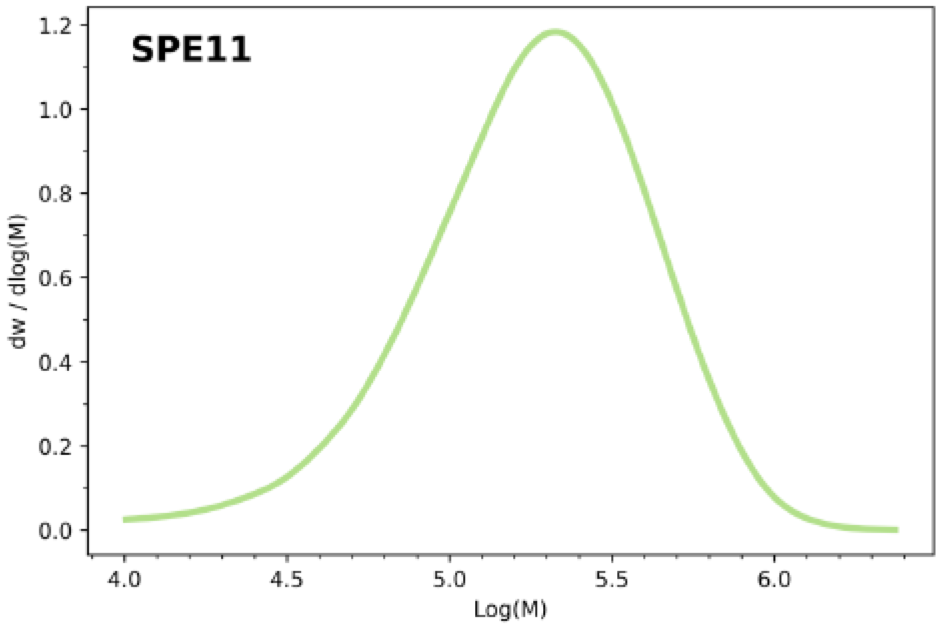

Figure S 46 GPC eluogram of **SPE11** (DMF, room temperature).

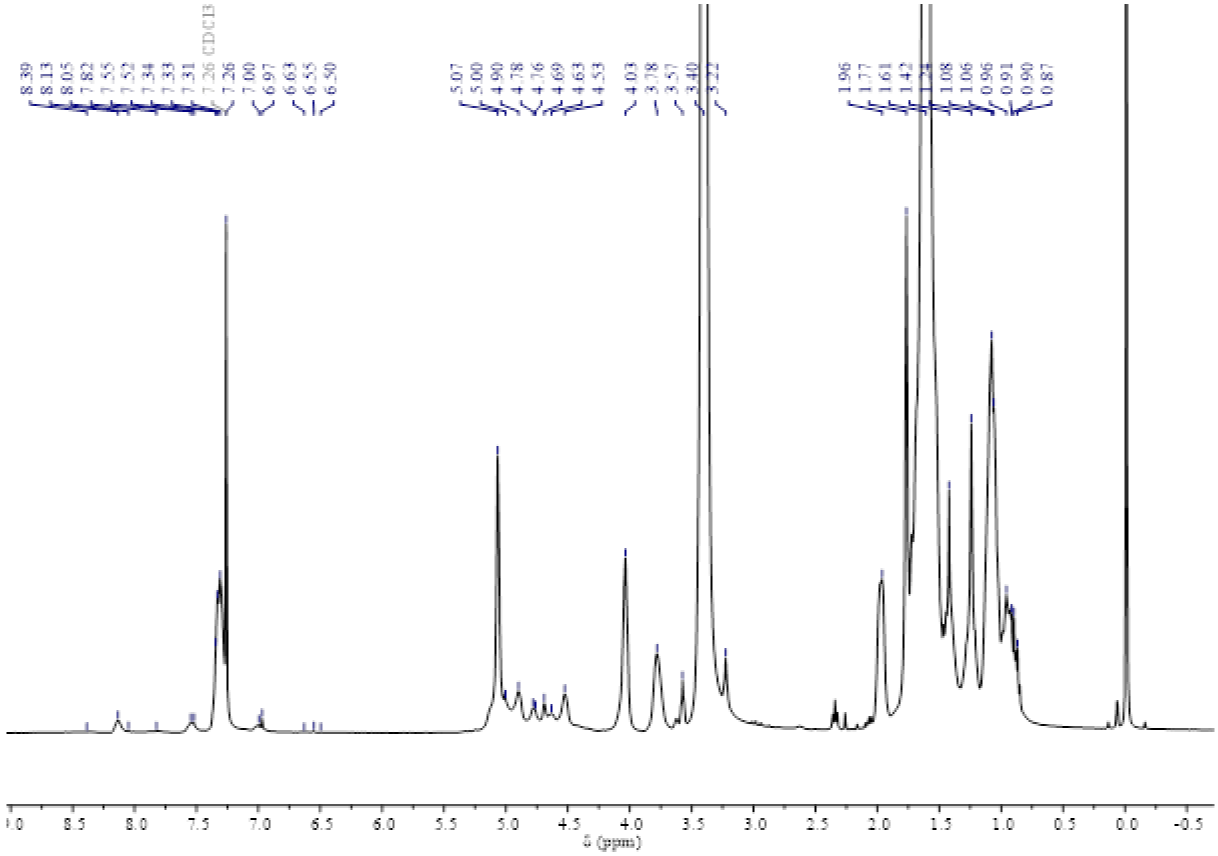

Figure S 47 ^{1}H NMR spectrum of **SPE12** (400 MHz, $CDCl_3$, 298 K).

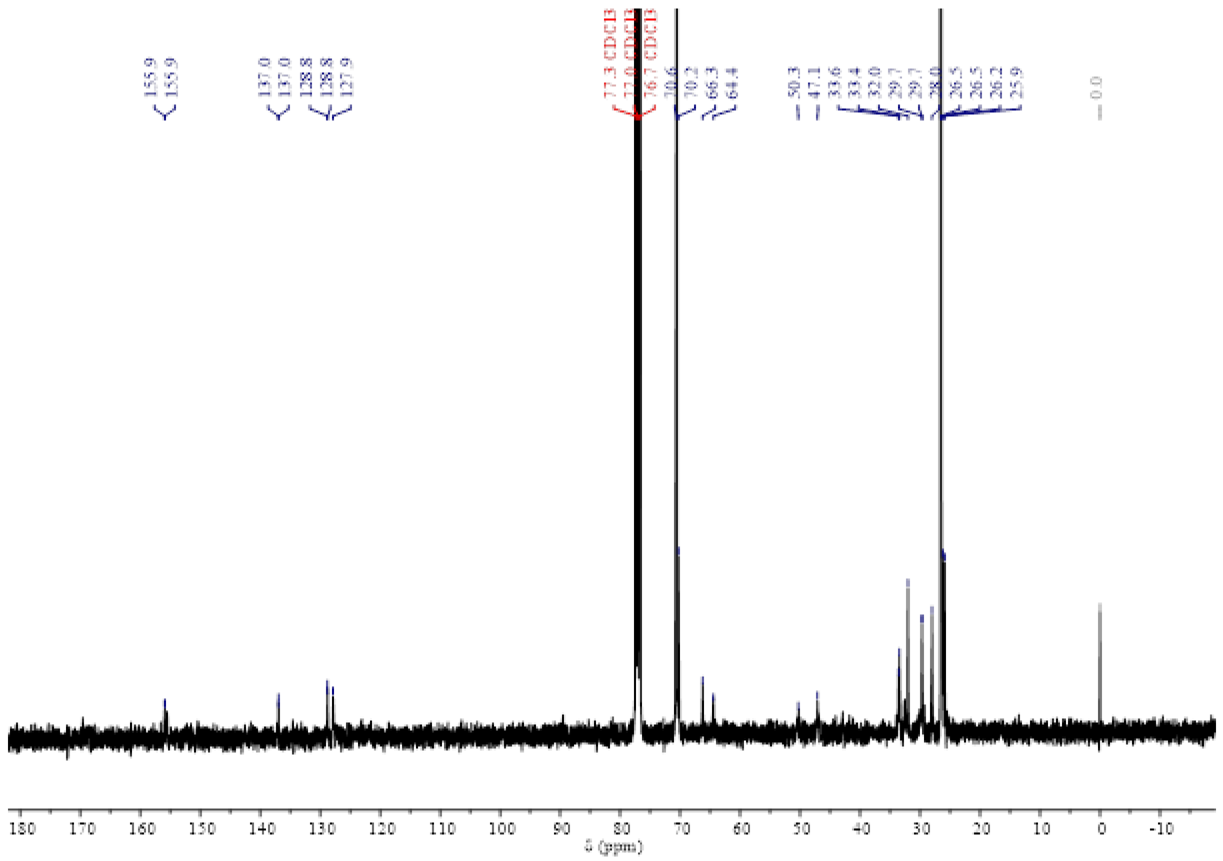

Figure S 48 $^{13}C\{H\}$ NMR spectrum of **SPE12** (100 MHz, $CDCl_3$, 298 K).

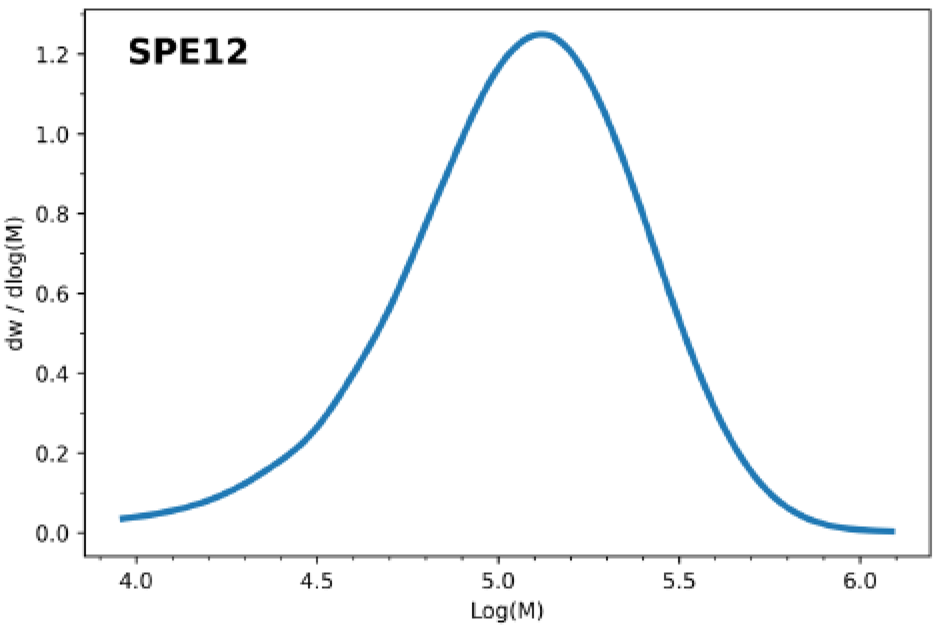

Figure S 49 GPC eluogram of **SPE12** (DMF, room temperature).

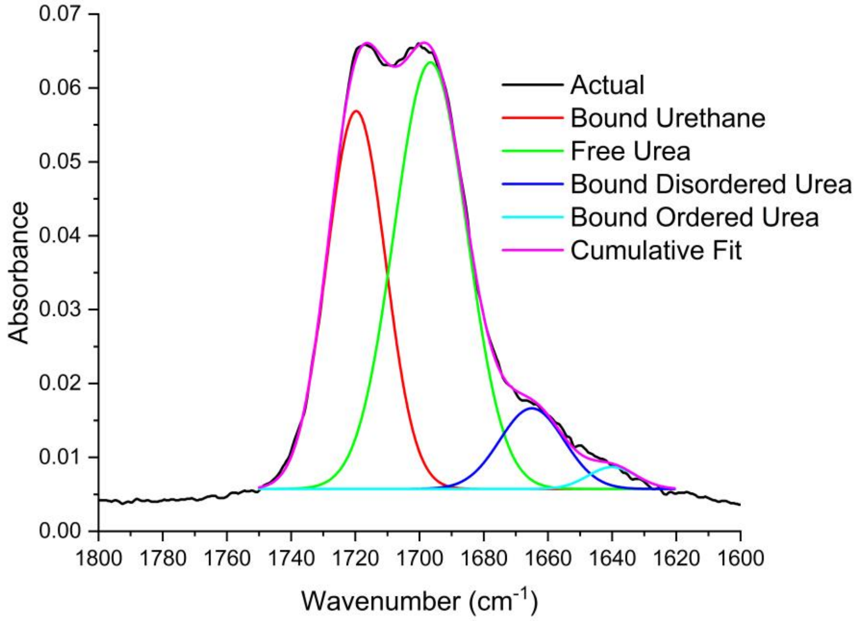

Figure S 50 Example of the deconvolution analysis of IR spectroscopic data; shown for **SPE1** at 25 °C in the carbonyl region 1800-1600 cm^{-1}.

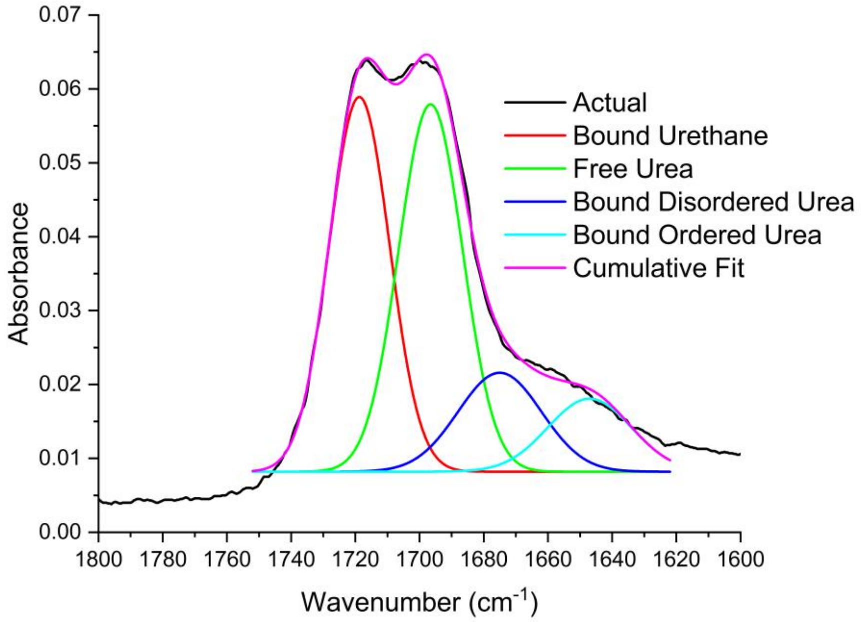

Figure S 51 Example of the deconvolution analysis of IR spectroscopic data; shown for **SPE6** at 25 °C in the carbonyl region 1800-1600 cm^{-1}.

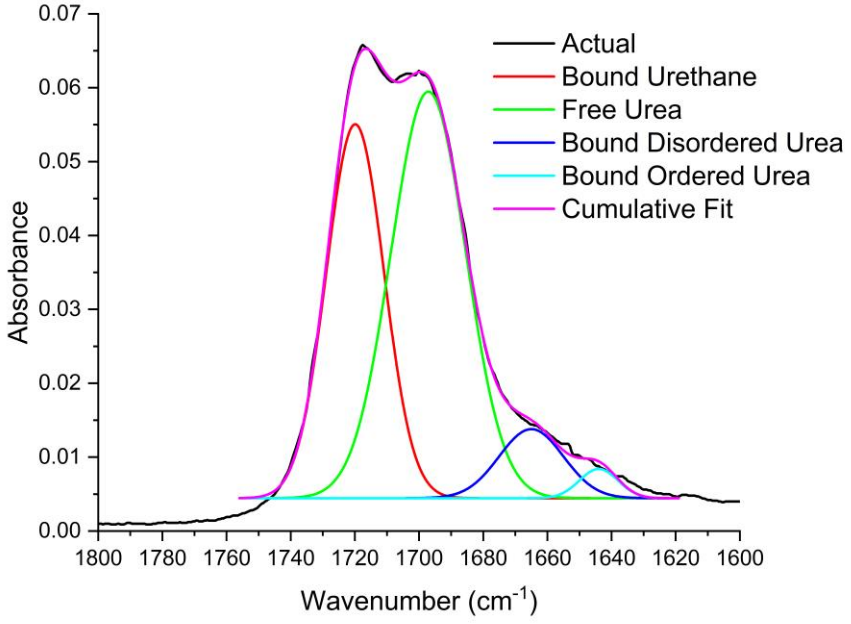

Figure S 52 Example of the deconvolution analysis of IR spectroscopic data; shown for **SPE7** at 25 °C in the carbonyl region 1800-1600 cm^{-1}.

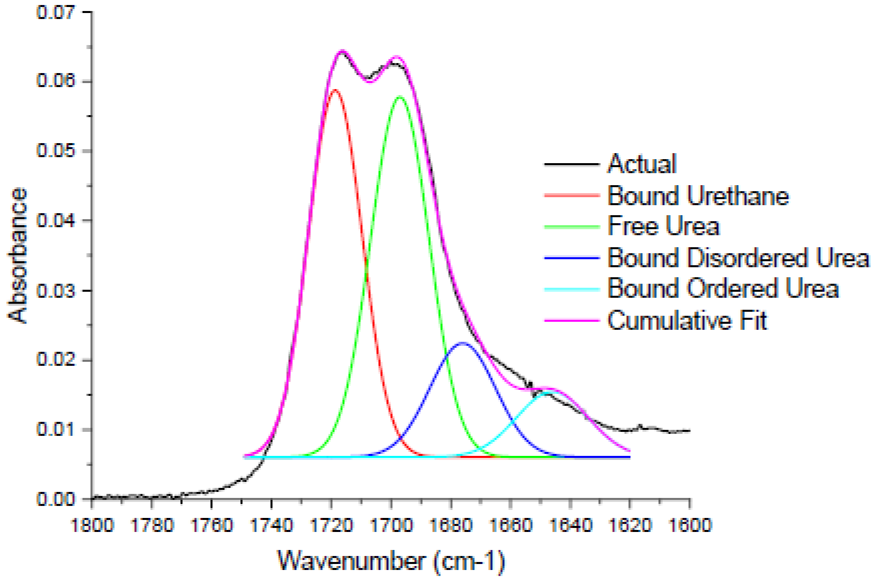

Figure S 53 Example of the deconvolution analysis of IR spectroscopic data; shown for **SPE12** at 25 °C in the carbonyl region 1800-1600 cm^{-1}.

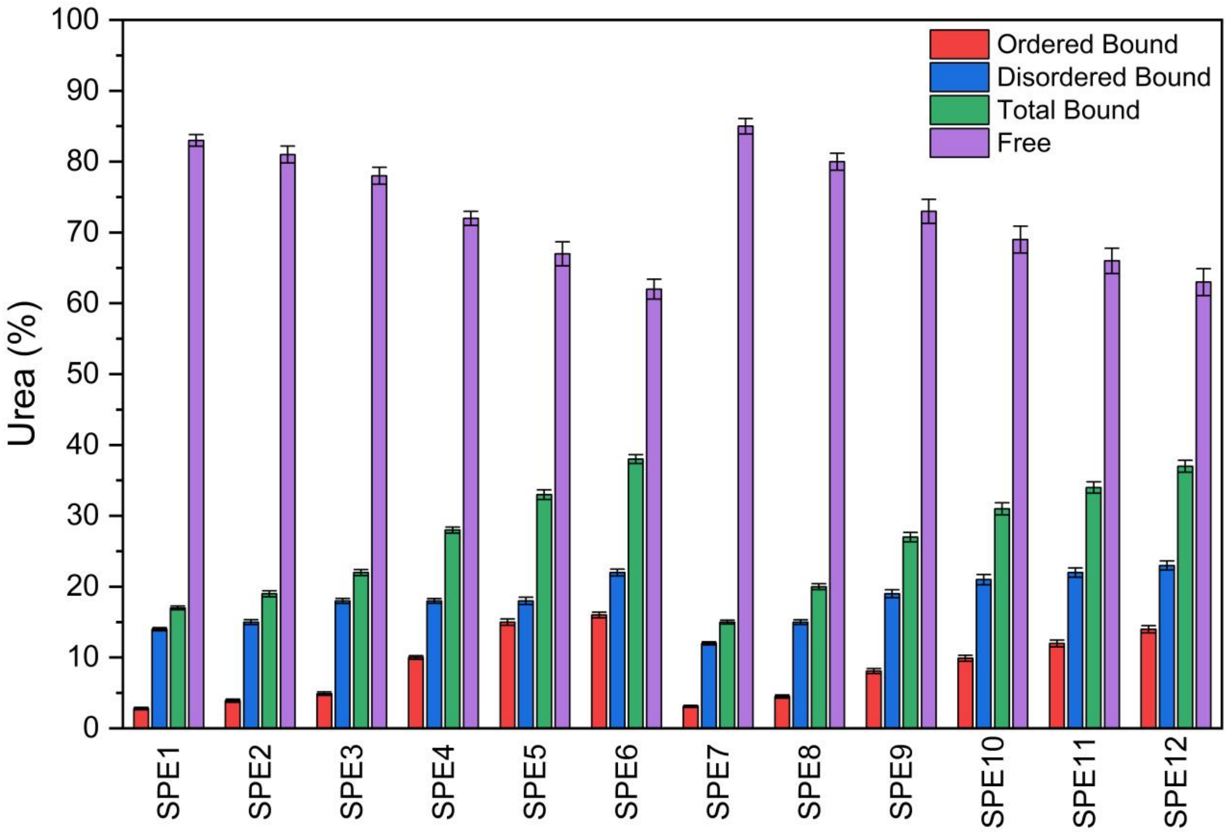

Figure S 54 The urea deconvolution analysis of IR spectroscopic data

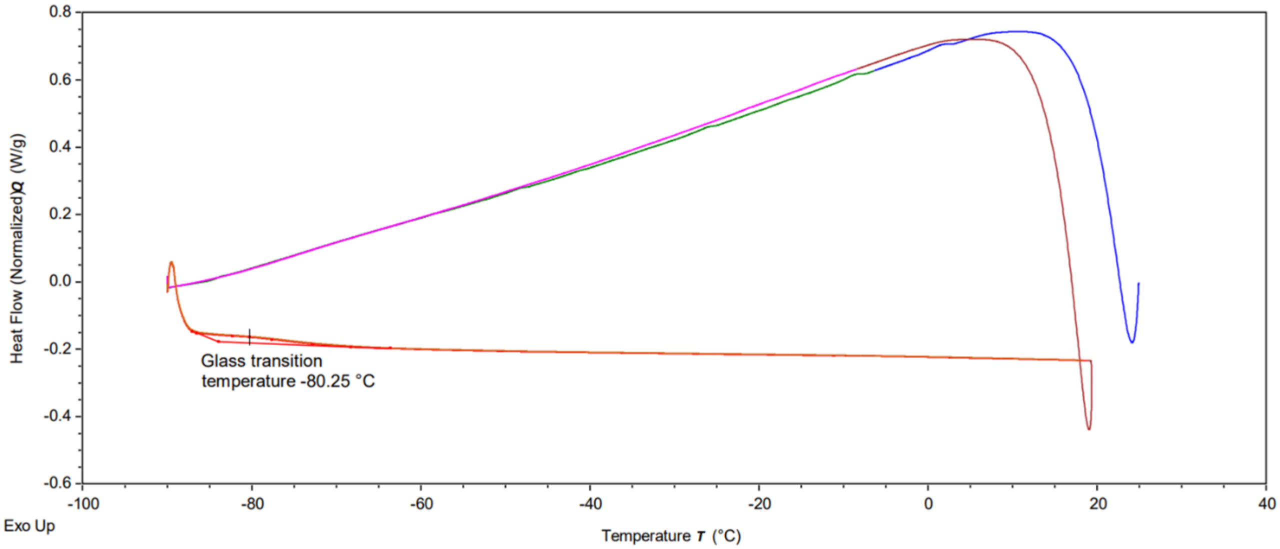

Figure S 55 DSC analysis of **SPE5**

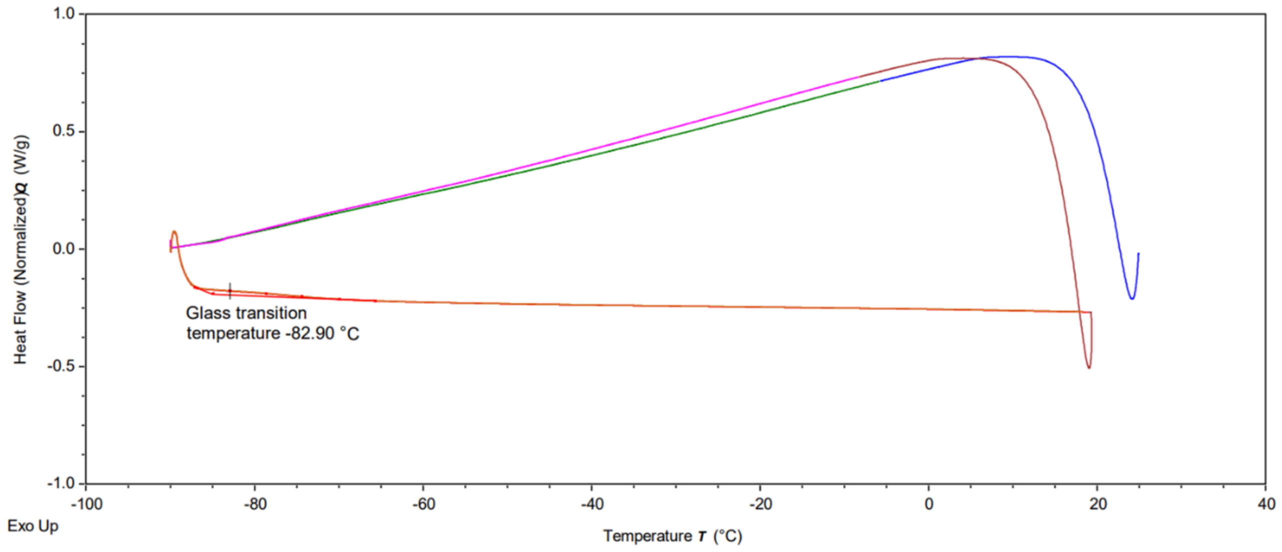

Figure S 56 DSC analysis of **SPE6**

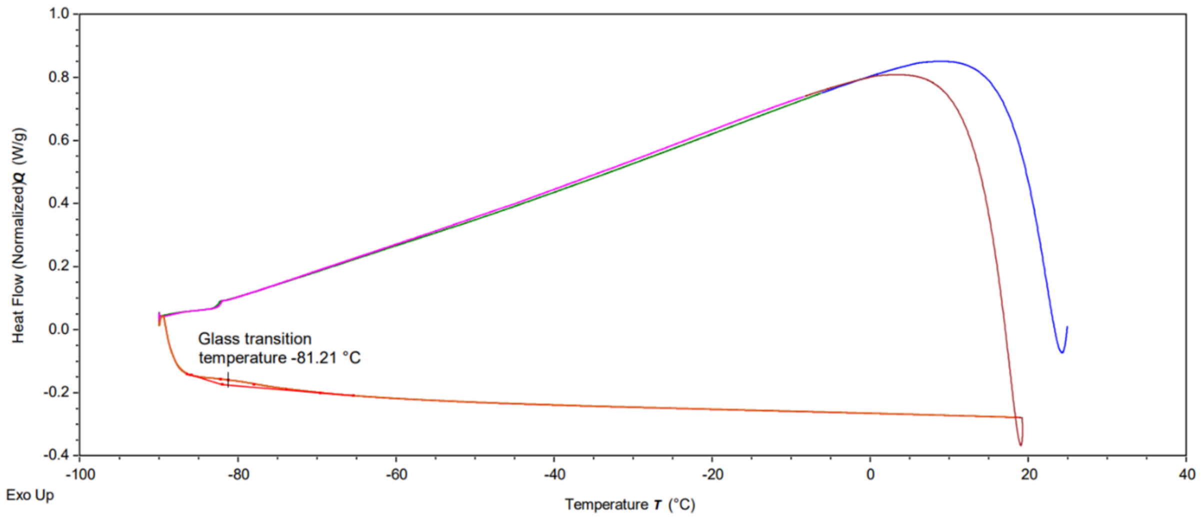

Figure S 57 DSC analysis of **SPE8**

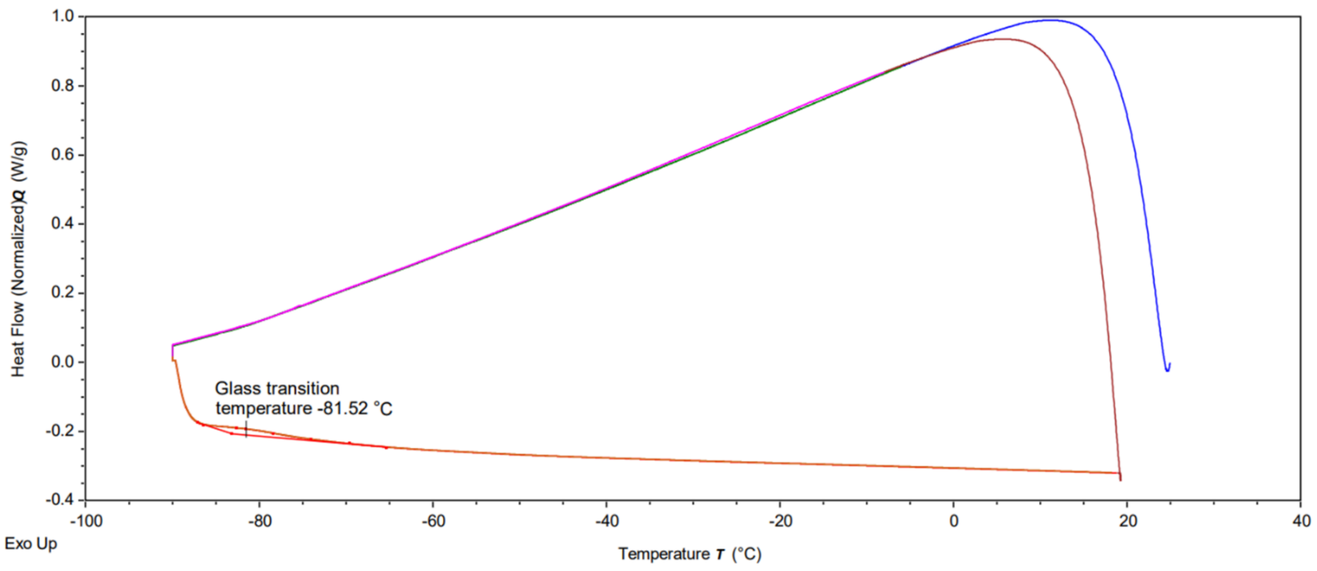

Figure S 58 DSC analysis of **SPE9**

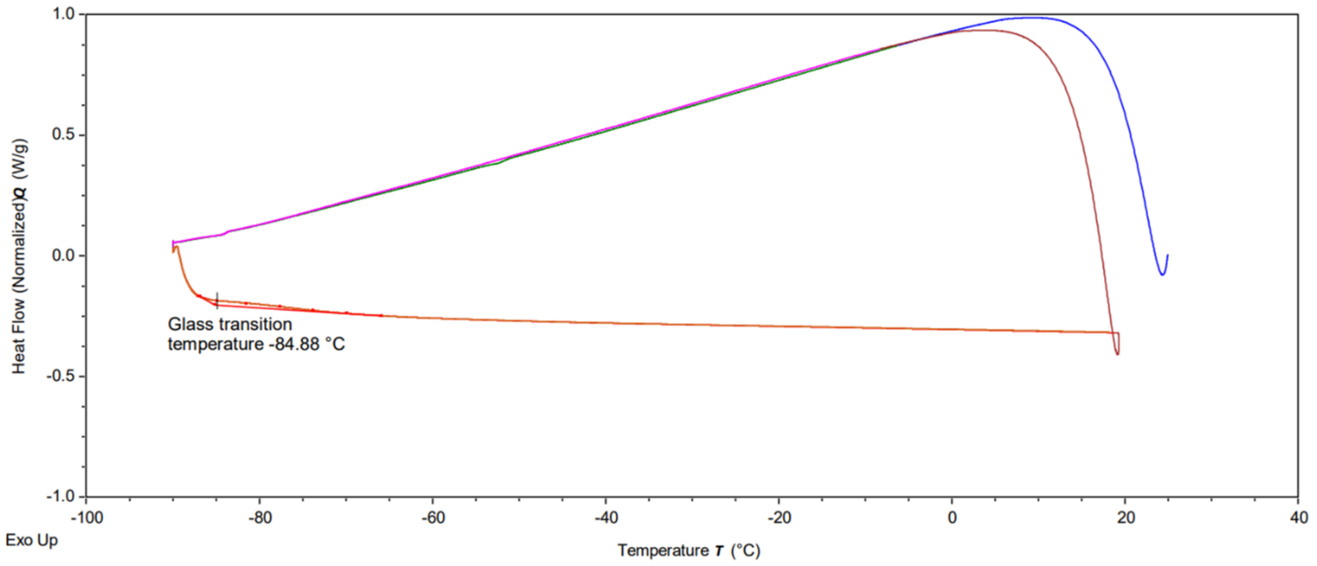

Figure S 59 DSC analysis of **SPE10**

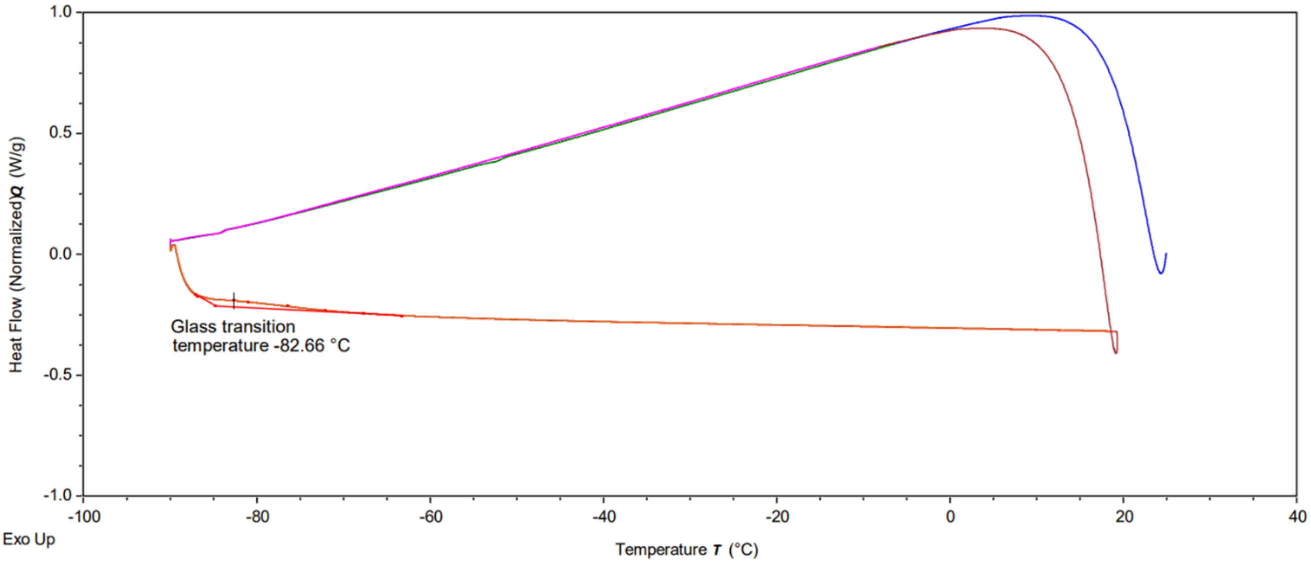

Figure S 60 DSC analysis of **SPE11**

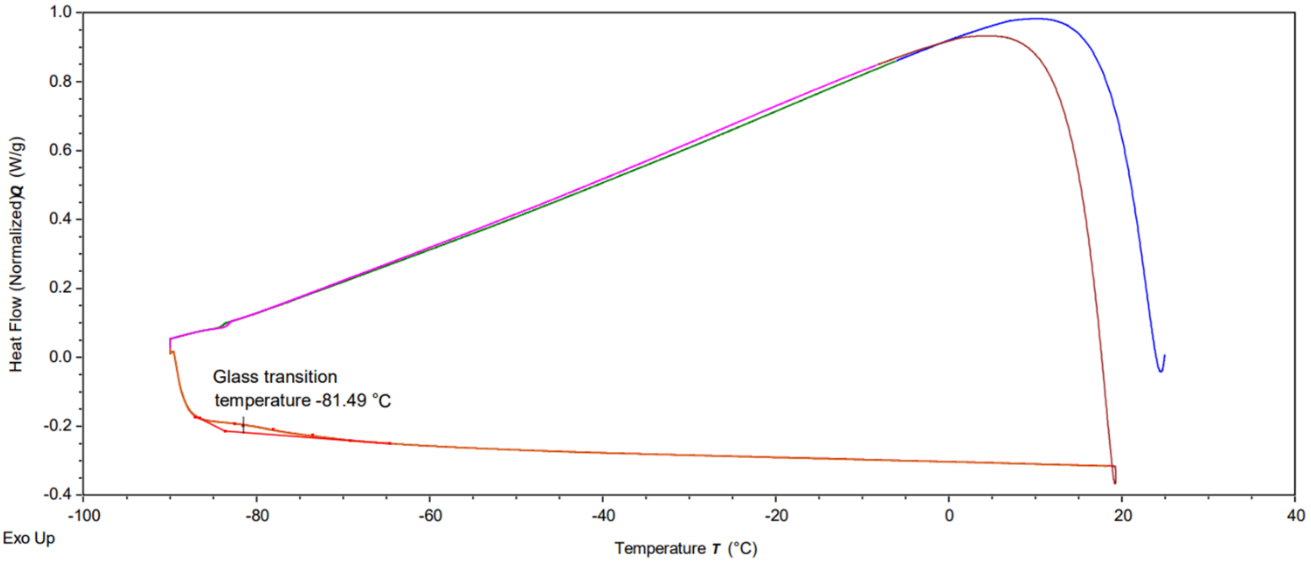

Figure S 61 DSC analysis of **SPE12**

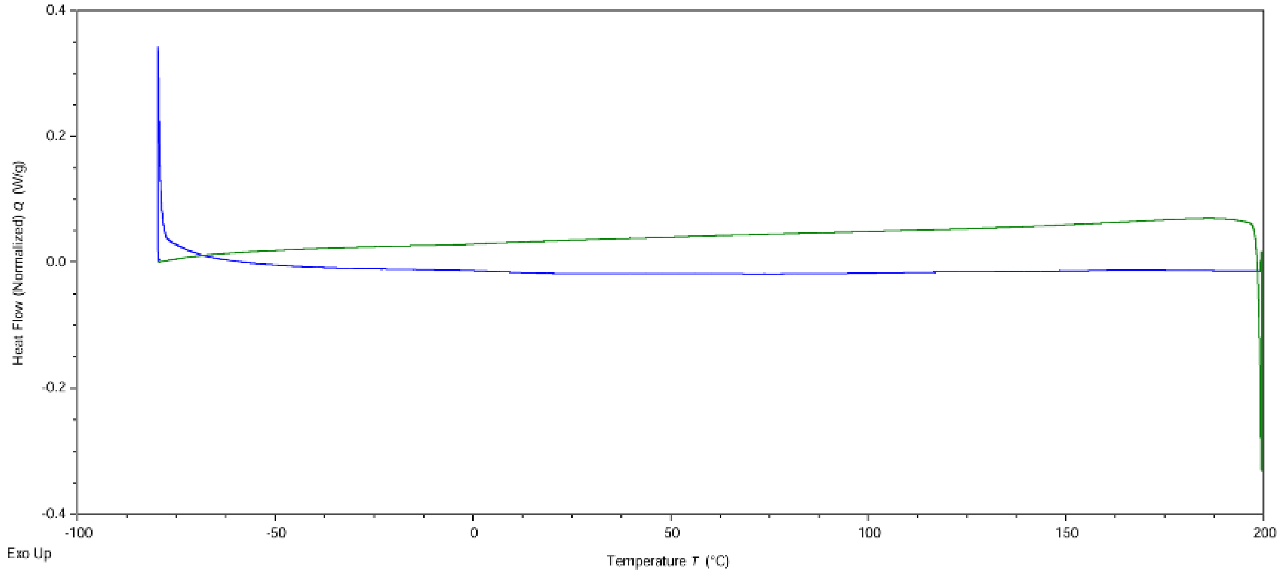

Figure S 62 DSC analysis of **SPE1** from -70 to 200 °C

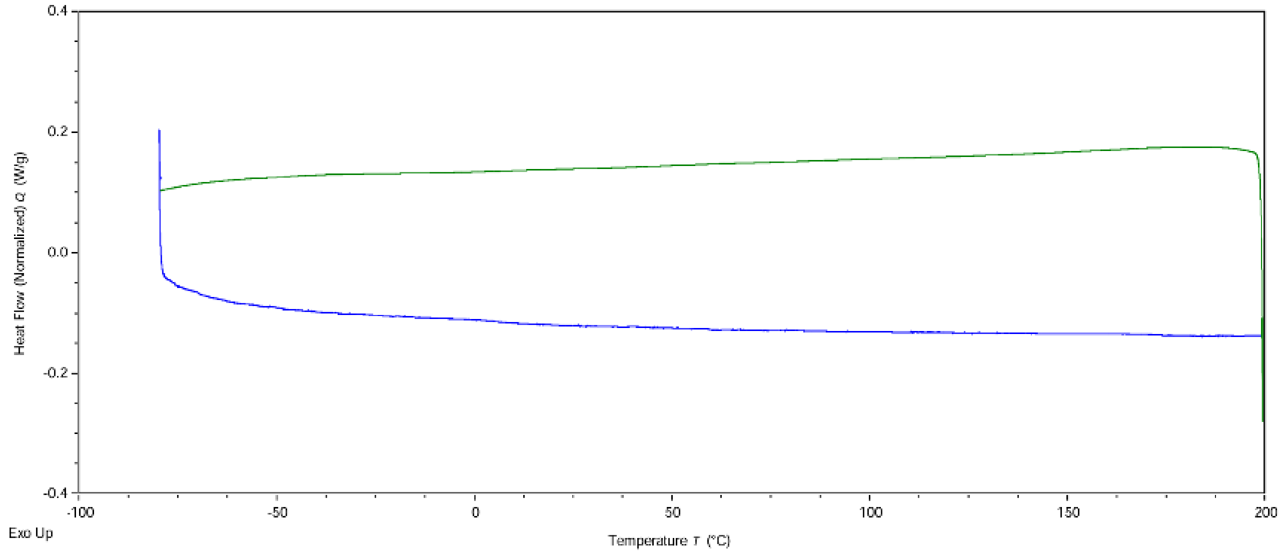

Figure S 63 DSC analysis of **SPE2** from -70 to 200 °C

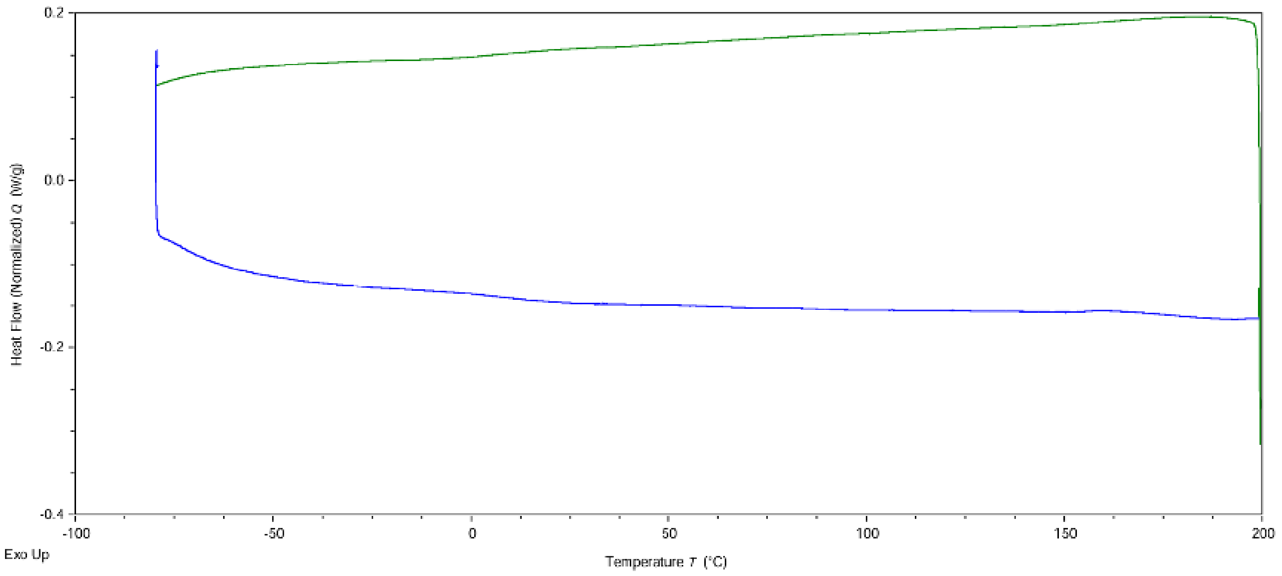

Figure S 64 DSC analysis of **SPE3** from -70 to 200 °C

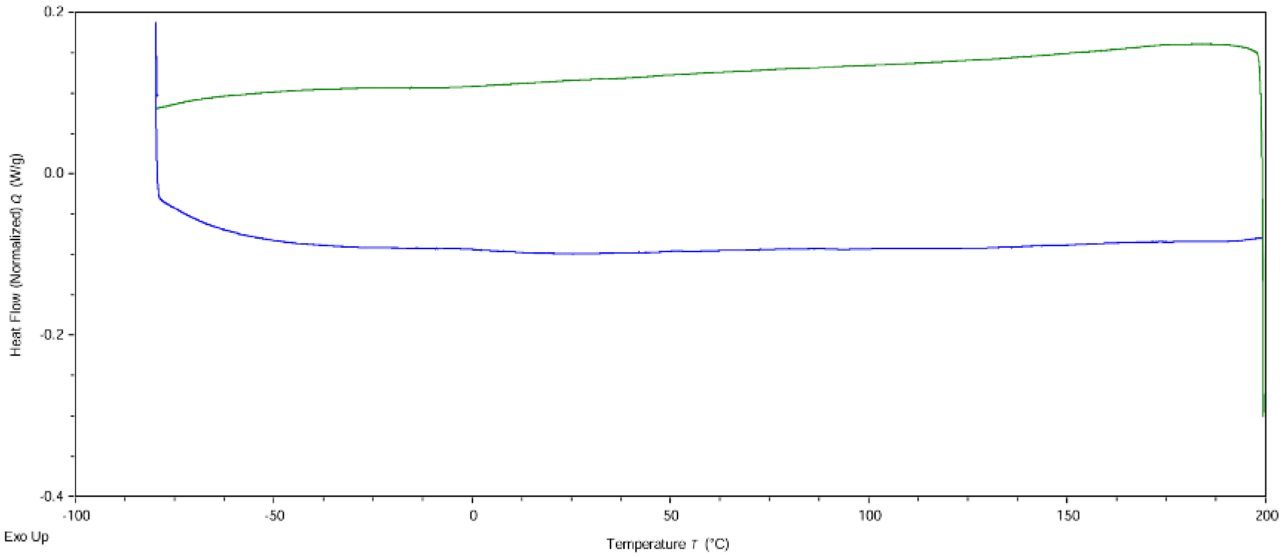

Figure S 65 DSC analysis of **SPE4** from -70 to 200 °C

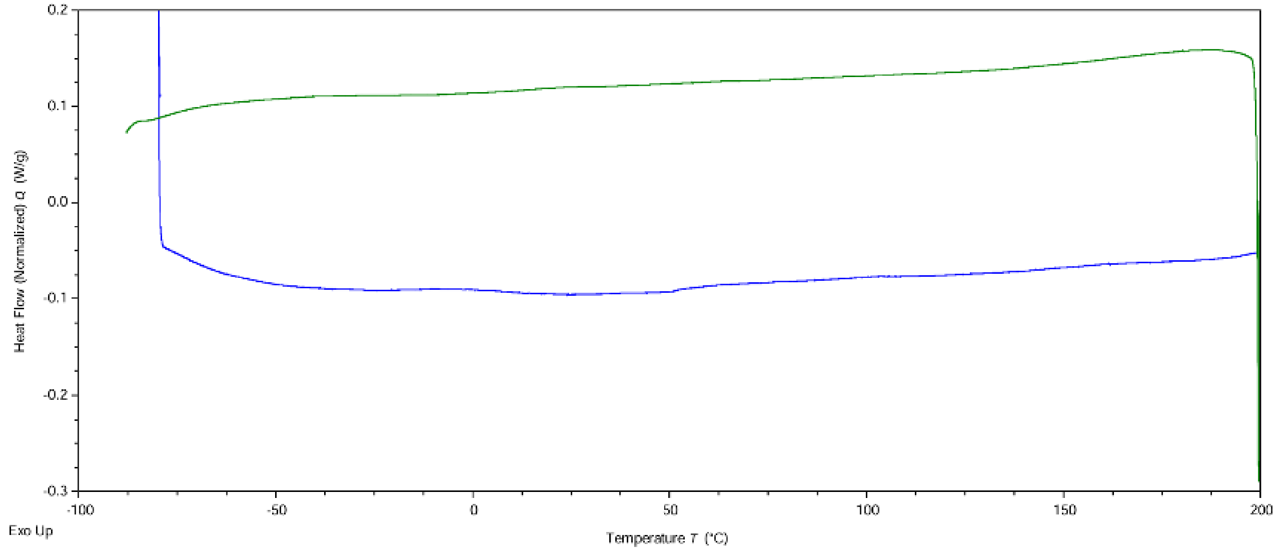

Figure S 66 DSC analysis of **SPE5** from -70 to 200 °C

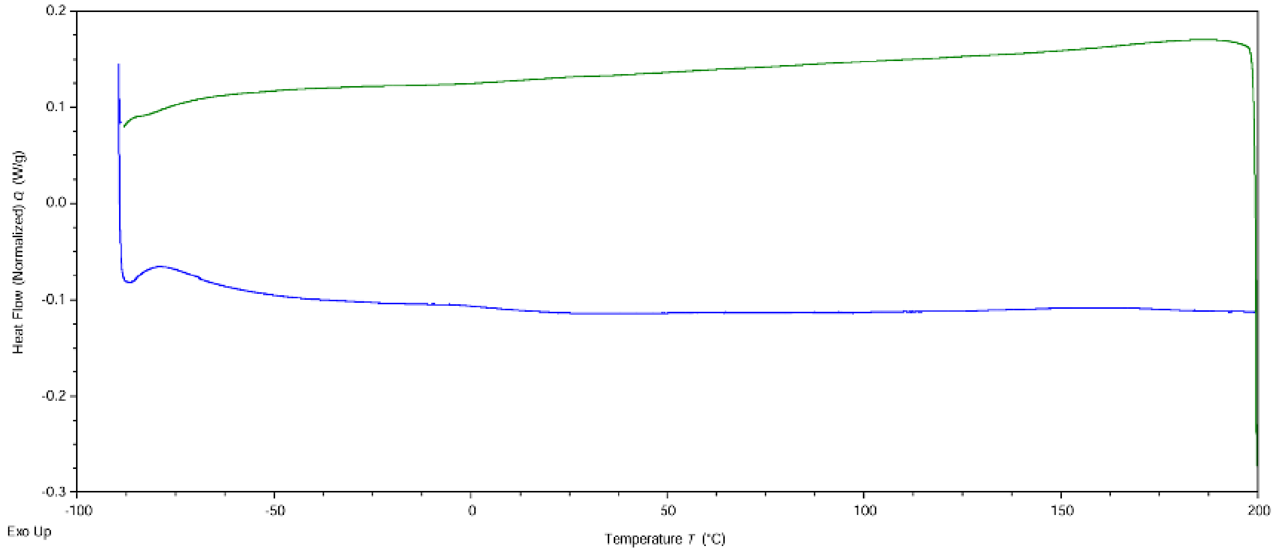

Figure S 67 DSC analysis of **SPE6** from -70 to 200 °C

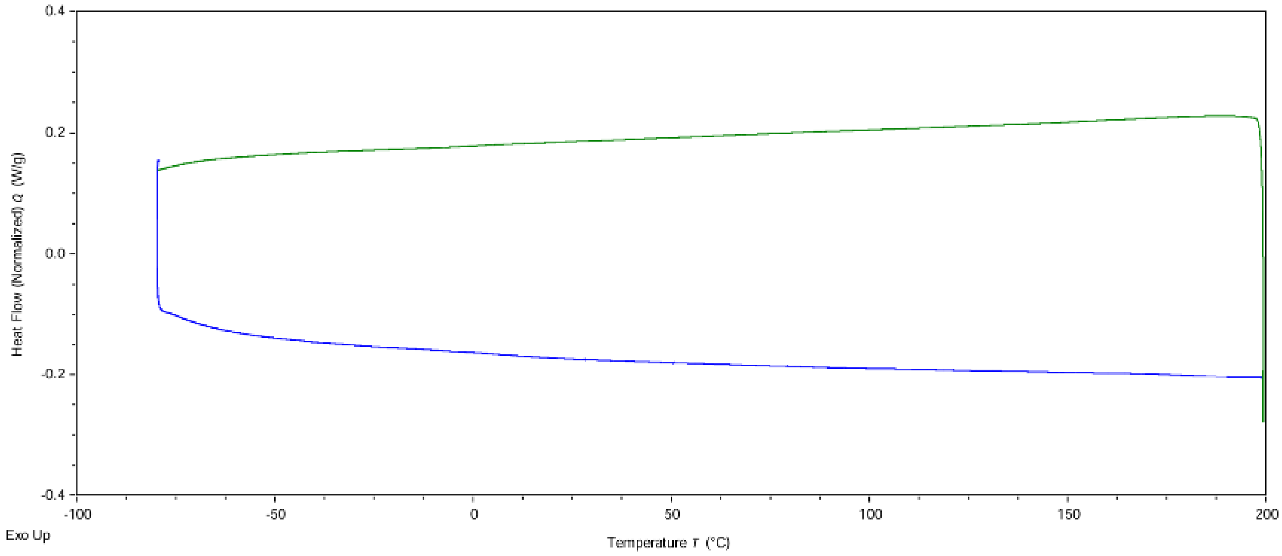

Figure S 68 DSC analysis of **SPE7** from -70 to 200 °C

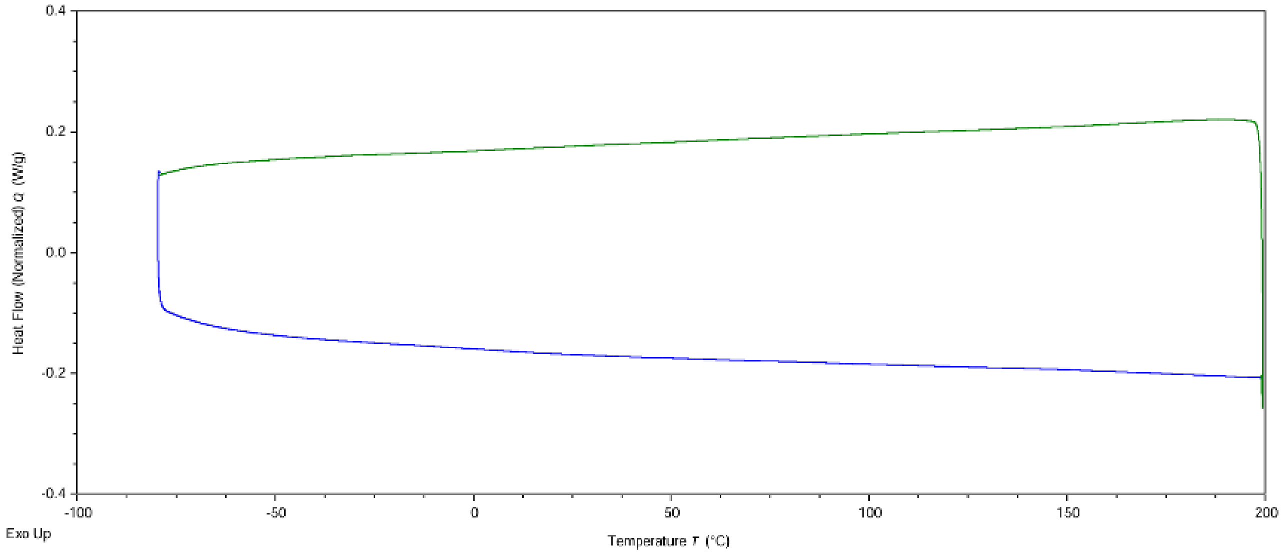

Figure S 69 DSC analysis of **SPE8** from -70 to 200 °C

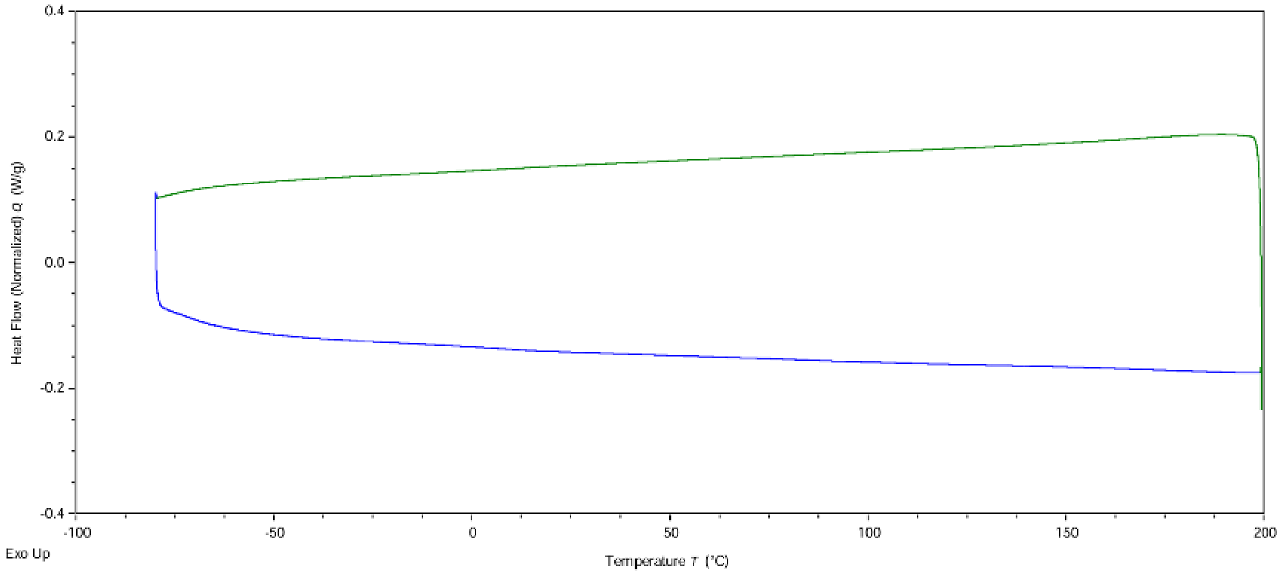

Figure S 70 DSC analysis of **SPE9** from -70 to 200 °C

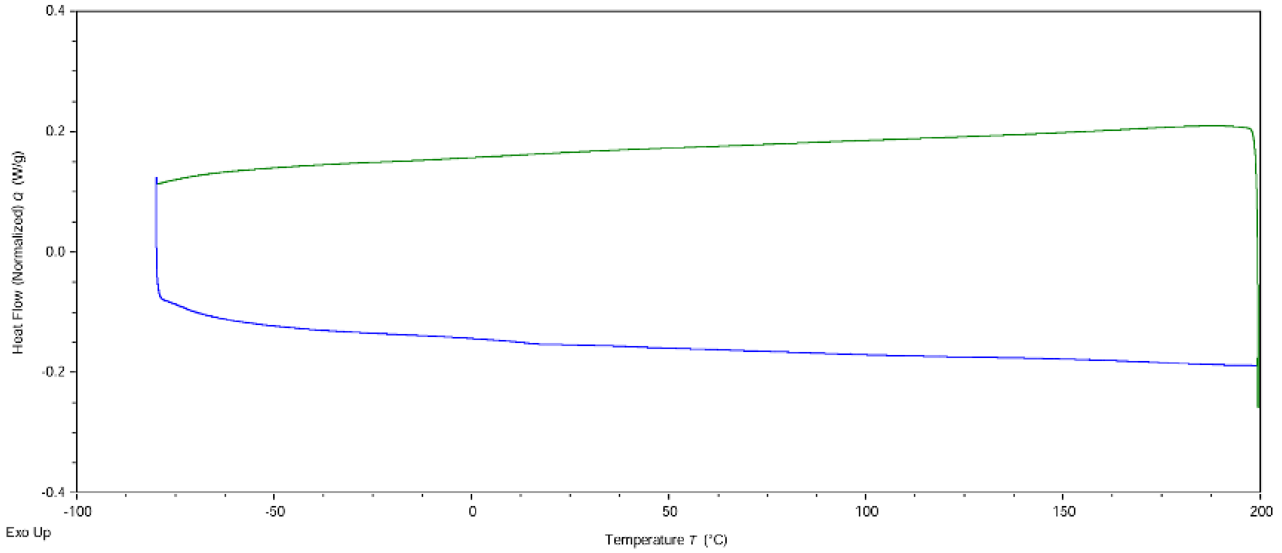

Figure S 71 DSC analysis of **SPE10** from -70 to 200 °C

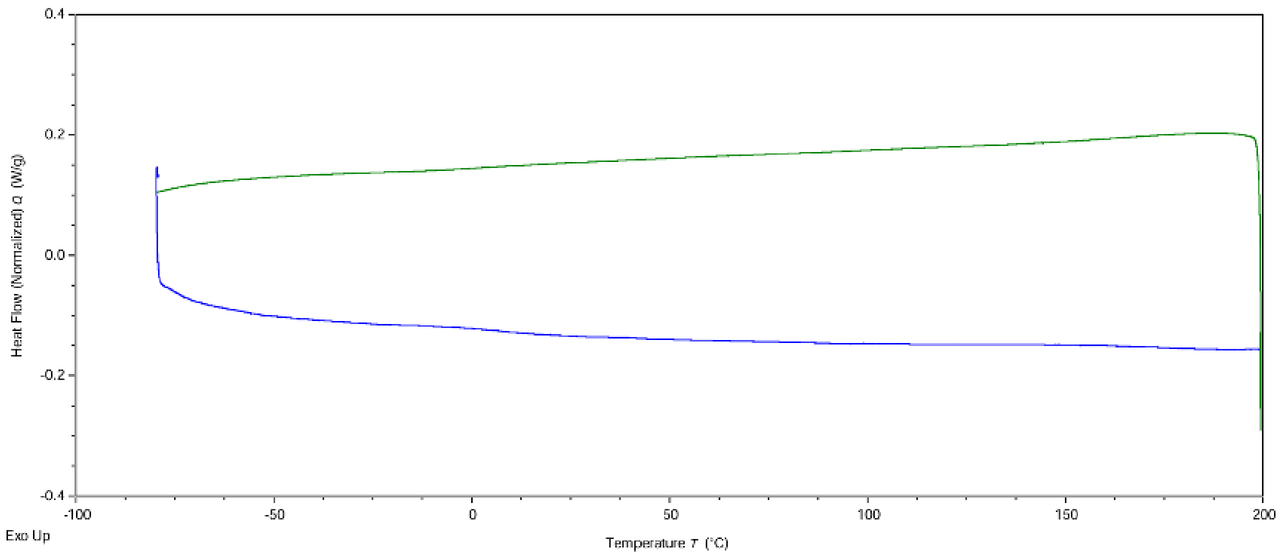

Figure S 72 DSC analysis of **SPE11** from -70 to 200 °C

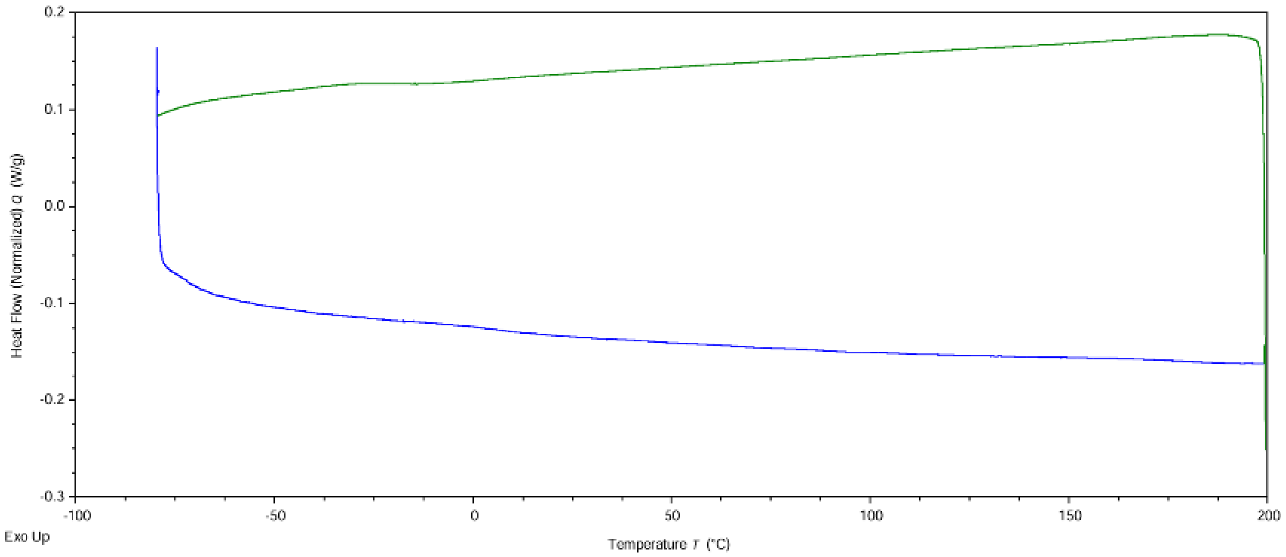

Figure S 73 DSC analysis of **SPE12** from -70 to 200 °C

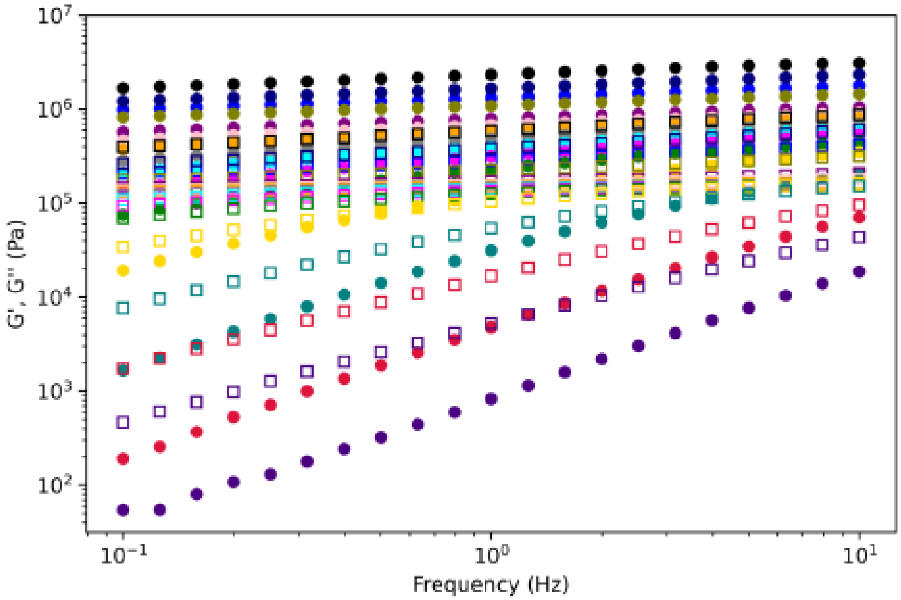

Figure S 74 Frequency sweeps of **SPE1** at 0 (black), 10 (red), 20 (blue), 30 (olive), 40 (navy), 50 (purple), 60 (pink), 70 (orange), 80 (gray), 90 (cyan), 100 (magenta), 110 (green), 120 (gold), 130 (teal), 140 (crimson), 150 °C (indigo). G' closed symbols and G'' open symbols frequency sweeps were performed between 0.1 to 10 Hz at an applied strain of 0.1%.

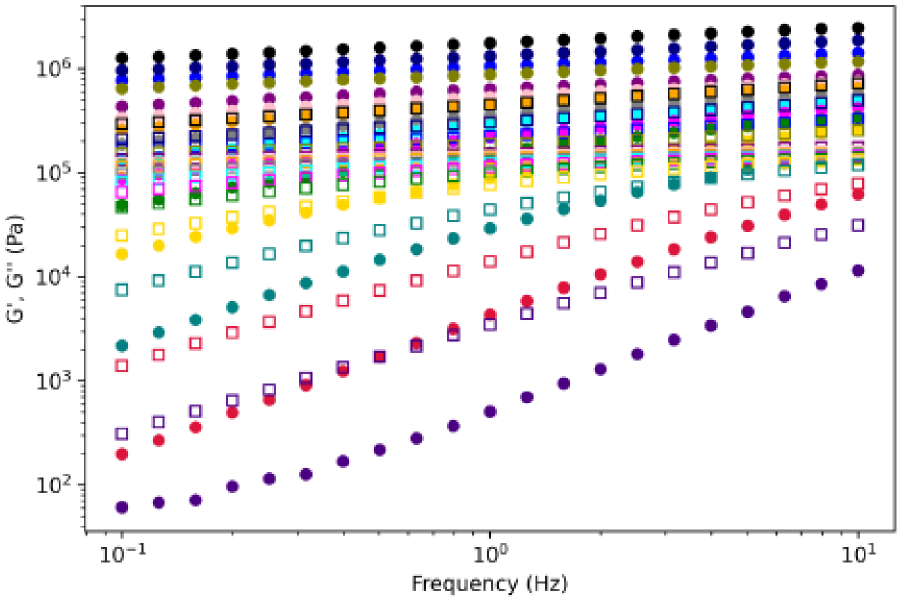

Figure S 75 Frequency sweeps of **SPE2** at 0 (black), 10 (red), 20 (blue), 30 (olive), 40 (navy), 50 (purple), 60 (pink), 70 (orange), 80 (gray), 90 (cyan), 100 (magenta), 110 (green), 120 (gold), 130 (teal), 140 (crimson), 150 °C (indigo). G' closed symbols and G'' open symbols frequency sweeps were performed between 0.1 to 10 Hz at an applied strain of 0.1%.

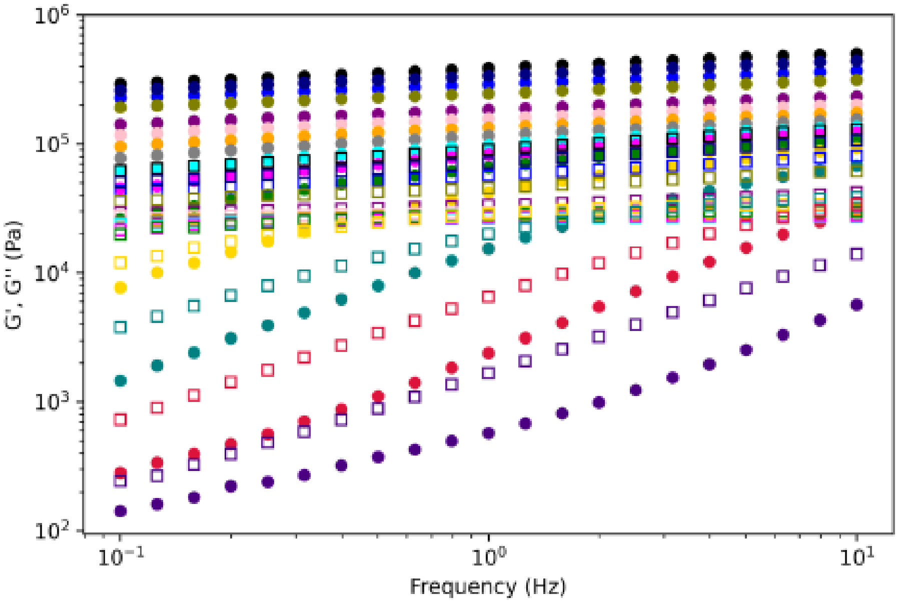

Figure S 76 Frequency sweeps of **SPE3** at 0 (black), 10 (red), 20 (blue), 30 (olive), 40 (navy), 50 (purple), 60 (pink), 70 (orange), 80 (gray), 90 (cyan), 100 (magenta), 110 (green), 120 (gold), 130 (teal), 140 (crimson), 150 °C (indigo). G' closed symbols and G'' open symbols frequency sweeps were performed between 0.1 to 10 Hz at an applied strain of 0.1%.

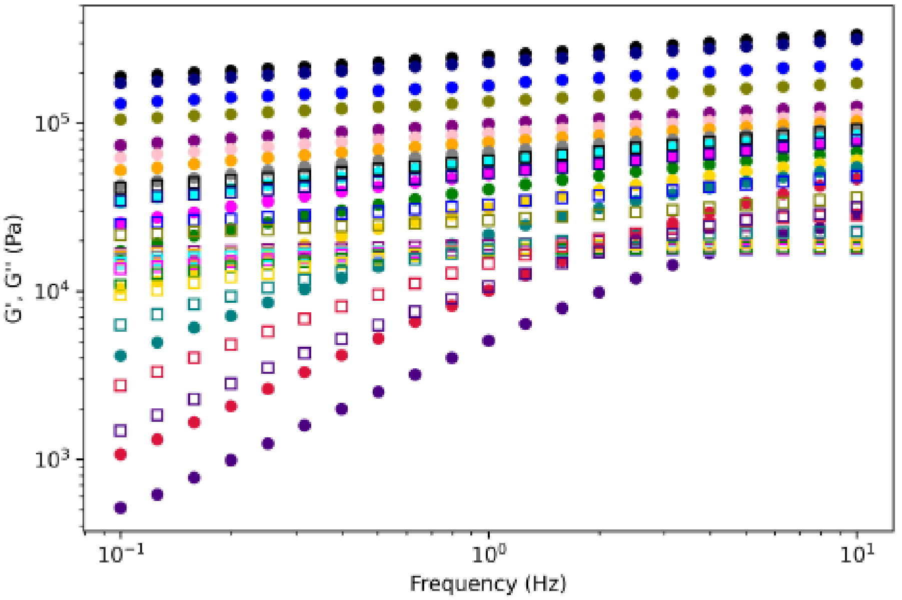

Figure S 77 Frequency sweeps of **SPE4** at 0 (black), 10 (red), 20 (blue), 30 (olive), 40 (navy), 50 (purple), 60 (pink), 70 (orange), 80 (gray), 90 (cyan), 100 (magenta), 110 (green), 120 (gold), 130 (teal), 140 (crimson), 150 °C (indigo). G' closed symbols and G'' open symbols frequency sweeps were performed between 0.1 to 10 Hz at an applied strain of 0.1%.

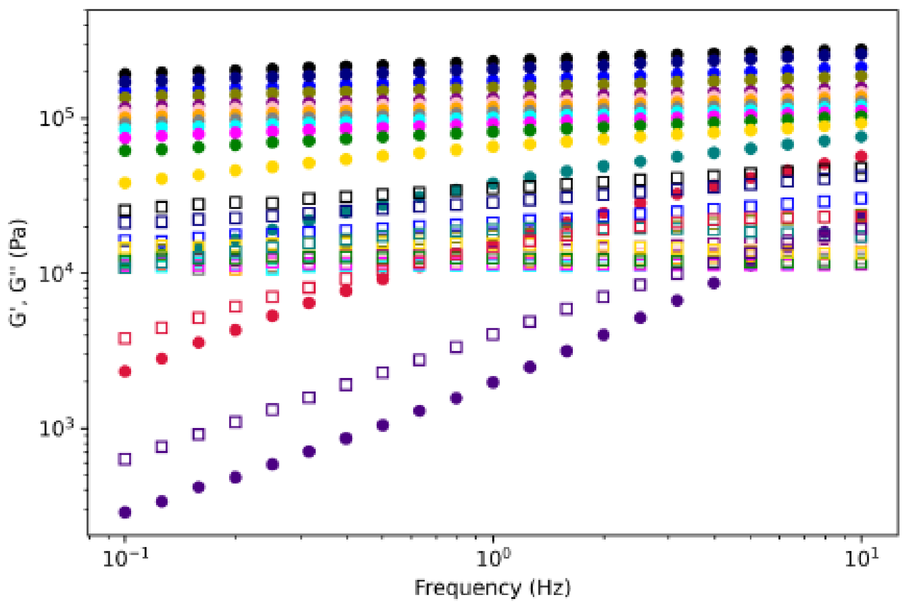

Figure S 78 Frequency sweeps of **SPE5** at 0 (black), 10 (red), 20 (blue), 30 (olive), 40 (navy), 50 (purple), 60 (pink), 70 (orange), 80 (gray), 90 (cyan), 100 (magenta), 110 (green), 120 (gold), 130 (teal), 140 (crimson), 150 °C (indigo). G' closed symbols and G'' open symbols frequency sweeps were performed between 0.1 to 10 Hz at an applied strain of 0.1%.

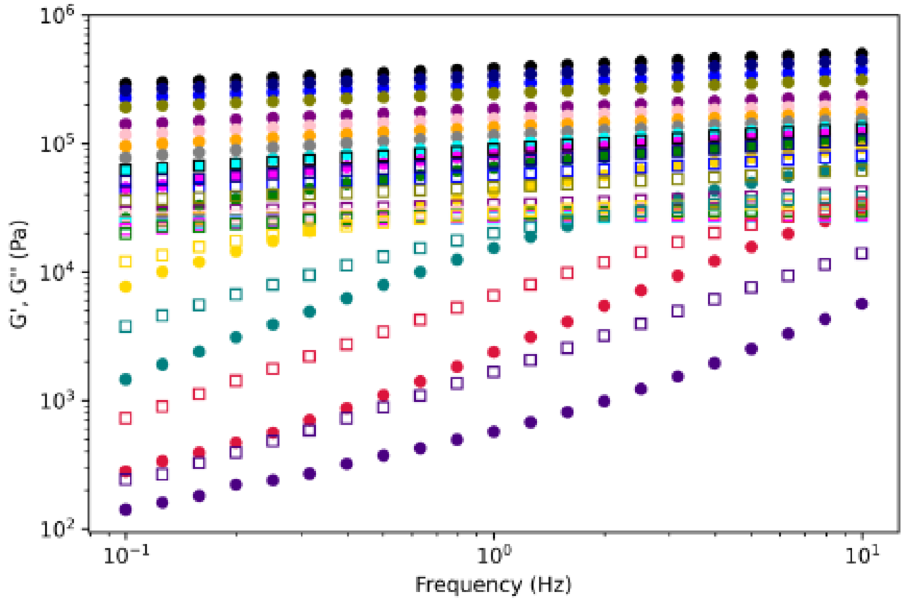

Figure S 79 Frequency sweeps of **SPE6** at 0 (black), 10 (red), 20 (blue), 30 (olive), 40 (navy), 50 (purple), 60 (pink), 70 (orange), 80 (gray), 90 (cyan), 100 (magenta), 110 (green), 120 (gold), 130 (teal), 140 (crimson), 150 °C (indigo). G' closed symbols and G'' open symbols frequency sweeps were performed between 0.1 to 10 Hz at an applied strain of 0.1%.

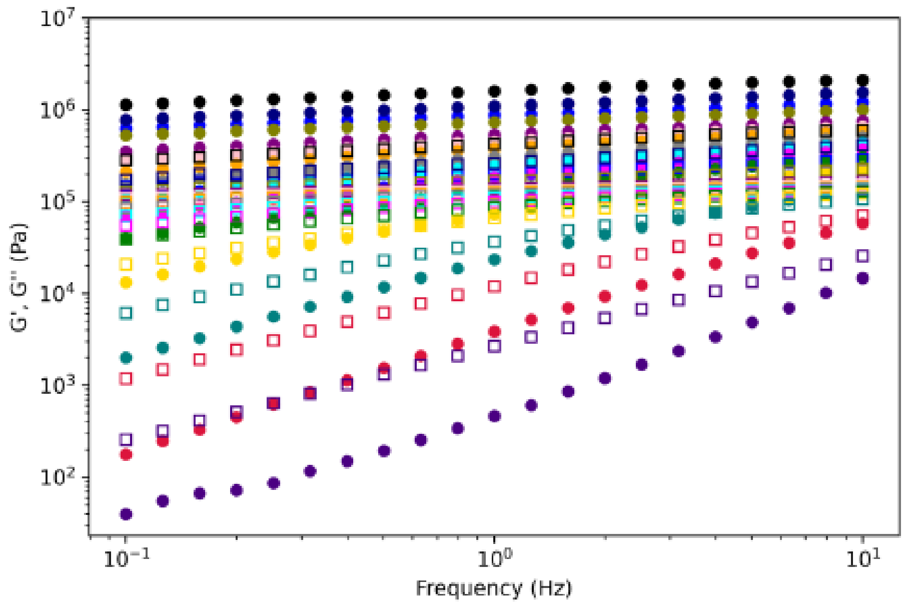

Figure S 80 Frequency sweeps of **SPE7** at 0 (black), 10 (red), 20 (blue), 30 (olive), 40 (navy), 50 (purple), 60 (pink), 70 (orange), 80 (gray), 90 (cyan), 100 (magenta), 110 (green), 120 (gold), 130 (teal), 140 (crimson), 150 °C (indigo). G' closed symbols and G'' open symbols frequency sweeps were performed between 0.1 to 10 Hz at an applied strain of 0.1%.

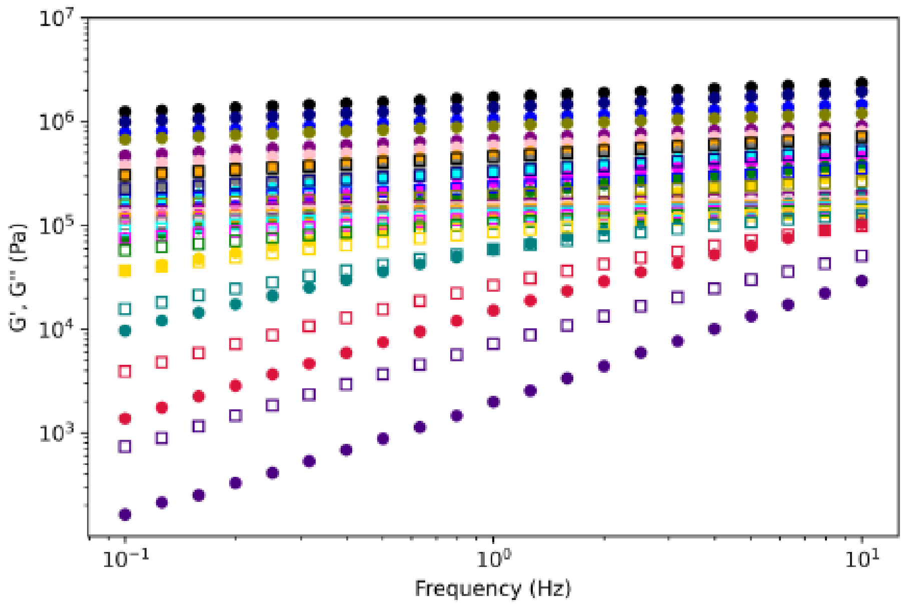

Figure S 81 Frequency sweeps of **SPE8** at 0 (black), 10 (red), 20 (blue), 30 (olive), 40 (navy), 50 (purple), 60 (pink), 70 (orange), 80 (gray), 90 (cyan), 100 (magenta), 110 (green), 120 (gold), 130 (teal), 140 (crimson), 150 °C (indigo). G' closed symbols and G'' open symbols frequency sweeps were performed between 0.1 to 10 Hz at an applied strain of 0.1%.

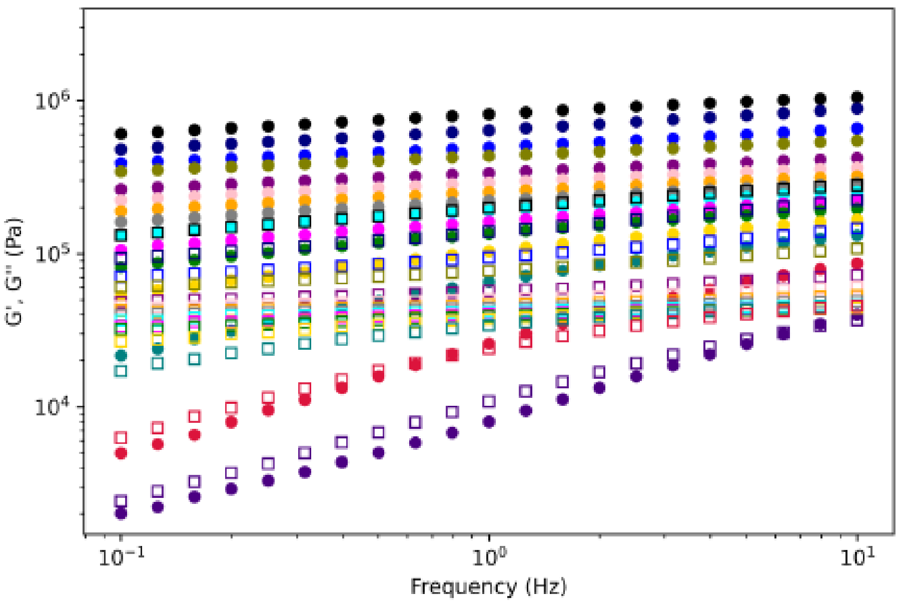

Figure S 82 Frequency sweeps of **SPE9** at 0 (black), 10 (red), 20 (blue), 30 (olive), 40 (navy), 50 (purple), 60 (pink), 70 (orange), 80 (gray), 90 (cyan), 100 (magenta), 110 (green), 120 (gold), 130 (teal), 140 (crimson), 150 °C (indigo). G' closed symbols and G'' open symbols frequency sweeps were performed between 0.1 to 10 Hz at an applied strain of 0.1%.

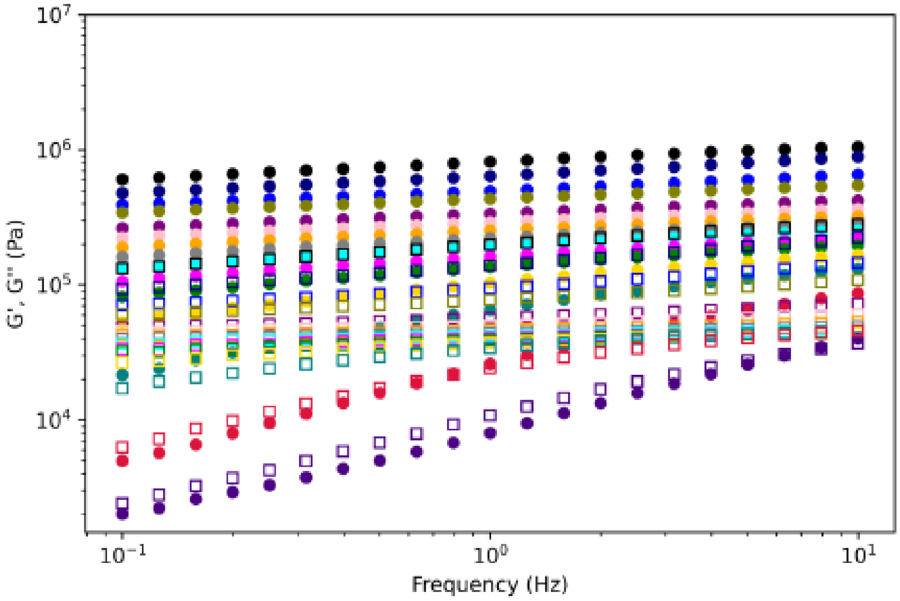

Figure S 83 Frequency sweeps of **SPE10** at 0 (black), 10 (red), 20 (blue), 30 (olive), 40 (navy), 50 (purple), 60 (pink), 70 (orange), 80 (gray), 90 (cyan), 100 (magenta), 110 (green), 120 (gold), 130 (teal), 140 (crimson), 150 °C (indigo). G' closed symbols and G'' open symbols frequency sweeps were performed between 0.1 to 10 Hz at an applied strain of 0.1%.

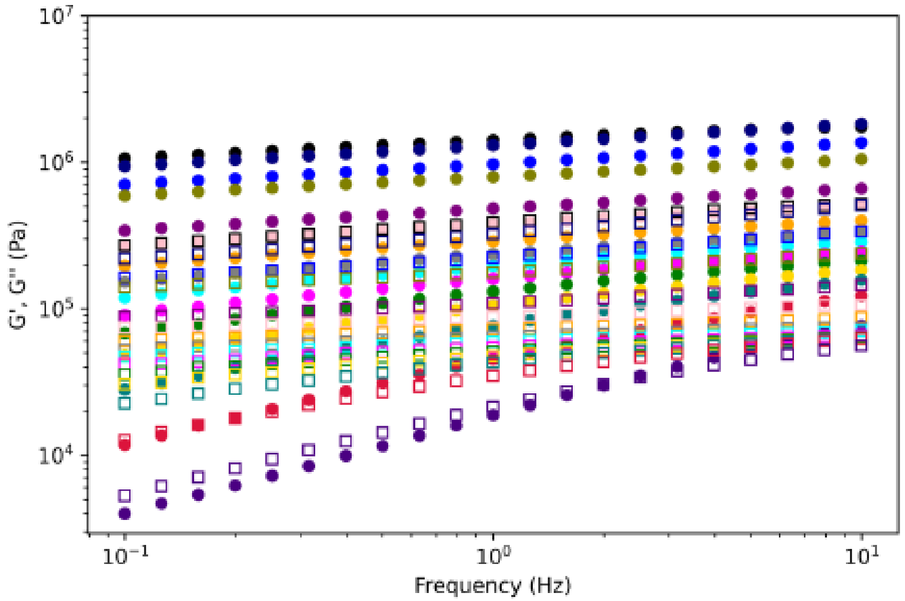

Figure S 84 Frequency sweeps of **SPE11** at 0 (black), 10 (red), 20 (blue), 30 (olive), 40 (navy), 50 (purple), 60 (pink), 70 (orange), 80 (gray), 90 (cyan), 100 (magenta), 110 (green), 120 (gold), 130 (teal), 140 (crimson), 150 °C (indigo). G' closed symbols and G'' open symbols frequency sweeps were performed between 0.1 to 10 Hz at an applied strain of 0.1%.

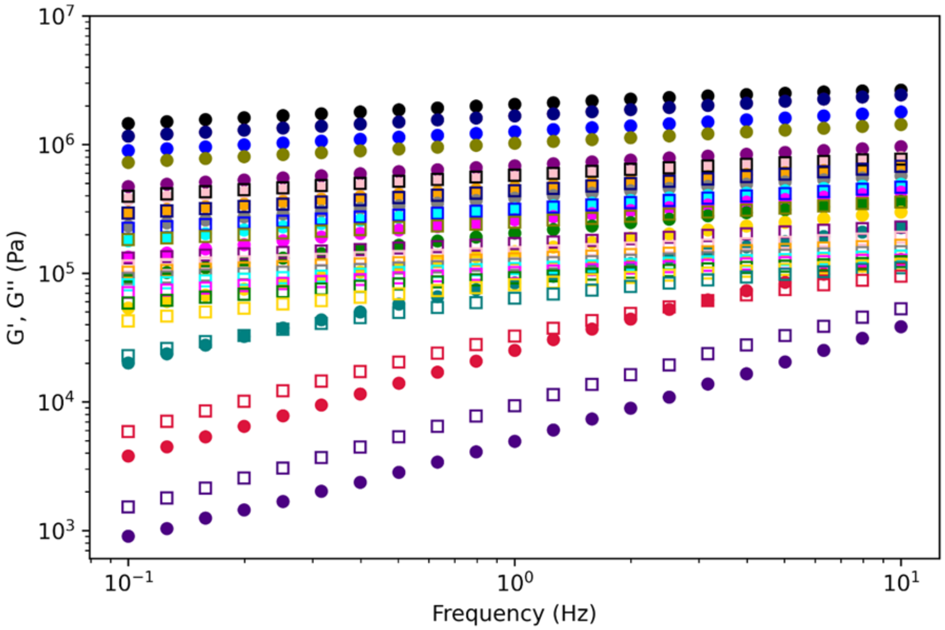

Figure S 85 Frequency sweeps of **SPE12** at 0 (black), 10 (red), 20 (blue), 30 (olive), 40 (navy), 50 (purple), 60 (pink), 70 (orange), 80 (gray), 90 (cyan), 100 (magenta), 110 (green), 120 (gold), 130 (teal), 140 (crimson), 150 °C (indigo). G' closed symbols and G'' open symbols frequency sweeps were performed between 0.1 to 10 Hz at an applied strain of 0.1%.

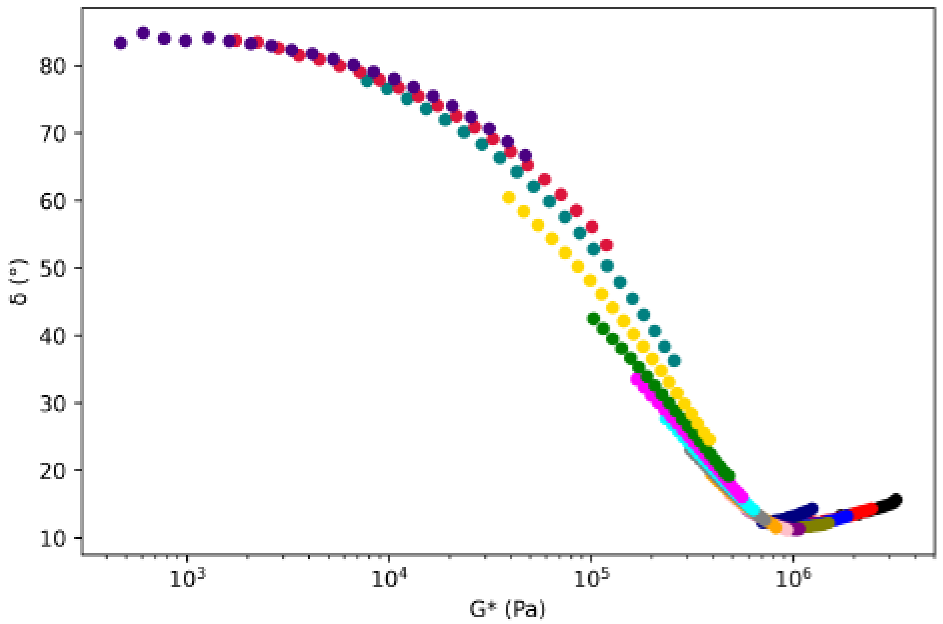

Figure S 86 van Gurp-Palmen plot of **SPE1** at 0 (black), 10 (red), 20 (blue), 30 (olive), 40 (navy), 50 (purple), 60 (pink), 70 (orange), 80 (gray), 90 (cyan), 100 (magenta), 110 (green), 120 (gold), 130 (teal), 140 (crimson), 150 °C (indigo). Frequency sweeps were performed between 0.1 to 10 Hz at an applied strain of 0.1%.

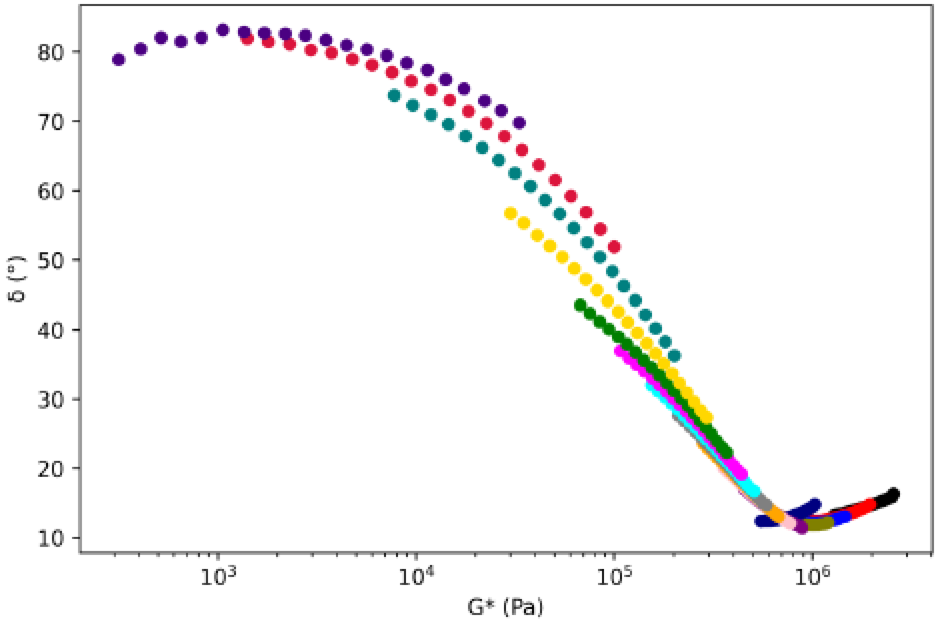

Figure S 87 van Gurp-Palmen plot of **SPE2** at 0 (black), 10 (red), 20 (blue), 30 (olive), 40 (navy), 50 (purple), 60 (pink), 70 (orange), 80 (gray), 90 (cyan), 100 (magenta), 110 (green), 120 (gold), 130 (teal), 140 (crimson), 150 °C (indigo). Frequency sweeps were performed between 0.1 to 10 Hz at an applied strain of 0.1%.

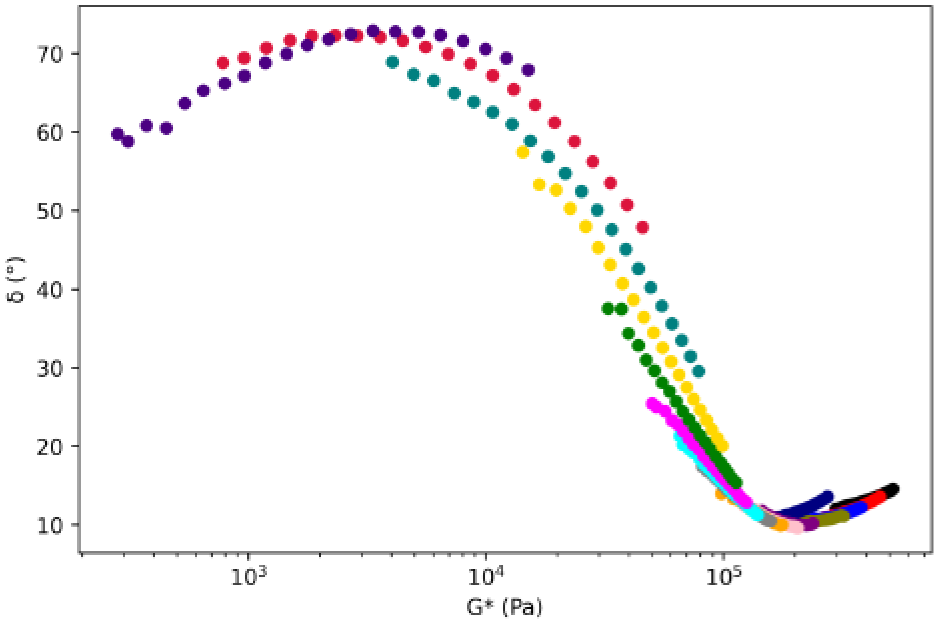

Figure S 88 van Gurp-Palmen plot of **SPE3** at 0 (black), 10 (red), 20 (blue), 30 (olive), 40 (navy), 50 (purple), 60 (pink), 70 (orange), 80 (gray), 90 (cyan), 100 (magenta), 110 (green), 120 (gold), 130 (teal), 140 (crimson), 150 °C (indigo). Frequency sweeps were performed between 0.1 to 10 Hz at an applied strain of 0.1%.

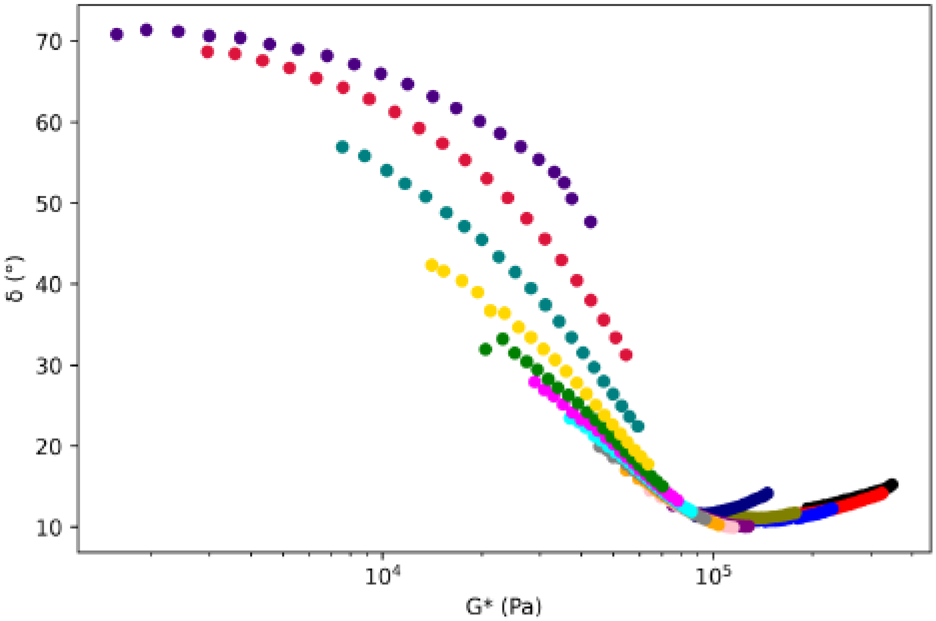

Figure S 89 van Gurp-Palmen plot of **SPE4** at 0 (black), 10 (red), 20 (blue), 30 (olive), 40 (navy), 50 (purple), 60 (pink), 70 (orange), 80 (gray), 90 (cyan), 100 (magenta), 110 (green), 120 (gold), 130 (teal), 140 (crimson), 150 °C (indigo). Frequency sweeps were performed between 0.1 to 10 Hz at an applied strain of 0.1%.

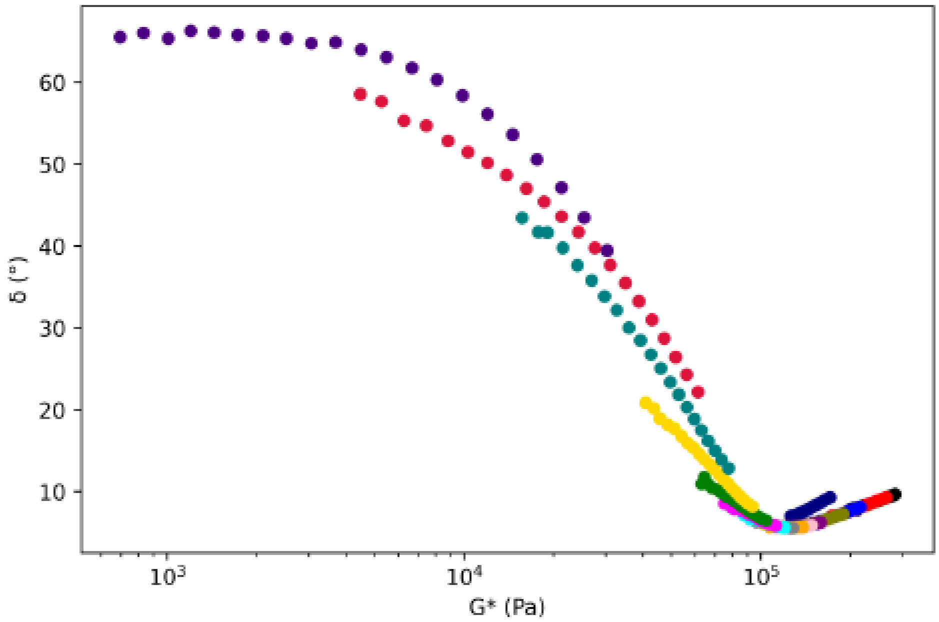

Figure S 90 van Gurp-Palmen plot of **SPE5** at 0 (black), 10 (red), 20 (blue), 30 (olive), 40 (navy), 50 (purple), 60 (pink), 70 (orange), 80 (gray), 90 (cyan), 100 (magenta), 110 (green), 120 (gold), 130 (teal), 140 (crimson), 150 °C (indigo). Frequency sweeps were performed between 0.1 to 10 Hz at an applied strain of 0.1%.

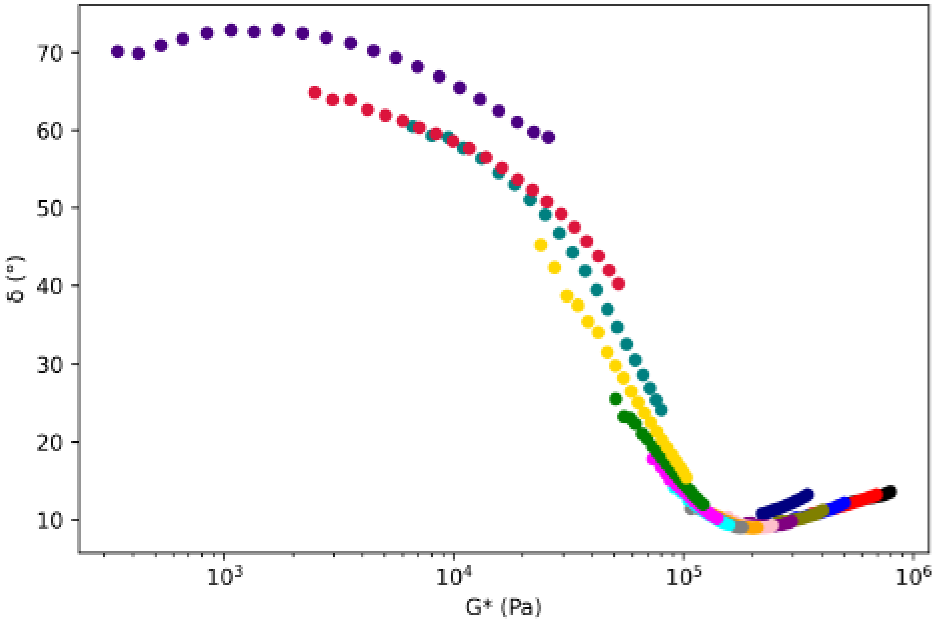

Figure S 91 van Gurp-Palmen plot of **SPE6** at 0 (black), 10 (red), 20 (blue), 30 (olive), 40 (navy), 50 (purple), 60 (pink), 70 (orange), 80 (gray), 90 (cyan), 100 (magenta), 110 (green), 120 (gold), 130 (teal), 140 (crimson), 150 °C (indigo). Frequency sweeps were performed between 0.1 to 10 Hz at an applied strain of 0.1%.

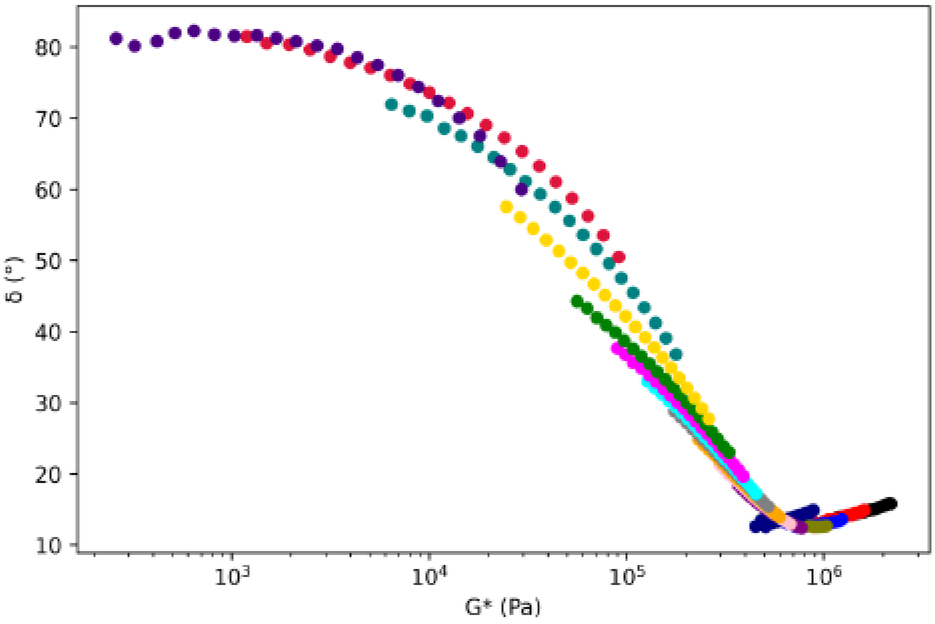

Figure S 92 van Gurp-Palmen plot of **SPE7** at 0 (black), 10 (red), 20 (blue), 30 (olive), 40 (navy), 50 (purple), 60 (pink), 70 (orange), 80 (gray), 90 (cyan), 100 (magenta), 110 (green), 120 (gold), 130 (teal), 140 (crimson), 150 °C (indigo). Frequency sweeps were performed between 0.1 to 10 Hz at an applied strain of 0.1%.

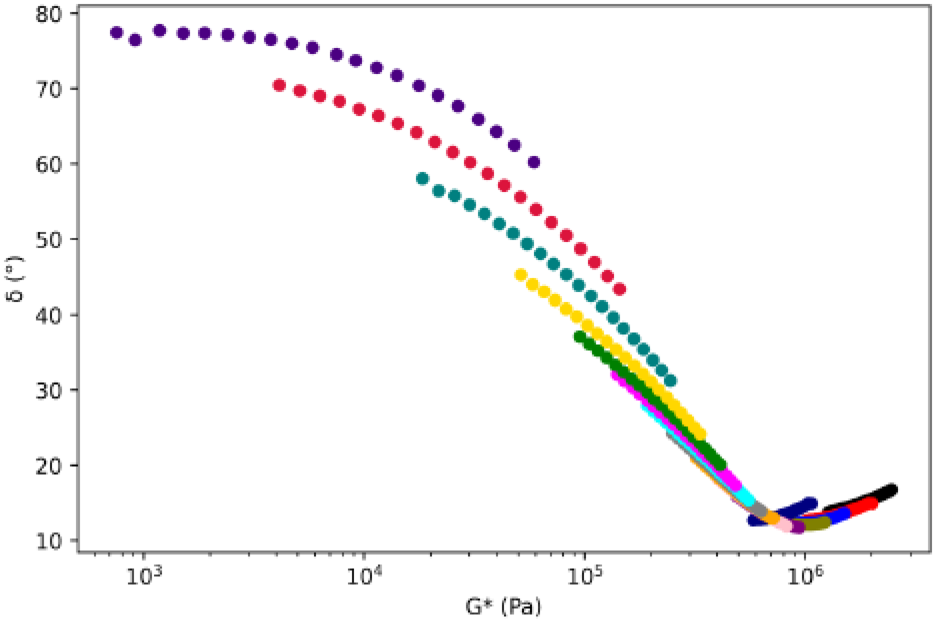

Figure S 93 van Gurp-Palmen plot of **SPE8** at 0 (black), 10 (red), 20 (blue), 30 (olive), 40 (navy), 50 (purple), 60 (pink), 70 (orange), 80 (gray), 90 (cyan), 100 (magenta), 110 (green), 120 (gold), 130 (teal), 140 (crimson), 150 °C (indigo). Frequency sweeps were performed between 0.1 to 10 Hz at an applied strain of 0.1%.

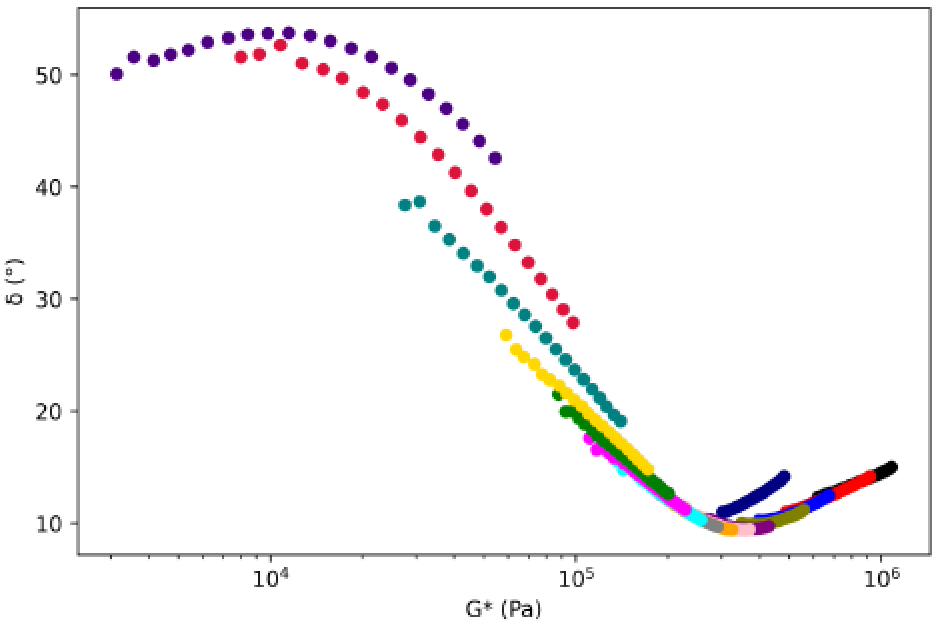

Figure S 94 van Gurp-Palmen plot of **SPE9** at 0 (black), 10 (red), 20 (blue), 30 (olive), 40 (navy), 50 (purple), 60 (pink), 70 (orange), 80 (gray), 90 (cyan), 100 (magenta), 110 (green), 120 (gold), 130 (teal), 140 (crimson), 150 °C (indigo). Frequency sweeps were performed between 0.1 to 10 Hz at an applied strain of 0.1%.

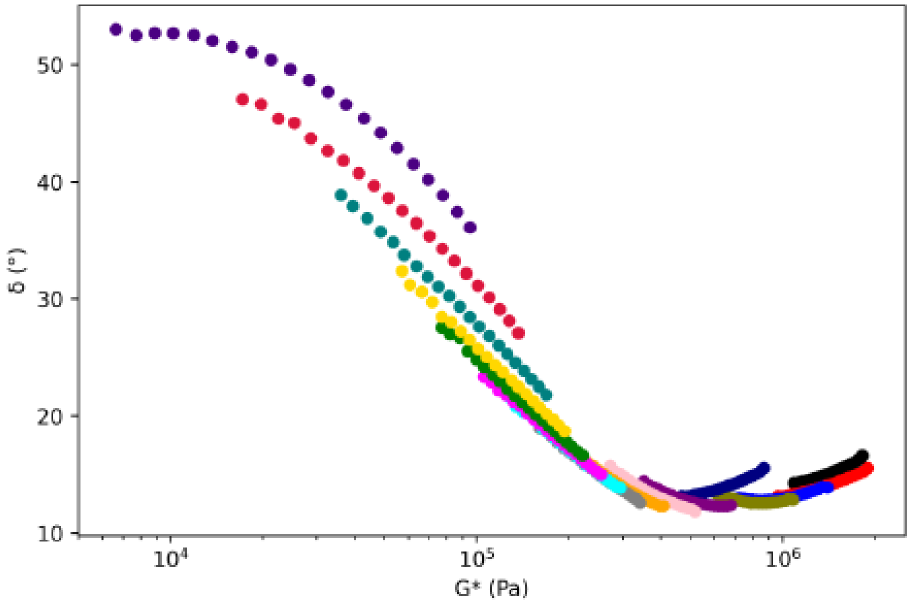

Figure S 95 van Gurp-Palmen plot of **SPE10** at 0 (black), 10 (red), 20 (blue), 30 (olive), 40 (navy), 50 (purple), 60 (pink), 70 (orange), 80 (gray), 90 (cyan), 100 (magenta), 110 (green), 120 (gold), 130 (teal), 140 (crimson), 150 °C (indigo). Frequency sweeps were performed between 0.1 to 10 Hz at an applied strain of 0.1%.

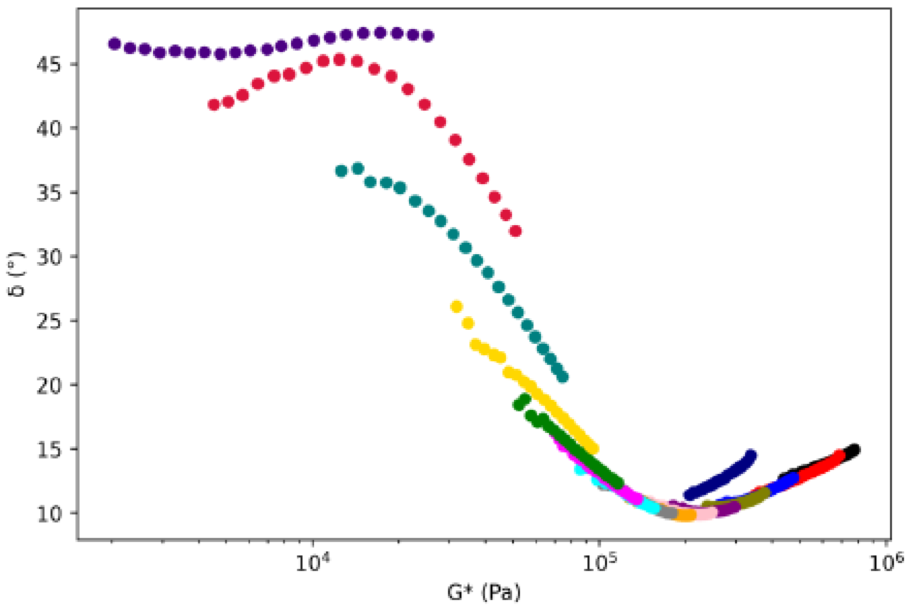

Figure S 96 van Gurp-Palmen plot of **SPE11** at 0 (black), 10 (red), 20 (blue), 30 (olive), 40 (navy), 50 (purple), 60 (pink), 70 (orange), 80 (gray), 90 (cyan), 100 (magenta), 110 (green), 120 (gold), 130 (teal), 140 (crimson), 150 °C (indigo). Frequency sweeps were performed between 0.1 to 10 Hz at an applied strain of 0.1%.

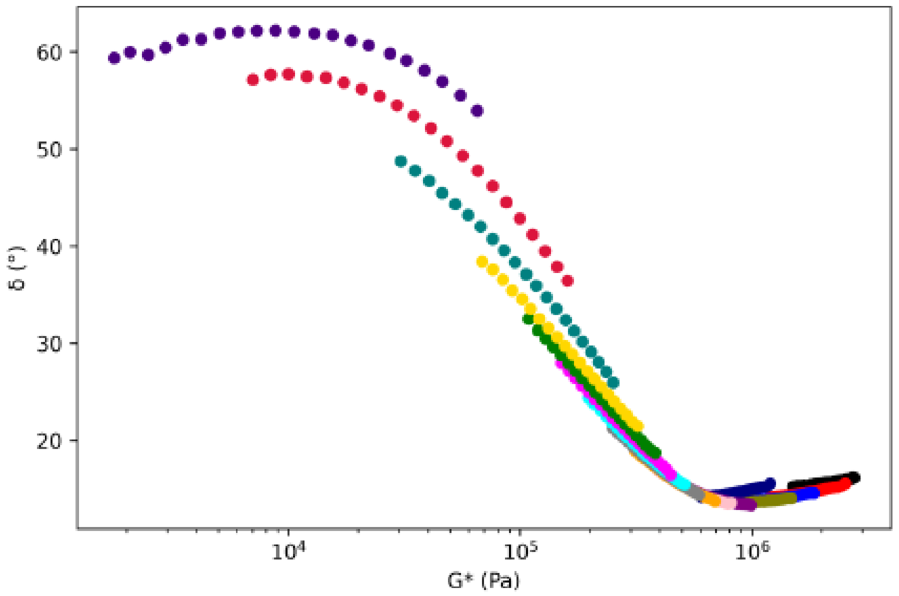

Figure S 97 van Gurp-Palmen plot of **SPE12** at 0 (black), 10 (red), 20 (blue), 30 (olive), 40 (navy), 50 (purple), 60 (pink), 70 (orange), 80 (gray), 90 (cyan), 100 (magenta), 110 (green), 120 (gold), 130 (teal), 140 (crimson), 150 °C (indigo). Frequency sweeps were performed between 0.1 to 10 Hz at an applied strain of 0.1%.

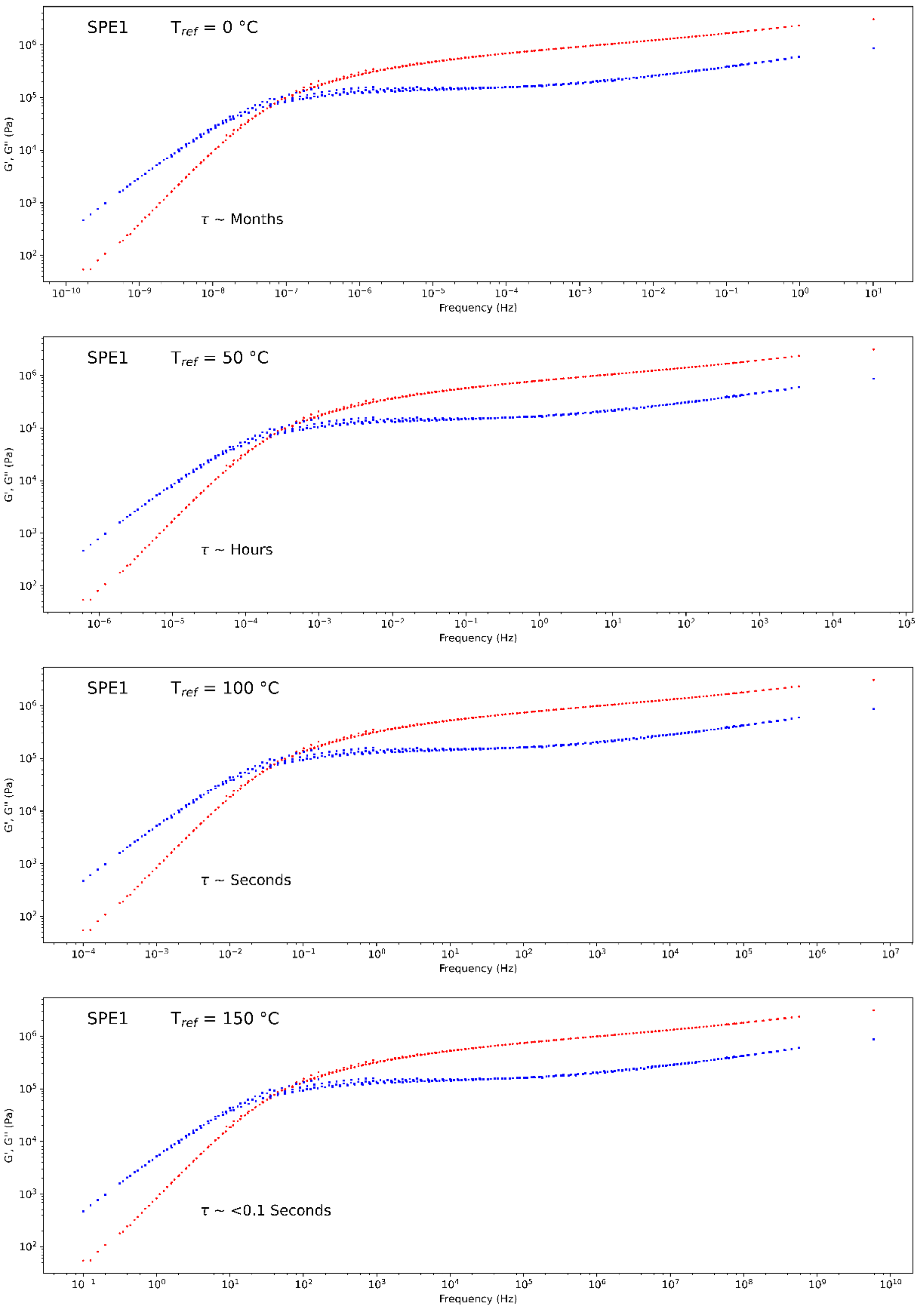

Figure S 98 Master curves of **SPE1** at a reference temperature (T_{ref}) of 0, 50, 100, and 150 °C, prepared by the TTS for G' and G'' values obtained from frequency sweeps at 10 °C intervals from 0 to 150 °C, frequency sweeps were performed between 0.1 to 10 Hz at an applied strain of 0.1%.

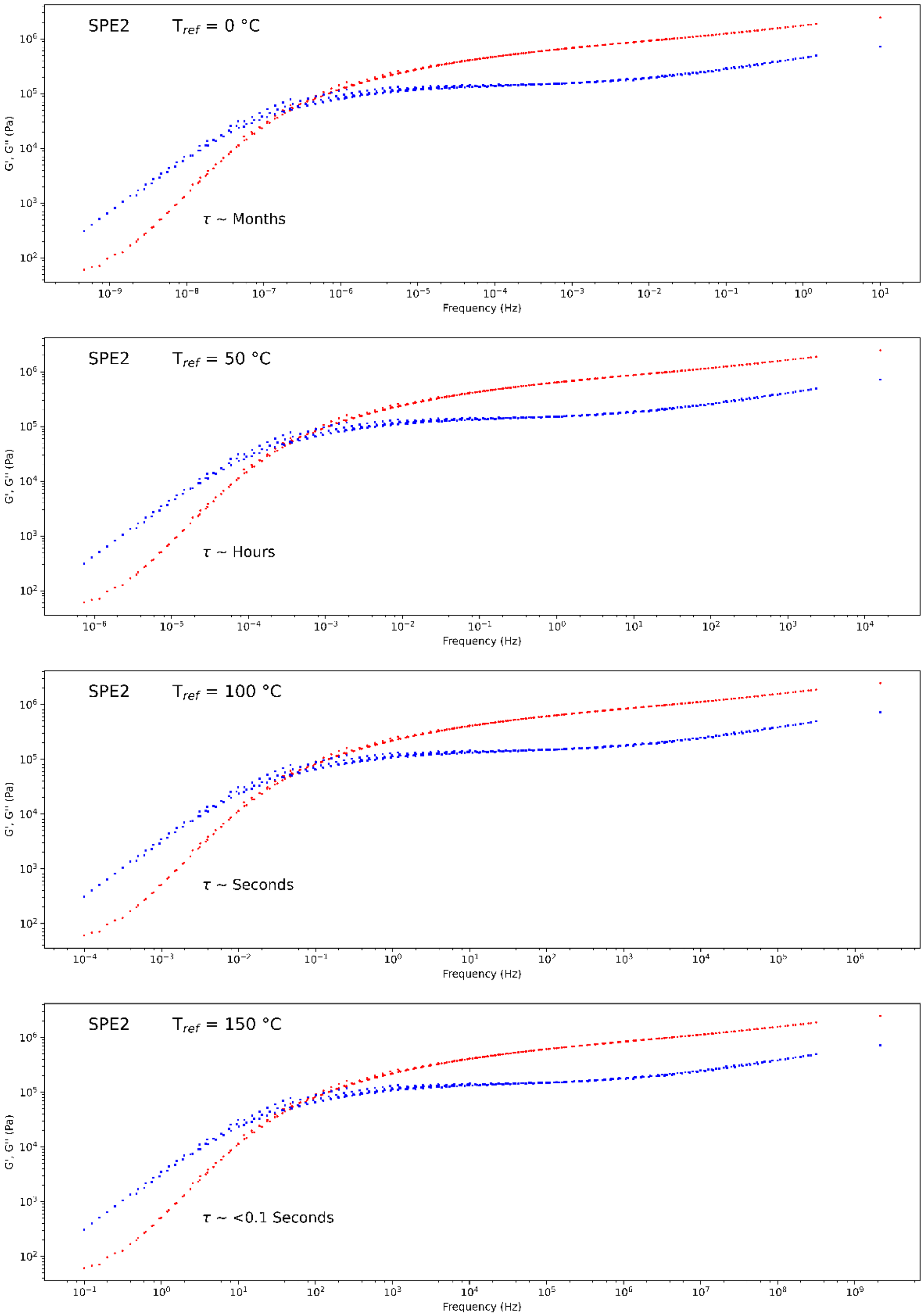

Figure S 99 Master curves of **SPE2** at a reference temperature (T_{ref}) of 0, 50, 100, and 150 °C, prepared by the TTS for G' and G'' values obtained from frequency sweeps at 10 °C intervals from 0 to 150 °C, frequency sweeps were performed between 0.1 to 10 Hz at an applied strain of 0.1%.

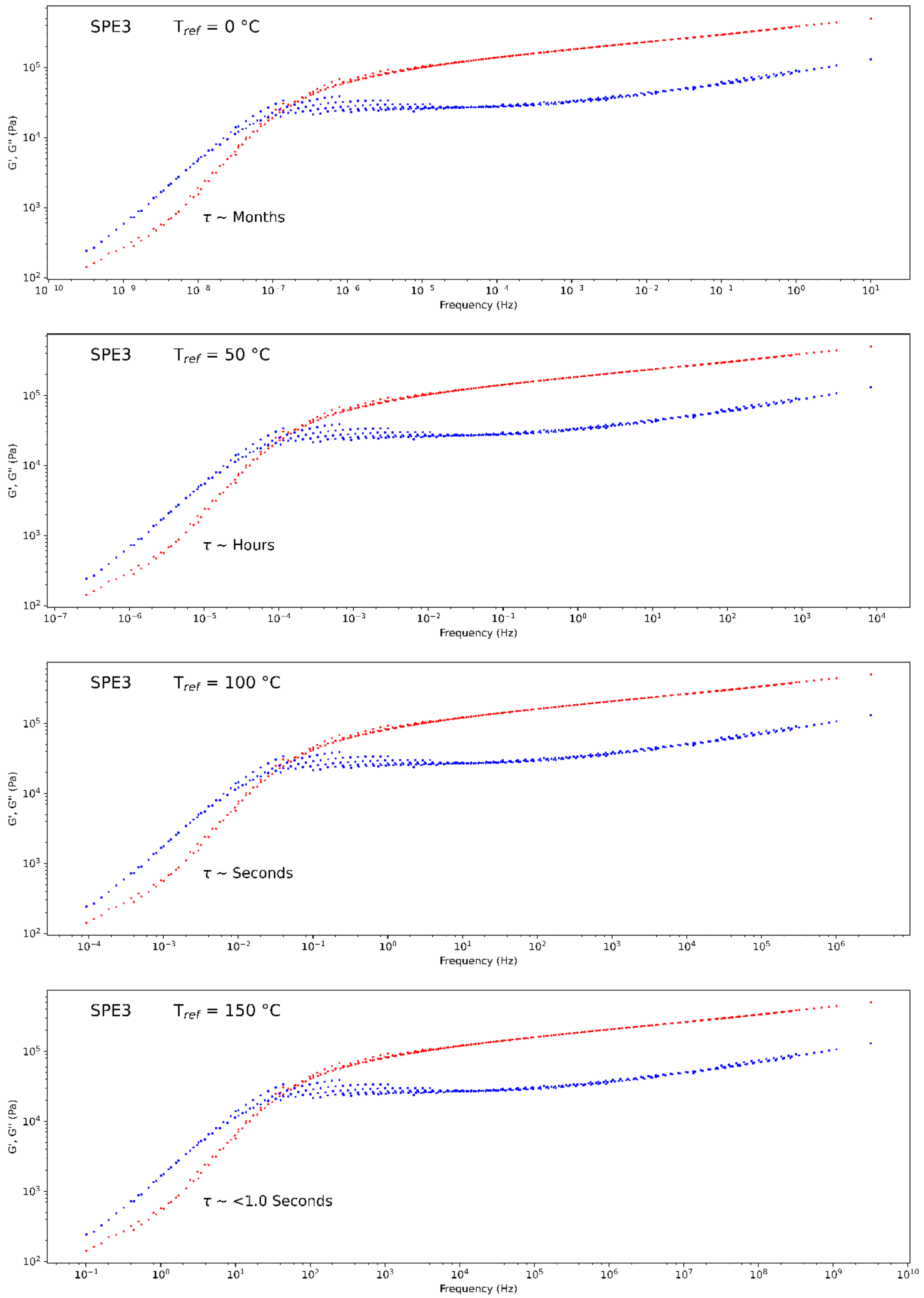

Figure S 100 Master curves of **SPE3** at a reference temperature (T_{ref}) of 0, 50, 100, and 150 °C, prepared by the TTS for G' and G'' values obtained from frequency sweeps at 10 °C intervals from 0 to 150 °C, frequency sweeps were performed between 0.1 to 10 Hz at an applied strain of 0.1%.

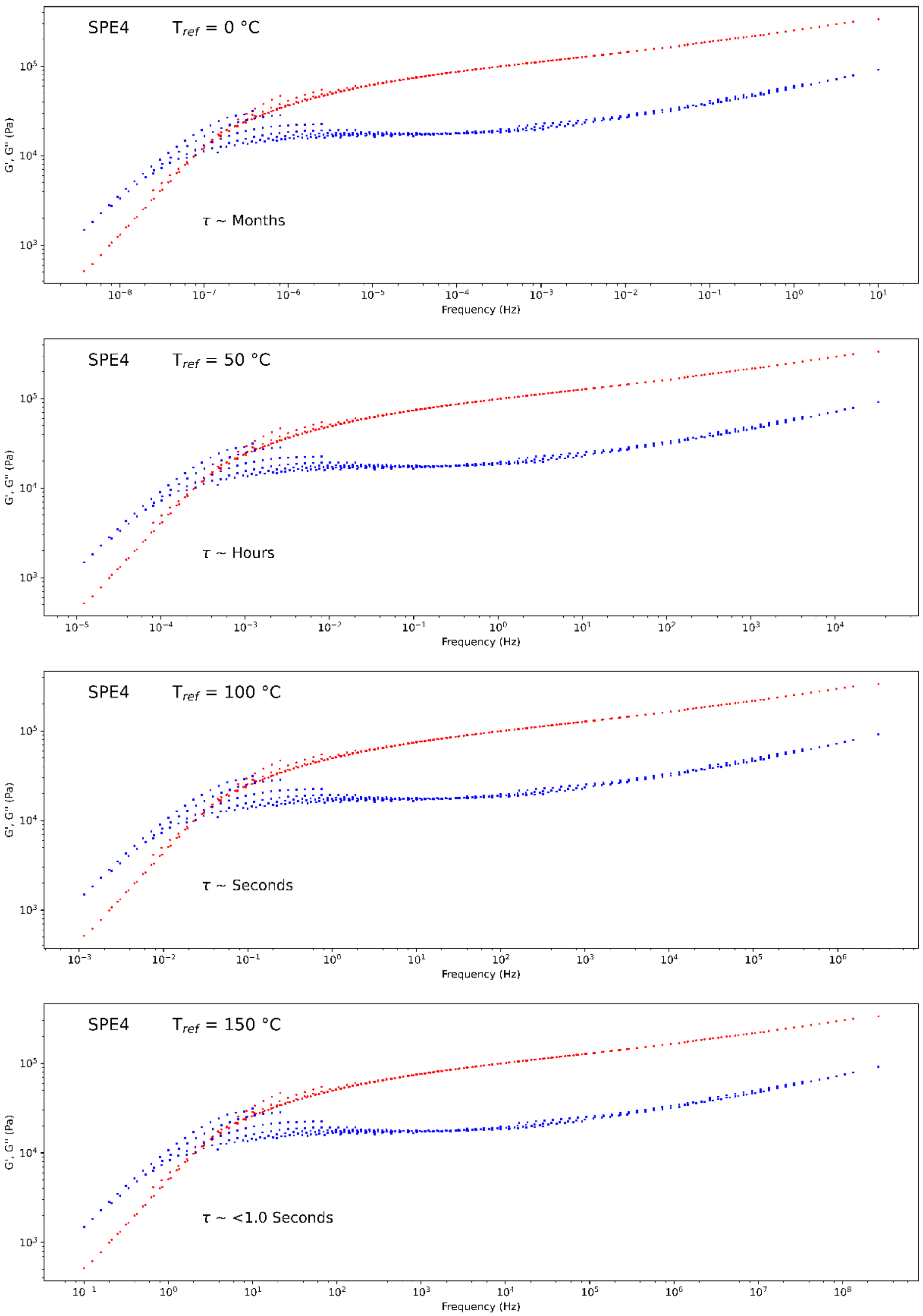

Figure S 101 Master curves of **SPE4** at a reference temperature (T_{ref}) of 0, 50, 100, and 150 °C, prepared by the TTS for G' and G'' values obtained from frequency sweeps at 10 °C intervals from 0 to 150 °C, frequency sweeps were performed between 0.1 to 10 Hz at an applied strain of 0.1%.

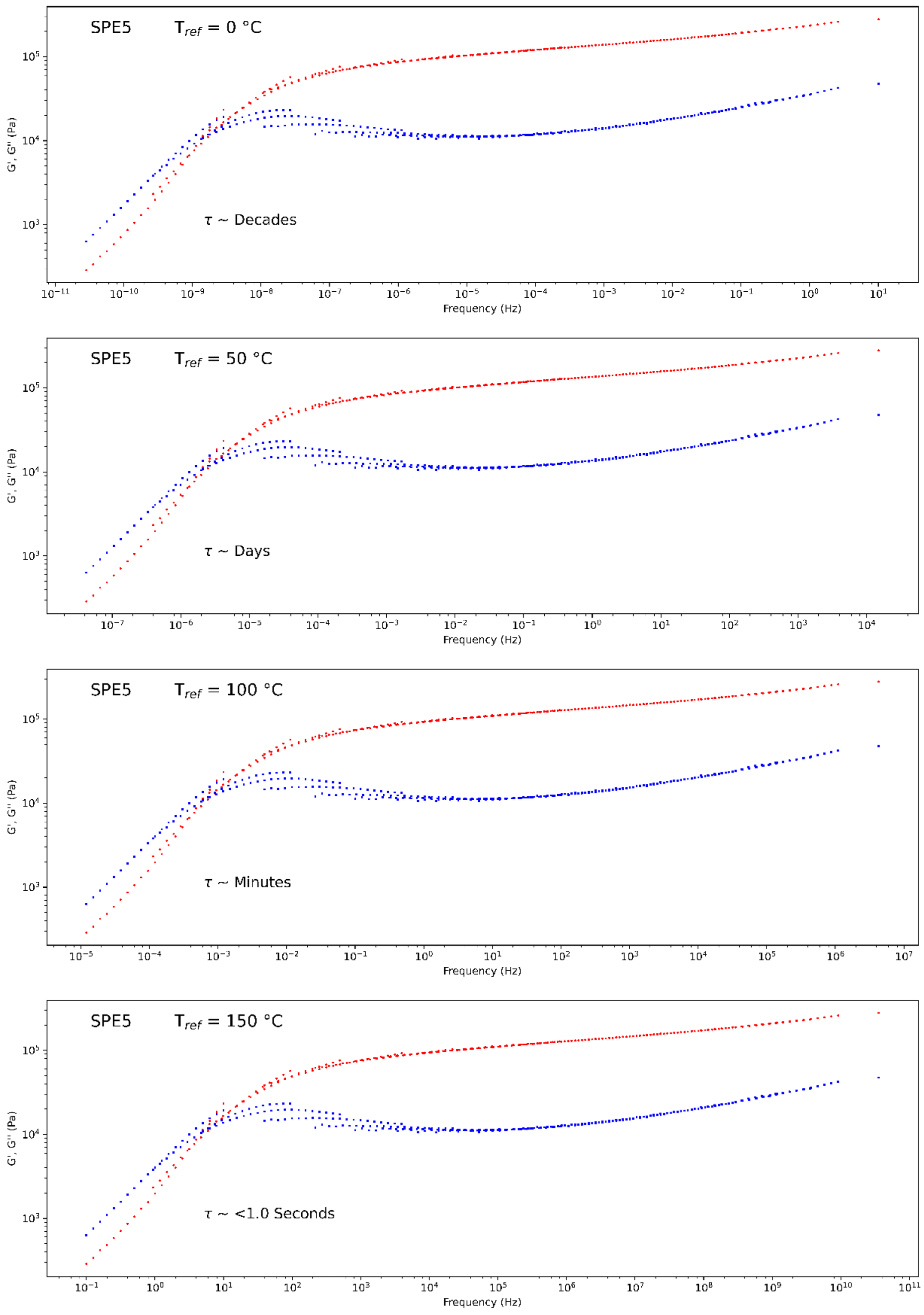

Figure S 102 Master curves of **SPE5** at a reference temperature (T_{ref}) of 0, 50, 100, and 150 °C, prepared by the TTS for G' and G'' values obtained from frequency sweeps at 10 °C intervals from 0 to 150 °C, frequency sweeps were performed between 0.1 to 10 Hz at an applied strain of 0.1%.

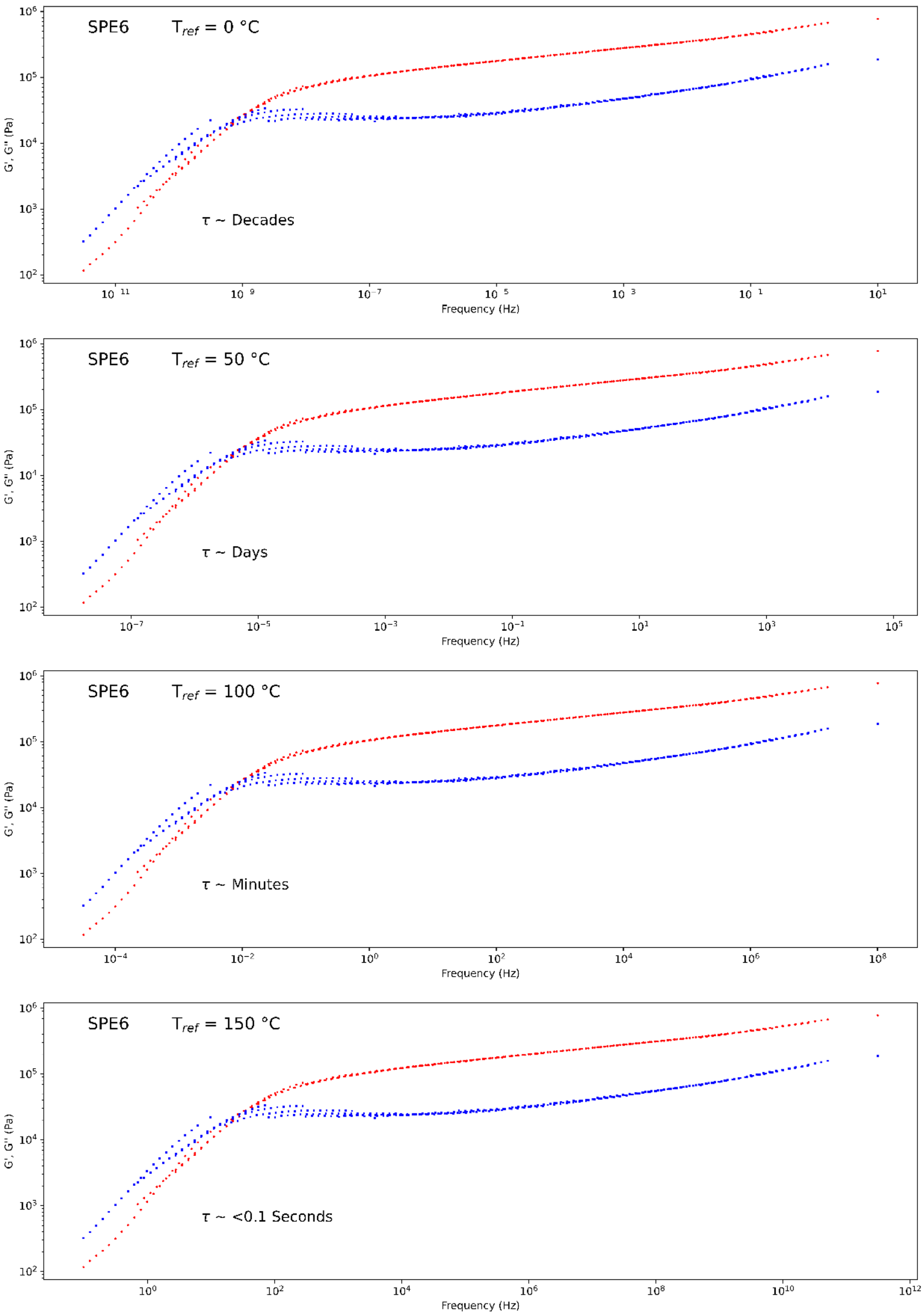

Figure S 103 Master curves of **SPE6** at a reference temperature (T_{ref}) of 0, 50, 100, and 150 °C, prepared by the TTS for G' and G'' values obtained from frequency sweeps at 10 °C intervals from 0 to 150 °C, frequency sweeps were performed between 0.1 to 10 Hz at an applied strain of 0.1%.

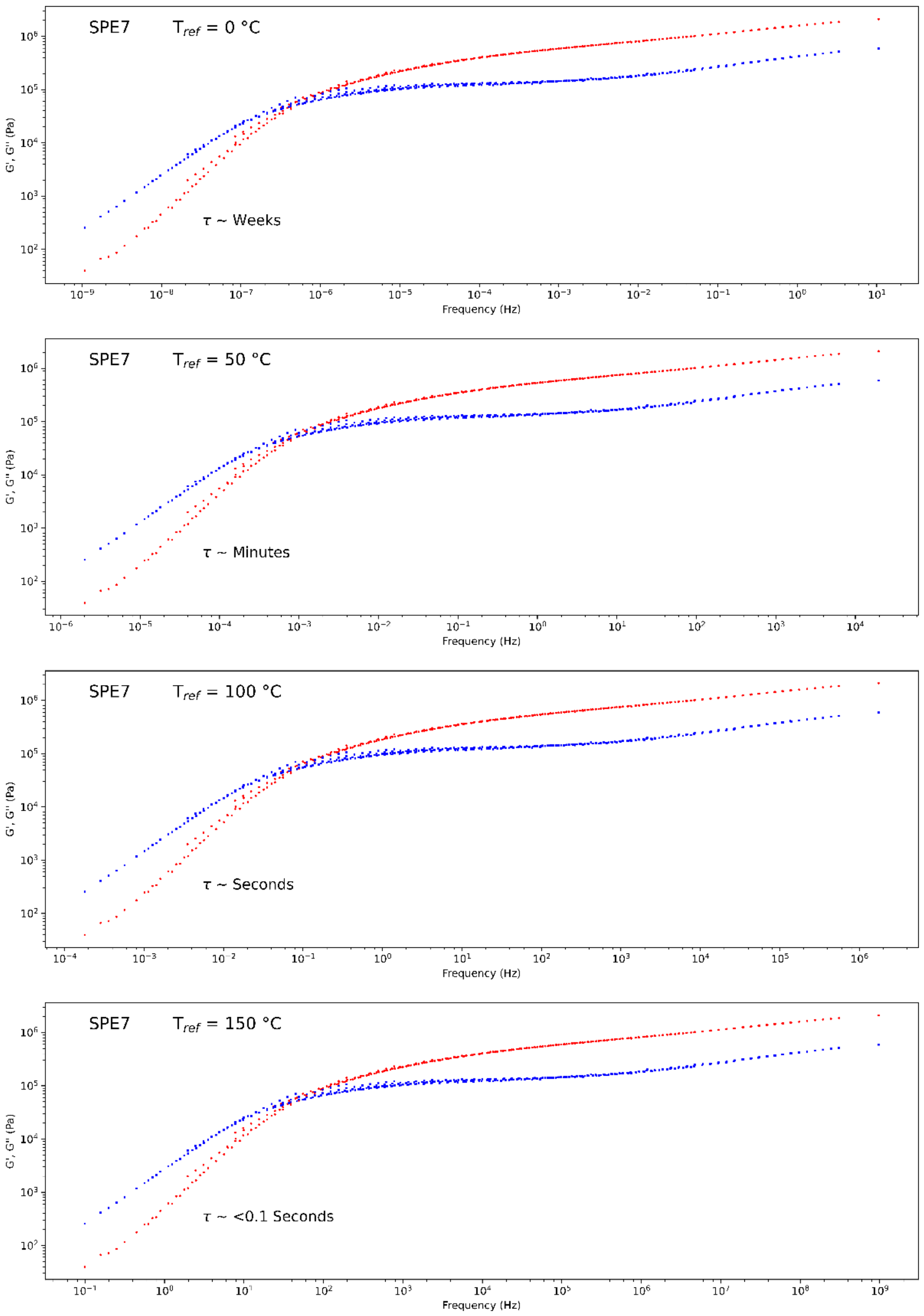

Figure S 104 Master curves of **SPE7** at a reference temperature (Tref) of 0, 50, 100, 150 °C, prepared by the TTS for G’ and G’’ values obtained at from frequency sweeps at 10 °C intervals from 0 to 150 °C, frequency sweeps were performed between 0.1 to 10 Hz at an applied strain of 0.1%.

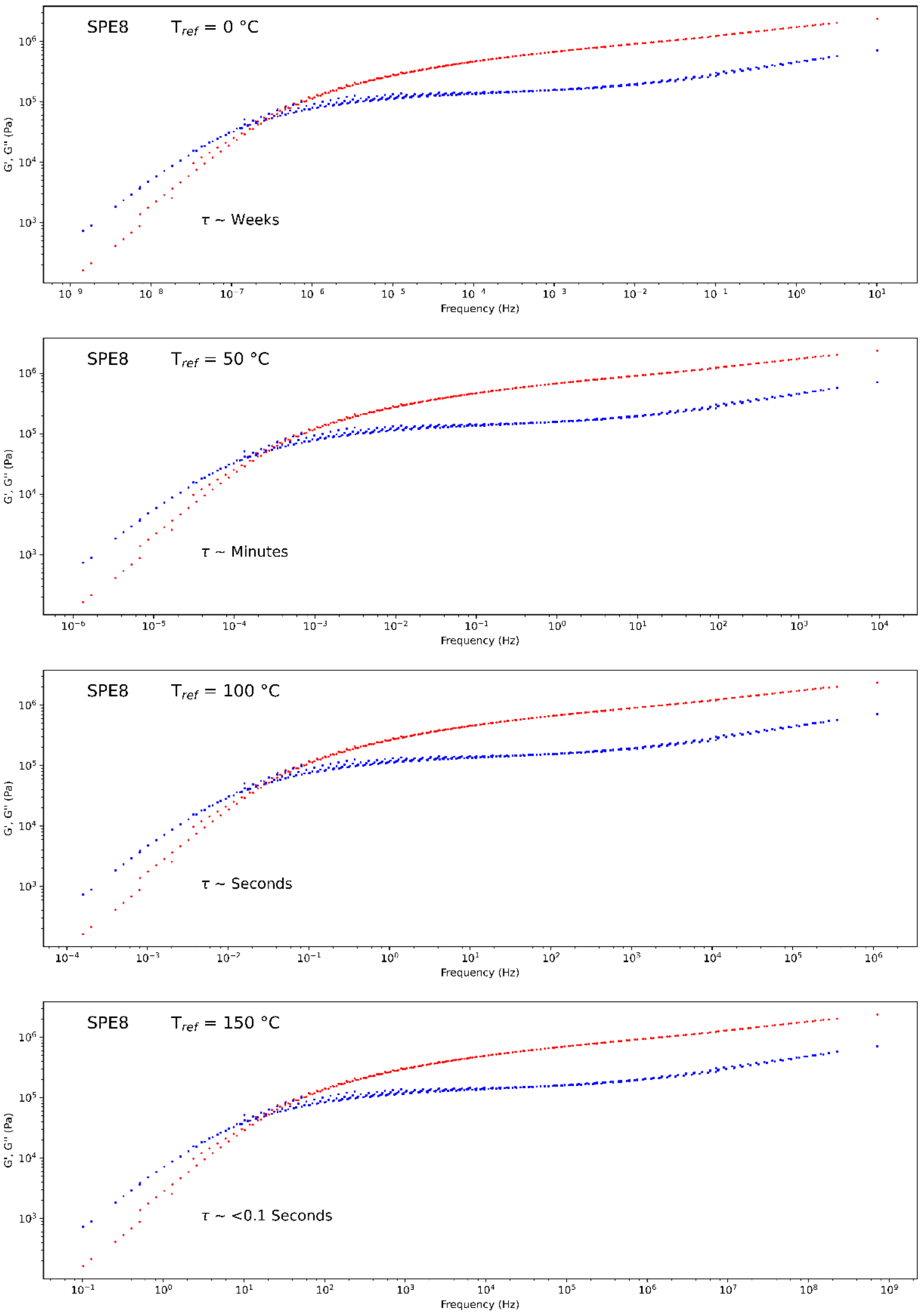

Figure S 105 Master curves of **SPE8** at a reference temperature (Tref) of 0, 50, 100, 150 °C, prepared by the TTS for G' and G'' values obtained at from frequency sweeps at 10 °C intervals from 0 to 150 °C, frequency sweeps were performed between 0.1 to 10 Hz at an applied strain of 0.1%.

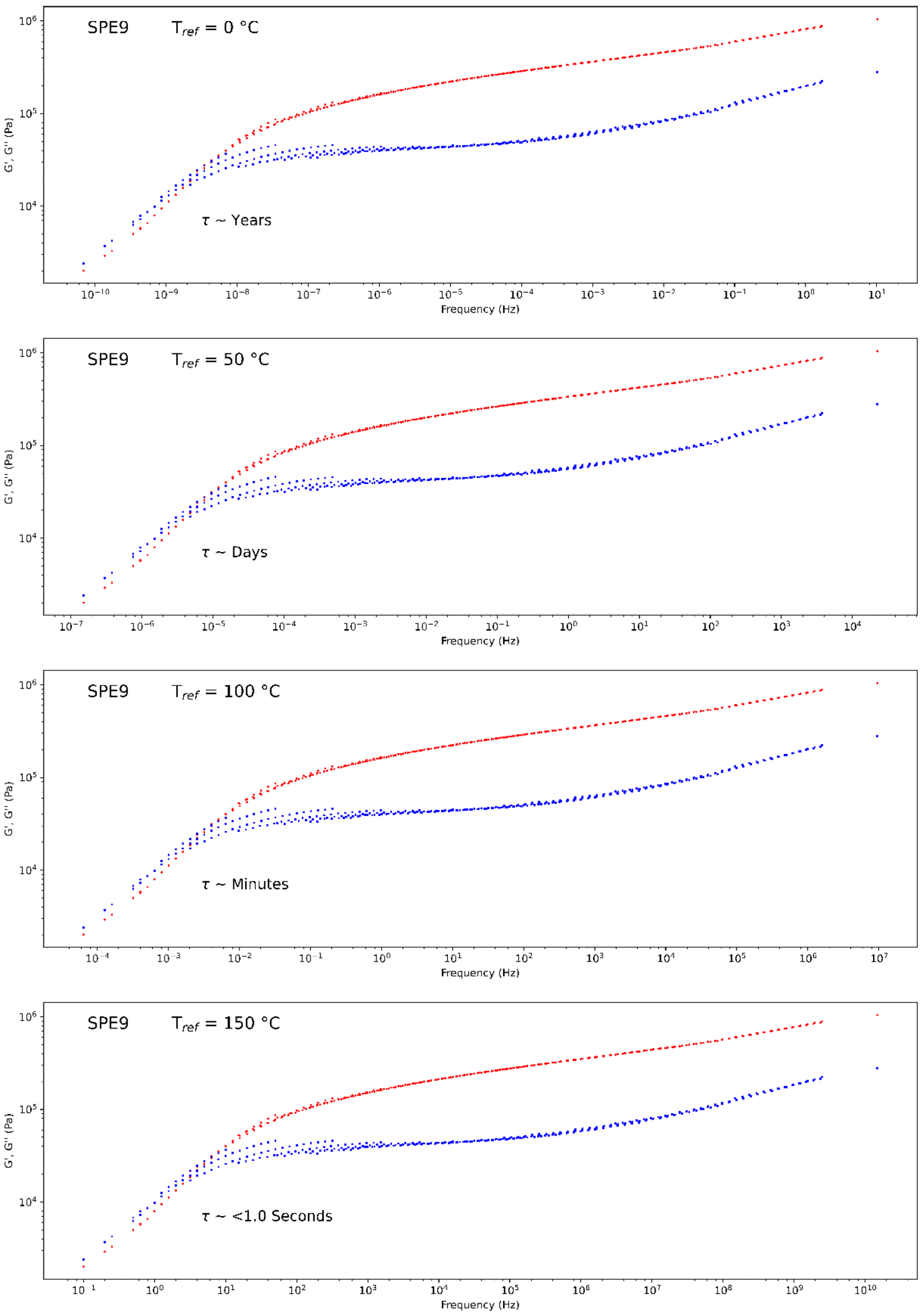

Figure S 106 Master curves of **SPE9** at a reference temperature (Tref) of 0, 50, 100, 150 °C, prepared by the TTS for G' and G'' values obtained at from frequency sweeps at 10 °C intervals from 0 to 150 °C, frequency sweeps were performed between 0.1 to 10 Hz at an applied strain of 0.1%.

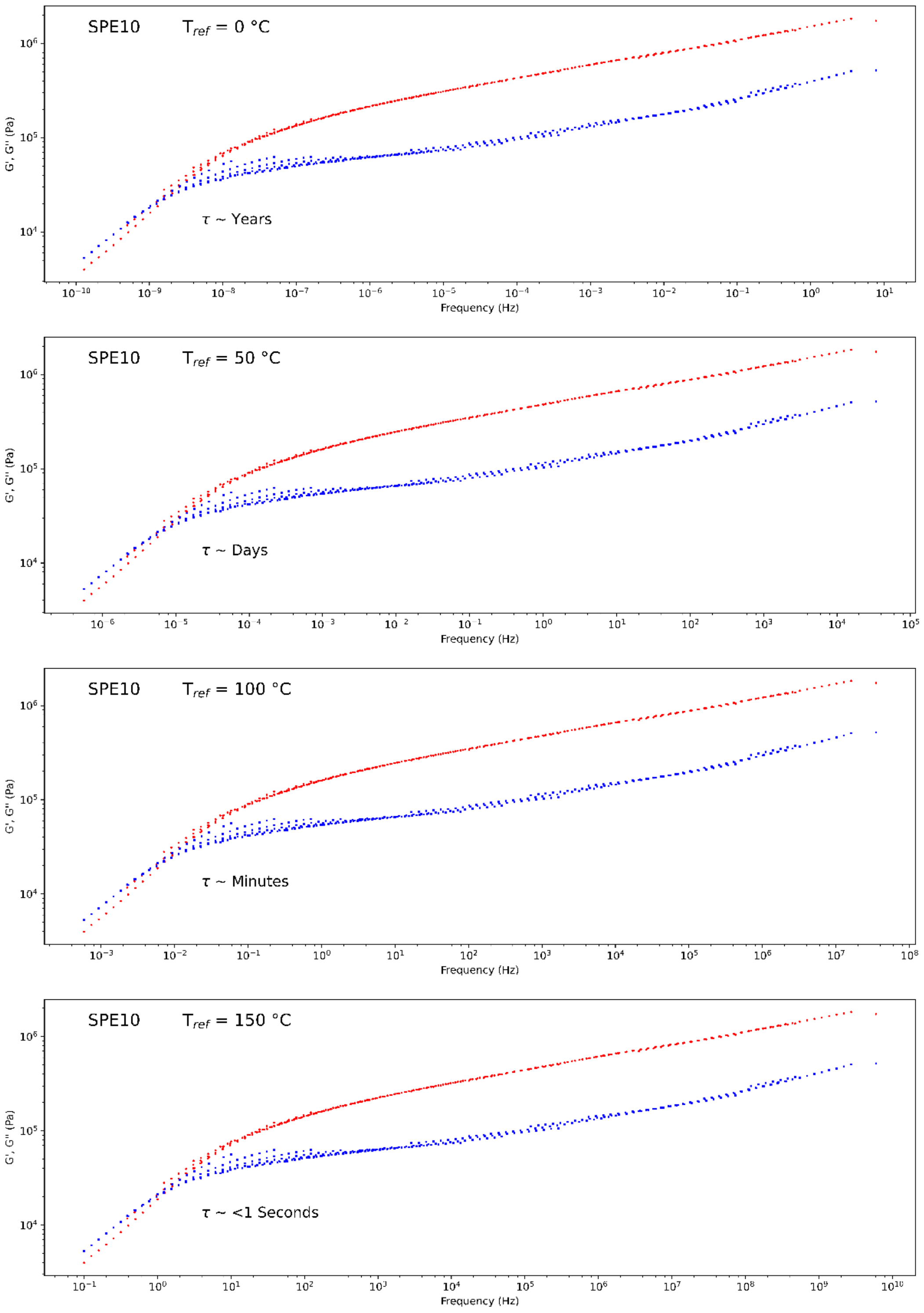

Figure S 107 Master curves of **SPE10** at a reference temperature (Tref) of 0, 50, 100, 150 °C, prepared by the TTS for G' and G'' values obtained at from frequency sweeps at 10 °C intervals from 0 to 150 °C, frequency sweeps were performed between 0.1 to 10 Hz at an applied strain of 0.1%.

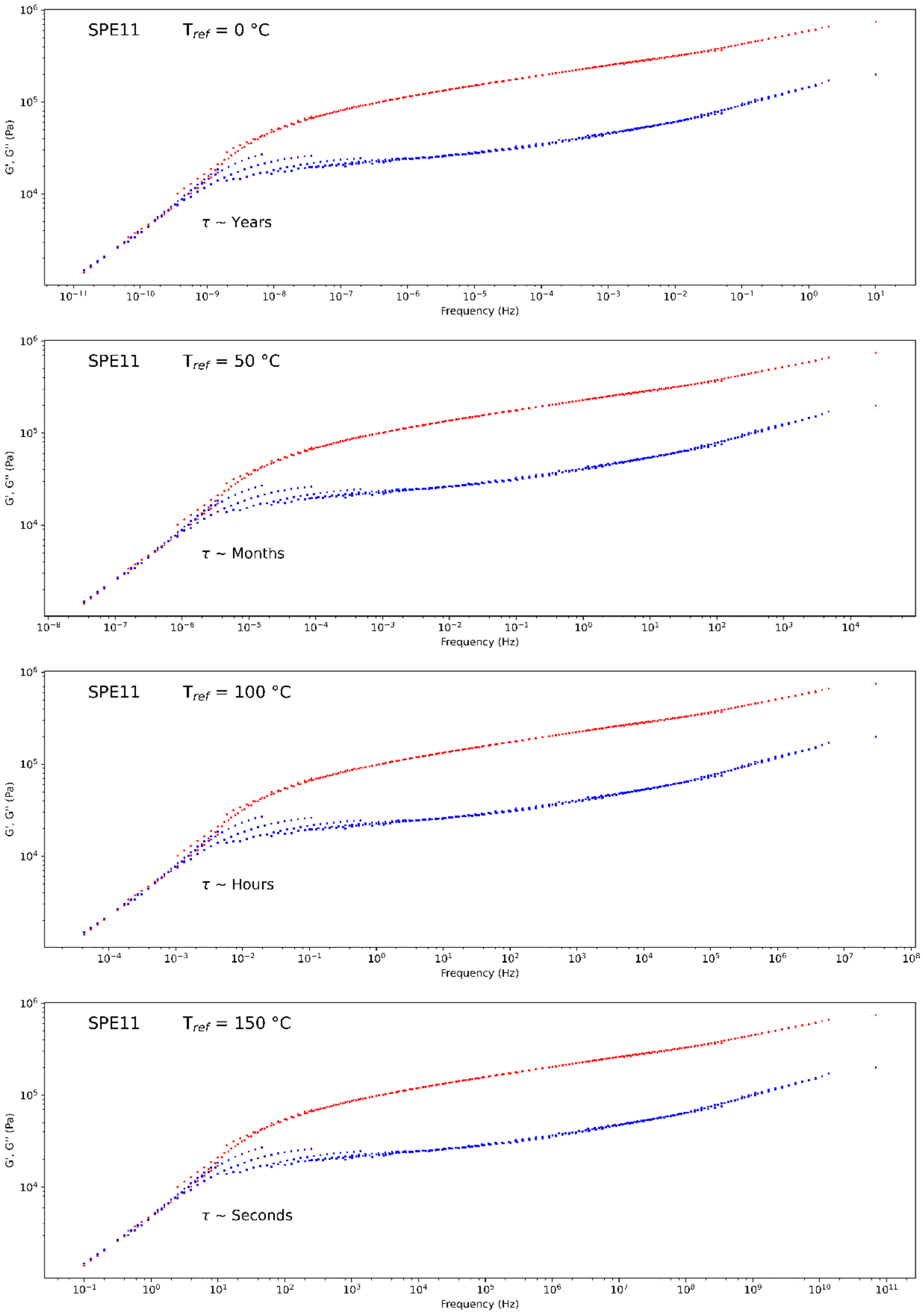

Figure S 108 Master curves of **SPE11** at a reference temperature (Tref) of 0, 50, 100, 150 °C, prepared by the TTS for G' and G'' values obtained at from frequency sweeps at 10 °C intervals from 0 to 150 °C, frequency sweeps were performed between 0.1 to 10 Hz at an applied strain of 0.1%.

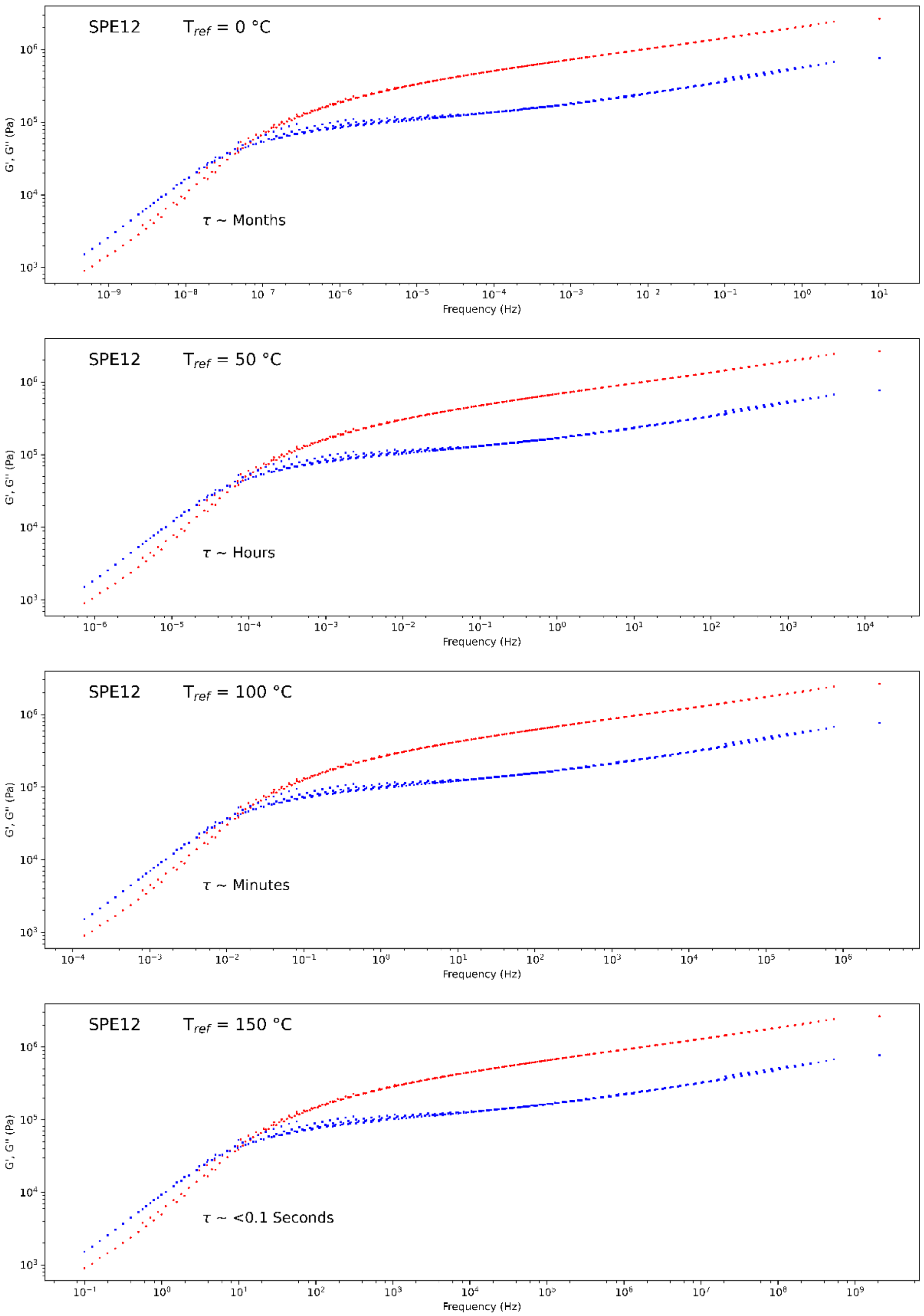

Figure S 109 Master curves of **SPE12** at a reference temperature (Tref) of 0, 50, 100, 150 °C, prepared by the TTS for G' and G'' values obtained at from frequency sweeps at 10 °C intervals from 0 to 150 °C, frequency sweeps were performed between 0.1 to 10 Hz at an applied strain of 0.1%.

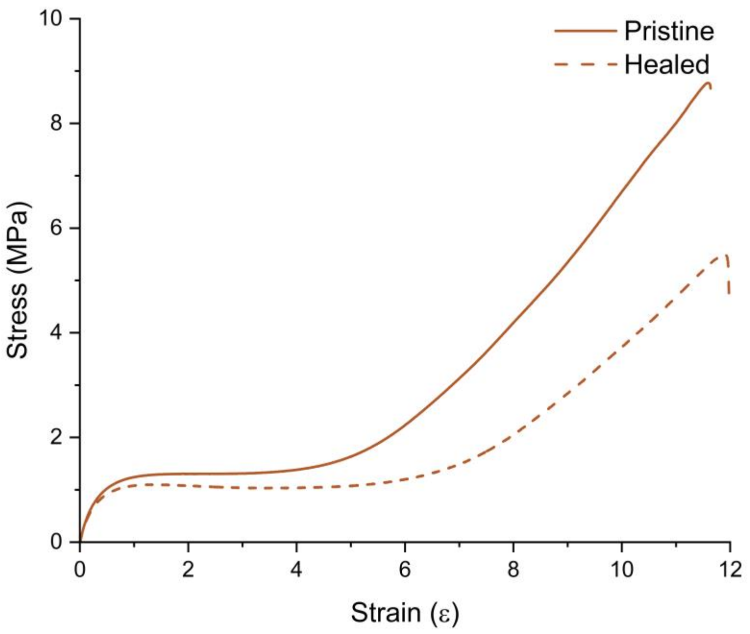

Figure S 110 Representative stress-strain curves of the pristine and healed **SPE1**.

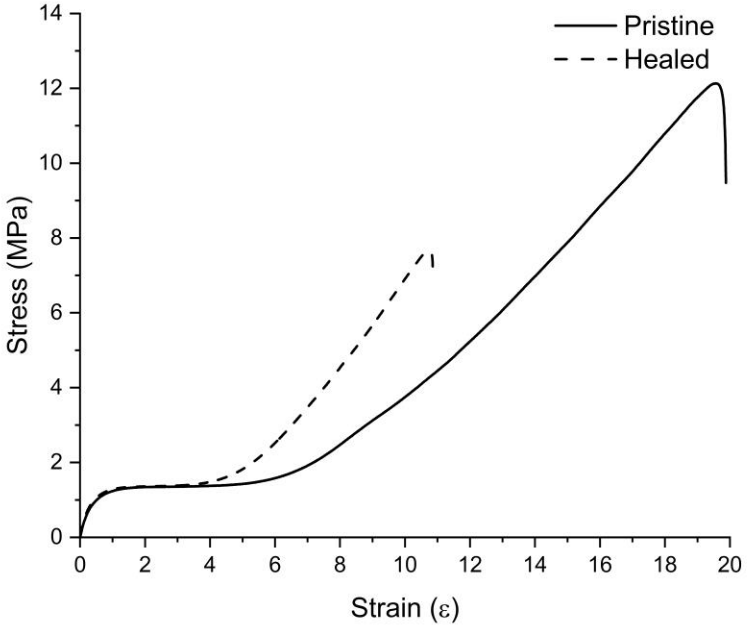

Figure S 111 Representative stress-strain curves of the pristine and healed **SPE2**.

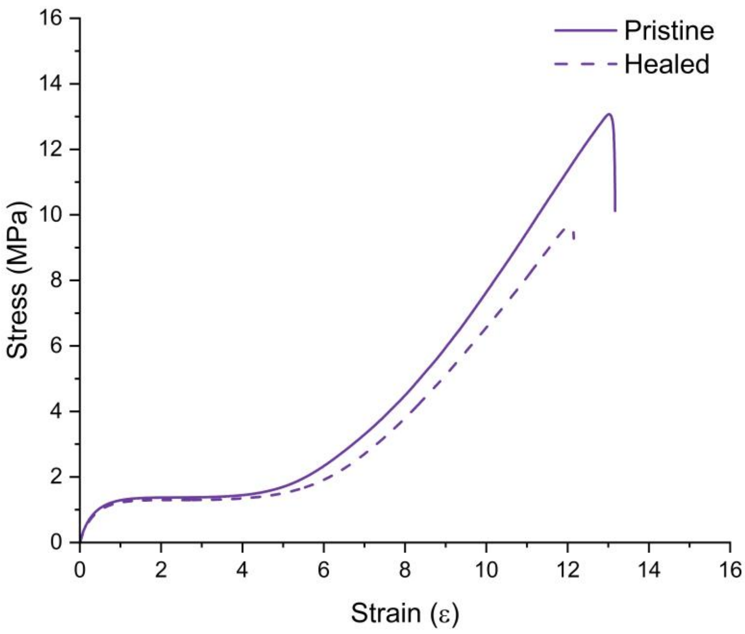

Figure S 112 Representative stress-strain curves of the pristine and healed **SPE3**.

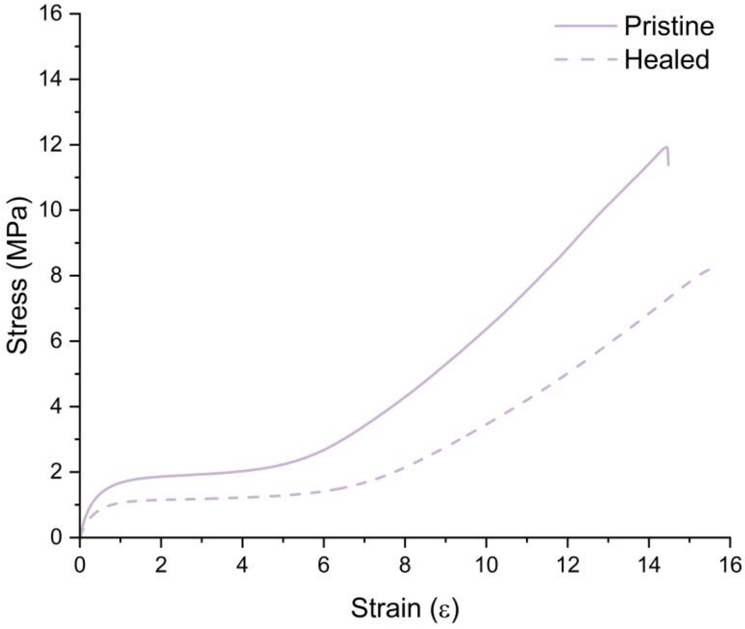

Figure S 113 Representative stress-strain curves of the pristine and healed **SPE4**.

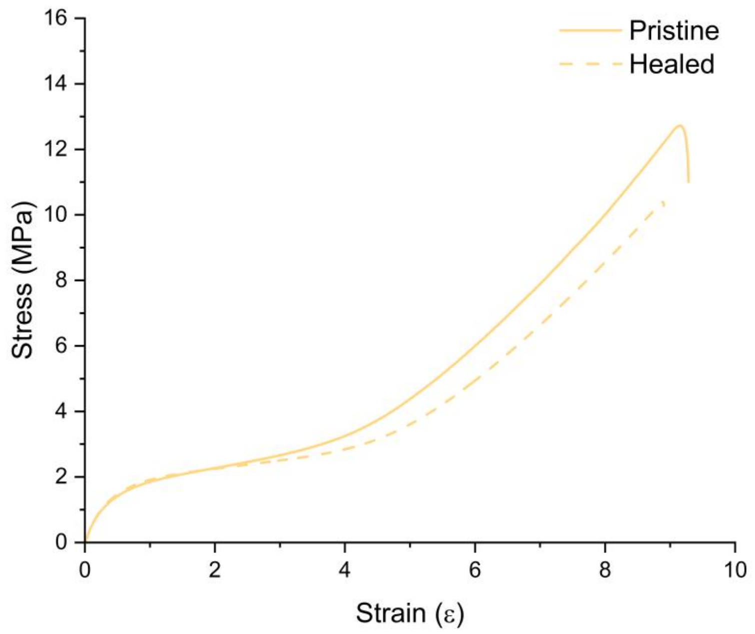

Figure S 114 Representative stress-strain curves of the pristine and healed **SPE5**.

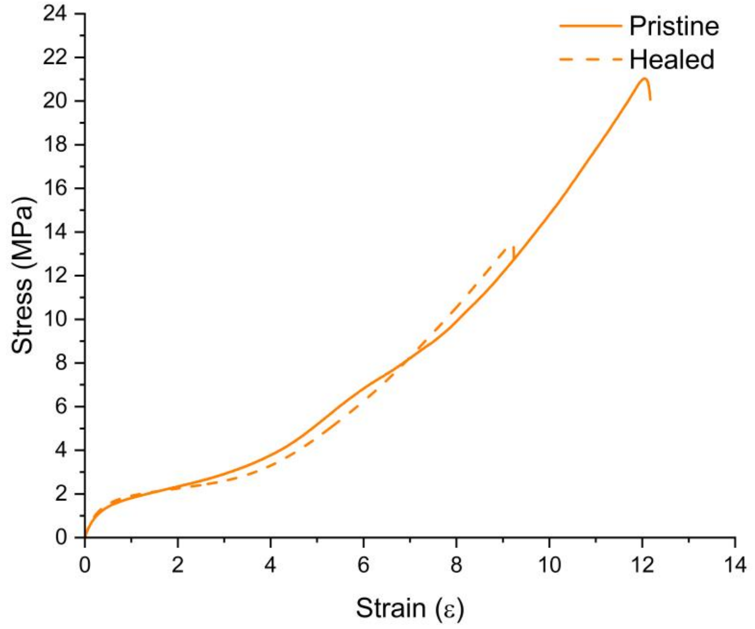

Figure S 115 Representative stress-strain curves of the pristine and healed **SPE6**.

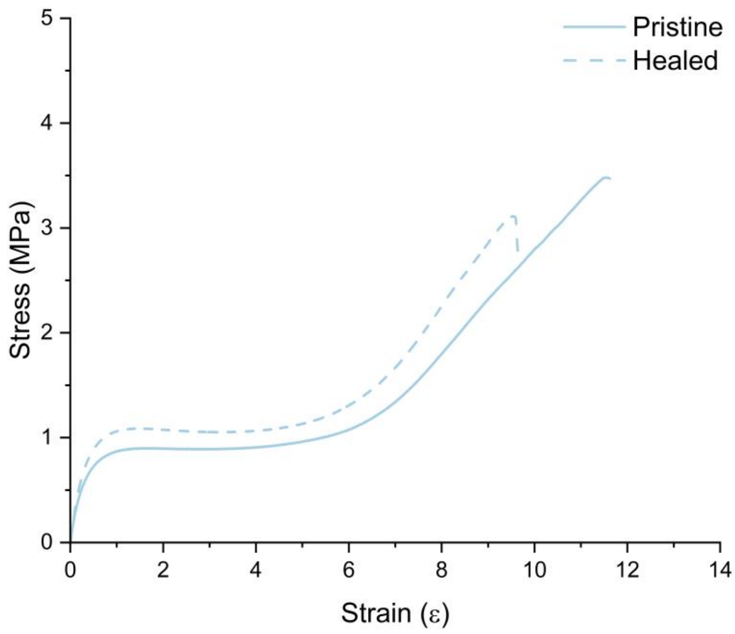

Figure S 116 Representative stress-strain curves of the pristine and healed **SPE7**.

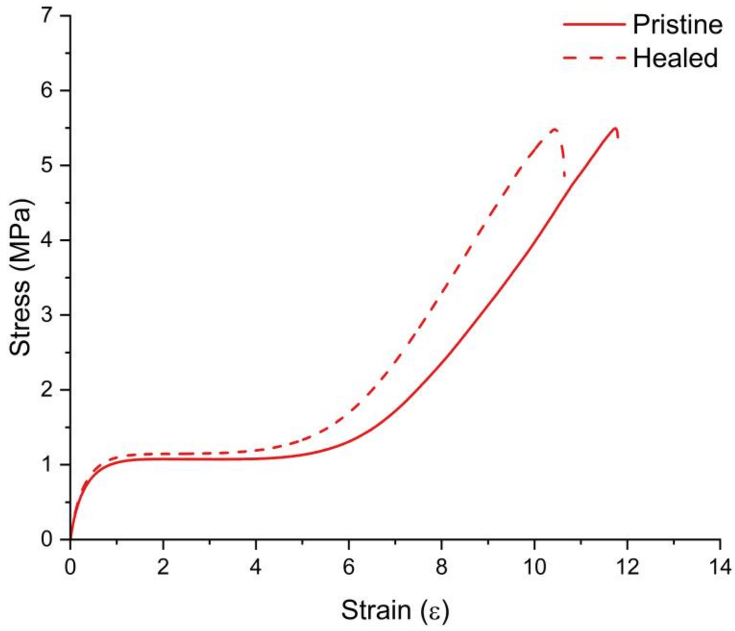

Figure S 117 Representative stress-strain curves of the pristine and healed **SPE8**.

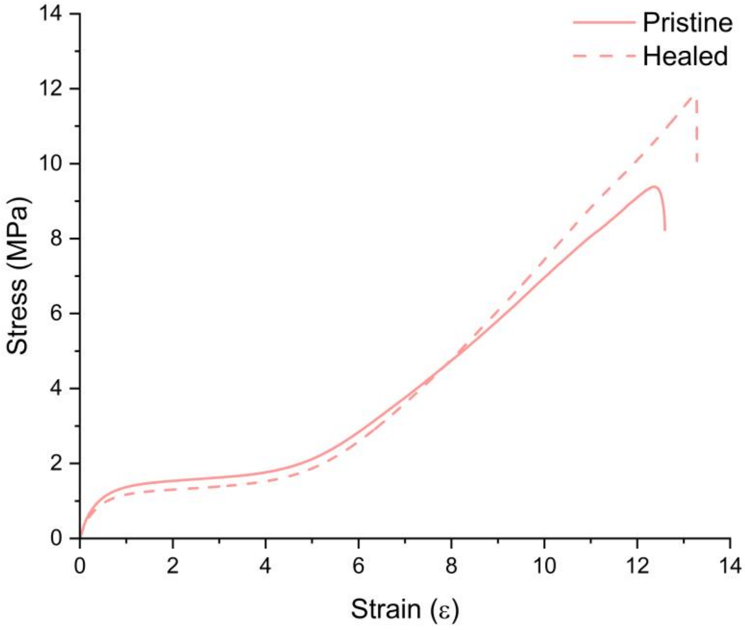

Figure S 118 Representative stress-strain curves of the pristine and healed **SPE9**.

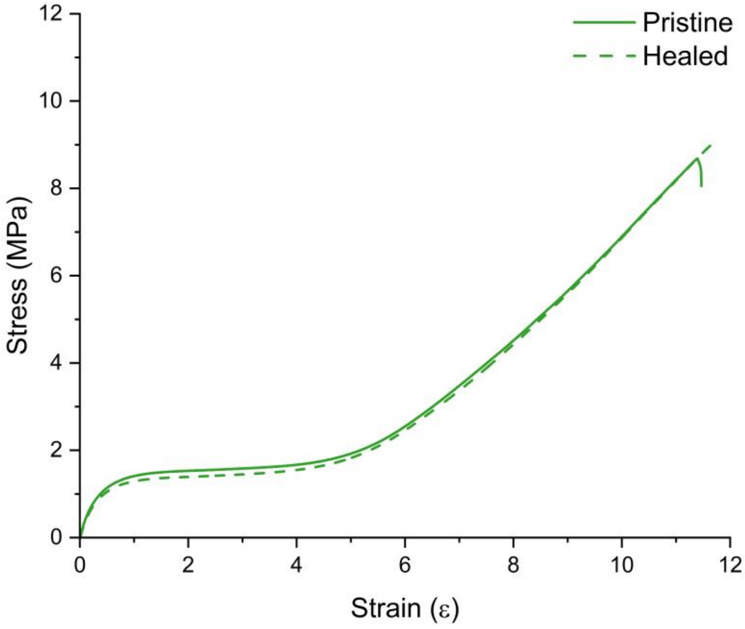

Figure S 119 Representative stress-strain curves of the pristine and healed **SPE10**.

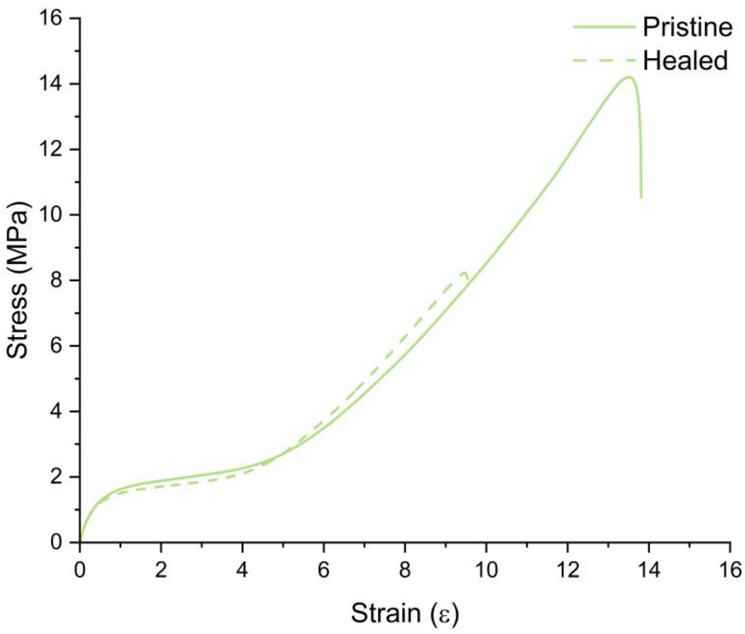

Figure S 120 Representative stress-strain curves of the pristine and healed **SPE11**.

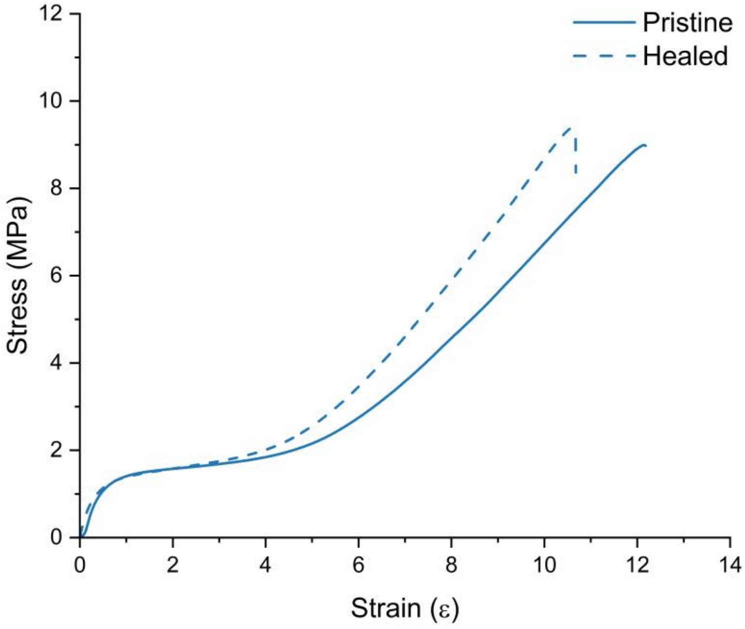

Figure S 121 Representative stress-strain curves of the pristine and healed **SPE12**.

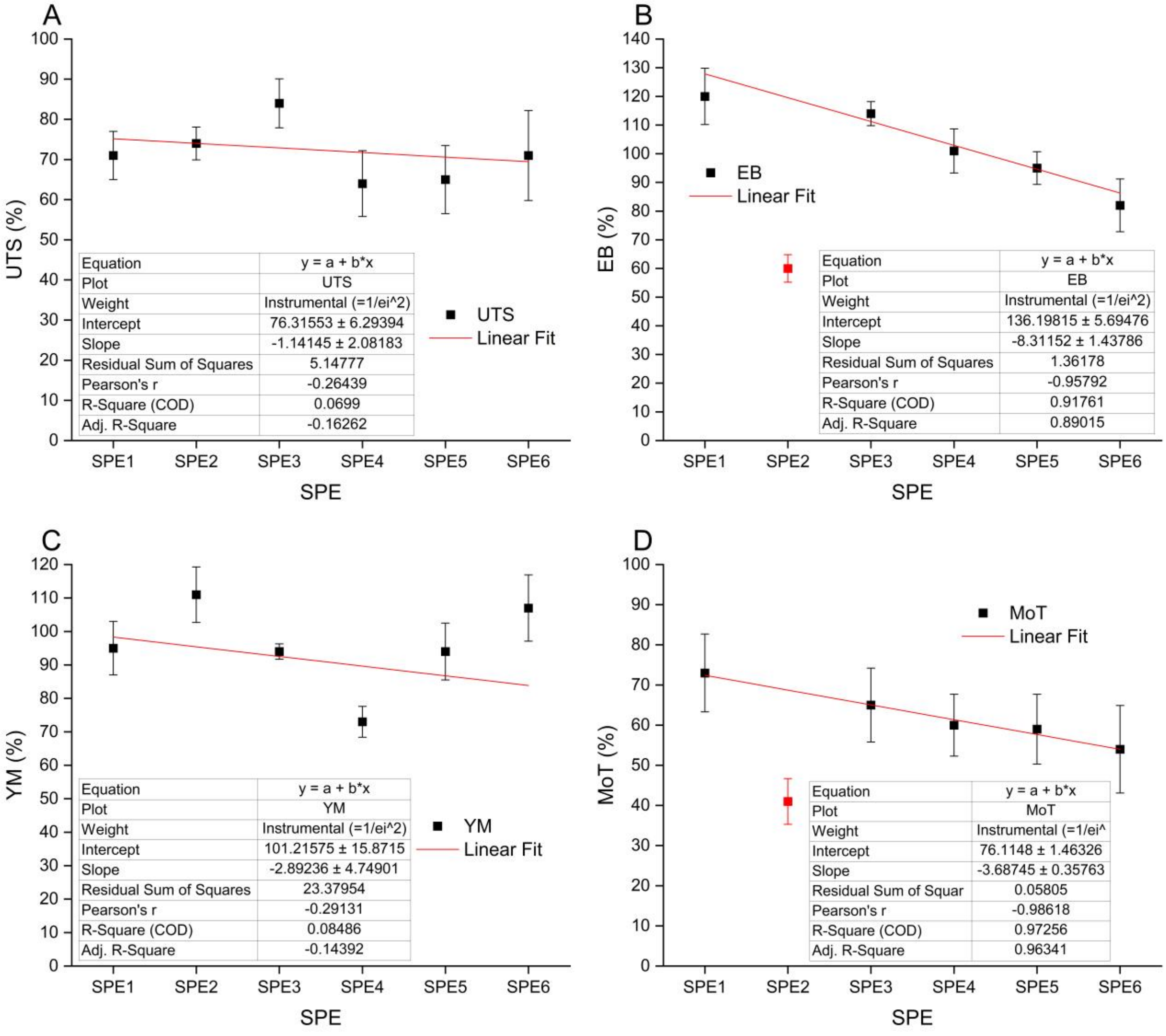

Figure S 122 Comparison of healing efficiencies of **SPE1**-**6** for **A** ultimate tensile strength (UTS), **B** elongation at break (EB), **C** Young's Modulus (YM), and **D** modulus of toughness (MoT).

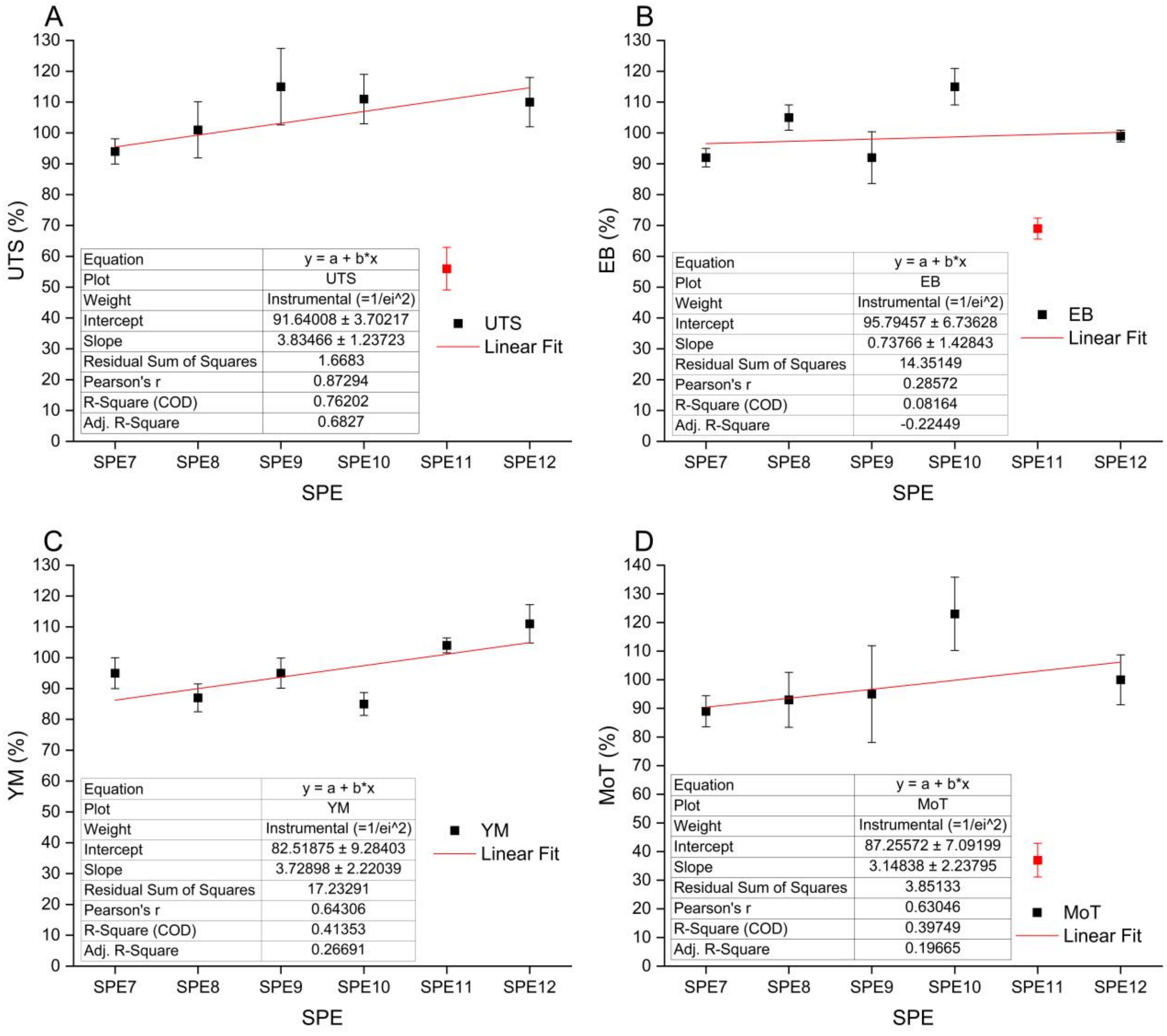

Figure S 123 Comparison of healing efficiencies of **SPE7**-**12** for **A** ultimate tensile strength (UTS), **B** elongation at break (EB), **C** Young's Modulus (YM), and **D** modulus of toughness (MoT).

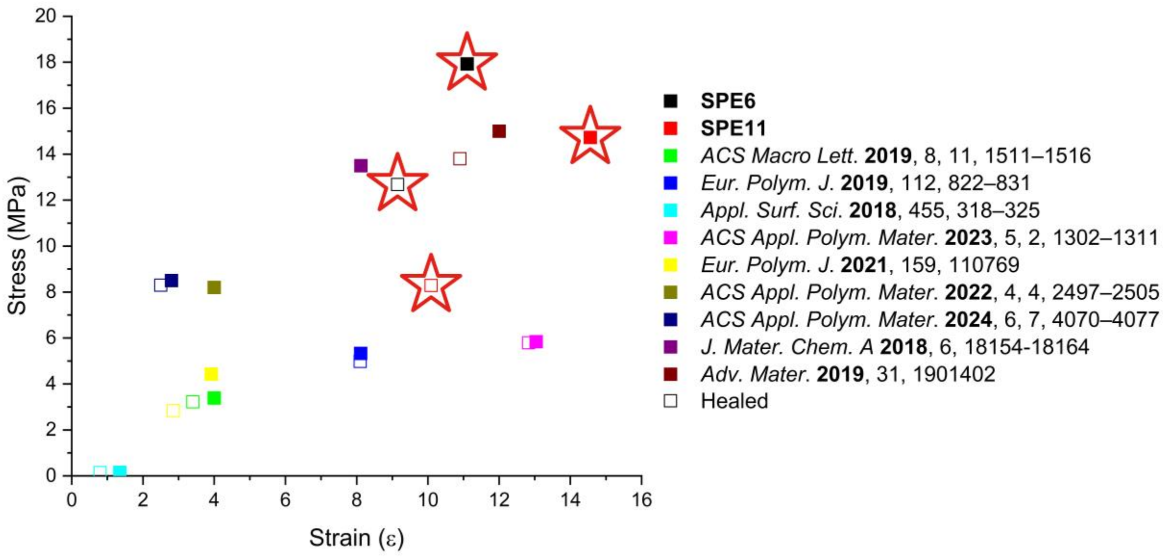

Figure S 124 Comparison of pristine and healed ultimate tensile strength and elongation at break for **SPE6** and **SPE11** with literature self-healing polyurethanes. [1–9]

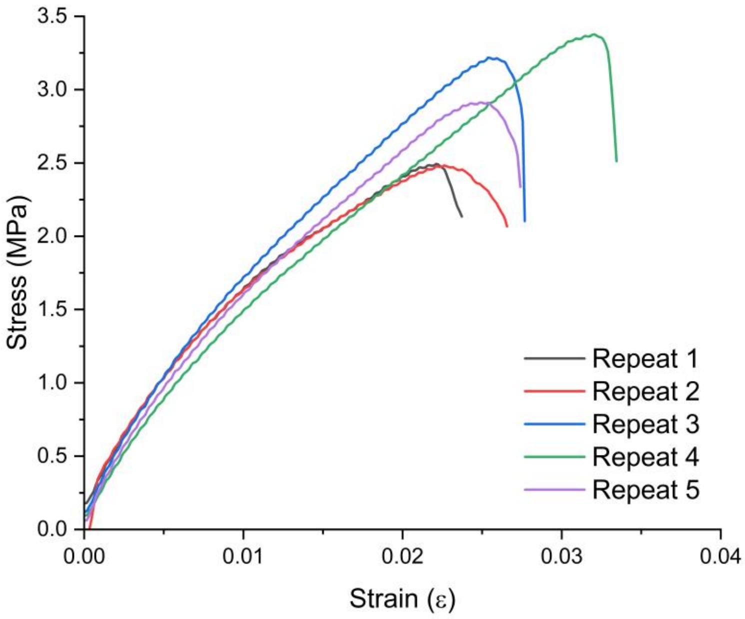

Figure S 125 Lap Shear adhesion of **SPE12** on Aluminium.

References

(1) Li, X.; Yu, R.; He, Y.; Zhang, Y.; Yang, X.; Zhao, X.; Huang, W. Self-Healing Polyurethane Elastomers Based on a Disulfide Bond by Digital Light Processing 3D Printing. *ACS Macro Lett.* **2019**, *8*, 1511–1516.

(2) Chang, K.; Jia, H.; Gu, S.-Y. A Transparent, Highly Stretchable, Self-Healing Polyurethane Based on Disulfide Bonds. *Eur. Polym. J.* **2019**, *112*, 822–831.

(3) Liu, J.; Ma, X.; Tong, Y.; Lang, M. Self-Healing Polyurethane Based on Ditelluride Bonds. *Appl. Surf. Sci.* **2018**, *455*, 318–325.

(4) Song, Y.; Li, J.; Song, G.; Zhang, L.; Liu, Z.; Jing, X.; Luo, F.; Zhang, Y.; Zhang, Y.; Li, X. Self-Healing Polyurethane Elastomers with High Mechanical Properties Based on Synergistically Thermo-Reversible and Quadruple Hydrogen Bonds. *ACS Appl. Polym. Mater.* **2023**, *5*, 1302–1311.

(5) Zhou, X.; Wang, H.; Li, S.; Liu, M. Synthesis and Application of Self-Healing Elastomers with High Healing Efficiency and Mechical Properties Based on Multi-Healing Systems. *Eur. Polym. J.* **2021**, *159*, 110769.

(6) Ma, J.; Lee, G.-H.; Kim, J.-H.; Kim, S.-W.; Jo, S.; Kim, C. S. A Transparent Self-Healing Polyurethane–Isophorone-Diisocyanate Elastomer Based on Hydrogen-Bonding Interactions. *ACS Appl. Polym. Mater.* **2022**, *4*, 2497–2505.

(7) Li, F.; Wang, X.; Zuo, J.; Chen, C.; Chen, J.; Zhu, J.; Ying, W. Bin. Oxime-Urethane-Based Self-Healing Polyurethane for Achieving Complex Structures via 3D Printing. *ACS Appl. Polym. Mater.* **2024**, *6*, 4070–4077.

(8) Fu, D.; Pu, W.; Wang, Z.; Lu, X.; Sun, S.; Yu, C.; Xia, H. A Facile Dynamic Crosslinked Healable Poly(Oxime-Urethane) Elastomer with High Elastic Recovery and Recyclability. *J. Mater. Chem. A* **2018**, *6*, 18154–18164.

(9) Zhang, L.; Liu, Z.; Wu, X.; Guan, Q.; Chen, S.; Sun, L.; Guo, Y.; Wang, S.; Song, J.; Jeffries, E. M.; He, C.; Qing, F.; Bao, X.; You, Z. A Highly Efficient Self-Healing Elastomer with Unprecedented Mechanical Properties. *Adv. Mater.* **2019**, *31*, 1901402.
